# Morpholine as a privileged scaffold for neurodegenerative disease therapeutics

**DOI:** 10.1039/d6ra00100a

**Published:** 2026-03-04

**Authors:** Saranya Kattil Parmbil, Sunil Kumar, T. M. Rangarajan, Della Grace Thomas Parambi, Naseer Maliyakkal, Anél Petzer, Jacobus P. Petzer, Bijo Mathew

**Affiliations:** a Department of Pharmaceutical Chemistry, Amrita School of Pharmacy, Amrita Vishwa Vidyapeetham, AIMS Health Sciences Campus Kochi 682 041 India bijomathew@aims.amrita.edu bijovilaventgu@gmail.com; b Department of Chemistry, Sri Venkateswara College, University of Delhi New Delhi - 110 021 India; c Department of Pharmaceutical Chemistry, College of Pharmacy, Jouf University Sakaka Aljouf 72341 Saudi Arabia; d Department of Anesthesia and Operations, College of Applied Medical Sciences, King Khalid University Khamis Mushait Kingdom of Saudi Arabia; e Centre of Excellence for Pharmaceutical Sciences, North-West University Potchefstroom 2520 South Africa jacques.petzer@nwu.ac.za

## Abstract

Morpholine, a six-membered heterocyclic ring containing oxygen and nitrogen, is increasingly recognized in medicinal chemistry for its diverse pharmacological properties. Morpholine derivatives have shown promise as enzyme inhibitors, neurotransmitter modulators, and receptor agonists, making them candidates for treating neurodegenerative and neuropsychiatric disorders. The importance of this study lies in elucidating the therapeutic significance of morpholine in central nervous system (CNS) drug development, particularly in its role in inhibiting monoamine oxidases (MAO-A and MAO-B) and cholinesterases (AChE and BChE) and targeting the norepinephrine and dopamine pathways. Systematic searches of peer-reviewed literature were conducted to evaluate the structural significance and pharmacological potential of morpholine-based compounds. The findings reveal that morpholine derivatives exhibit promising efficacy and selectivity, often outperforming conventional drugs in specific contexts. These discoveries point to the possibility of morpholine as a valuable scaffold in CNS drug discovery due to its balanced lipophilic–hydrophilic profile and enhanced blood–brain barrier permeability. The implications of these findings are significant, and they call for future research to focus on optimizing morpholine derivatives to improve selectivity, efficacy, and safety, thereby expanding their therapeutic potential in CNS applications. Overall, this study emphasizes the importance of morpholine derivatives in advancing CNS therapeutics and their potential role in addressing unmet medical needs in neuropsychiatric disorder treatments.

## Abstract 2

The review emphasizes the importance of morpholine derivatives in advancing CNS therapeutics and their potential role in addressing unmet medical needs in neuropsychiatric disorder treatments.

## Graphical abstract

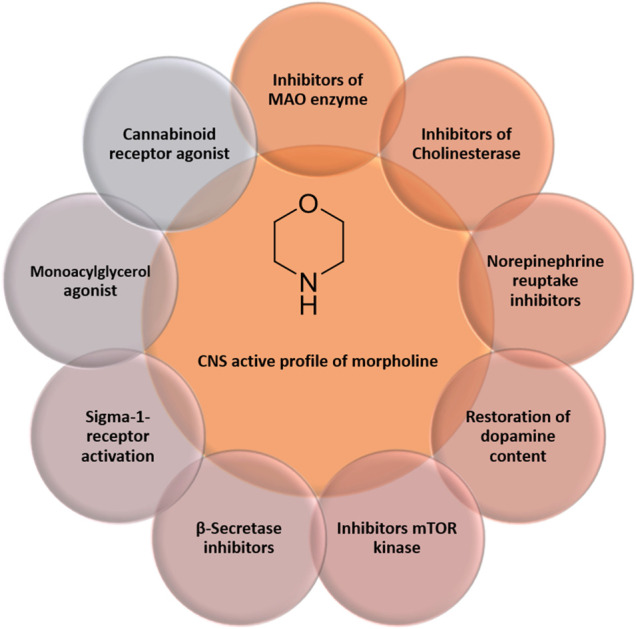

## 1 Introduction

**Fig. 1:**
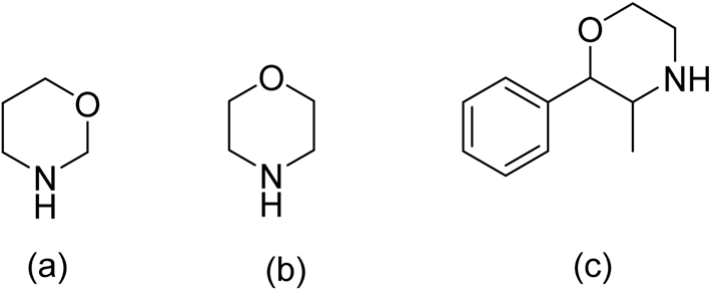
(a) Structure of 1,3-oxazinane; (b) structure of 1,4-oxazinane; (c) structure of preludin.

Neurodegenerative disorders such as Alzheimer's and Parkinson's disease continue to pose major therapeutic challenges due to their complex pathology and the limited ability of drugs to cross the blood–brain barrier (BBB). One of the central hurdles in CNS drug development is achieving sufficient brain penetration without compromising safety or pharmacokinetic stability.^1–4^ In this context, morpholine has become an essential heterocyclic scaffold since it can be employed in numerous ways, including structurally, physically, and chemically. Based on where the oxygen atom is in relation to the nitrogen atom, the resulting structures are called 1,3-oxazinane and 1,4-oxazinane (Fig. 1a and b). The second one is also called morpholine or tetrahydro-1,4-oxazine, which is a common part of bioactive molecules and drugs.^2,4,5^ Perludin was the first marketed drug that contained morpholine, introduced for the treatment of obesity in 1955 (Fig. 1c). Two years later, several morpholine analogs were developed with a wide range of therapeutic properties.^6^

The morpholine ring has a number of built-in features that make it useful for designing drugs for the central nervous system. Its balanced lipophilic–hydrophilic profile and lower pKa make it easier for molecules to cross the BBB. The spatial arrangement of its weakly basic nitrogen and oxygen enhances aqueous solubility and fine-tunes molecular polarity, which allows it to access CNS-relevant chemical space.^2–4^ Also, the electron-deficient nature of the ring which is caused by the negative inductive effect of oxygen, affects the pharmacokinetic and pharmacodynamic properties. The oxygen atom also helps in forming hydrogen bonding and the ring's hydrophobic surface encourages complementary interactions in protein binding pockets, which makes target engagement better.^2,3,6,7^ Along with these electronic features, the morpholine scaffold maintains a good balance between hydrophilicity and lipophilicity across various substitution patterns. This helps in formulation flexibility and transport across the blood–brain barrier. The ability to change shape between chair and skew-boat conformations makes it useful for positioning pharmacophoric groups. Morpholine-containing drugs mostly show better metabolic profiles. For example, CYP3A4 converts morpholine into non-toxic metabolites, which will help in efficient clearance and reduce toxicity.^2–4,6^ The combination of these characteristics has established morpholine as a common structural element in CNS drugs that inhibit monoamine oxidases and cholinesterases and affect neurotransmitter transporters and kinases and receptors.^3,4,6^

Scientists have not yet established the specific ways morpholine impacts CNS pharmacology, although it provides various advantages. Notwithstanding the fact that several reviews have already pointed out morpholine as a privileged heterocyclic core in medicinal chemistry, the majority of the literature available gives a general perspective that spans across various therapeutic areas, without a specific focus on central nervous system (CNS) drug discovery. Specifically, there is a lack of a systematic compilation of CNS-related molecular targets such as monoamine oxidases, cholinesterases, mTOR, β-secretase, Sigma-1 receptor, MAGL, and neurotransmitter receptors, together with structure–activity relationships, potency profiles, computational perspectives, and ADMET aspects.

The current review fills this void by providing a CNS-focused, target-driven perspective on morpholine derivatives published between 2019 and 2025. By making a correlation between structural variations and biological potency (IC_50_/*K*_i_), binding affinity, computational stability, and CNS-related ADMET properties, this review aims to provide a comprehensive platform that identifies the growing importance of morpholine as a multitarget scaffold in neurodegenerative and neuropsychiatric drug discovery. Additionally, the translational aspects and development limitations are critically reviewed to outline future directions for morpholine-based CNS therapeutics.

### 1.1 Morpholine chemistry and synthesis

**Scheme 1:**
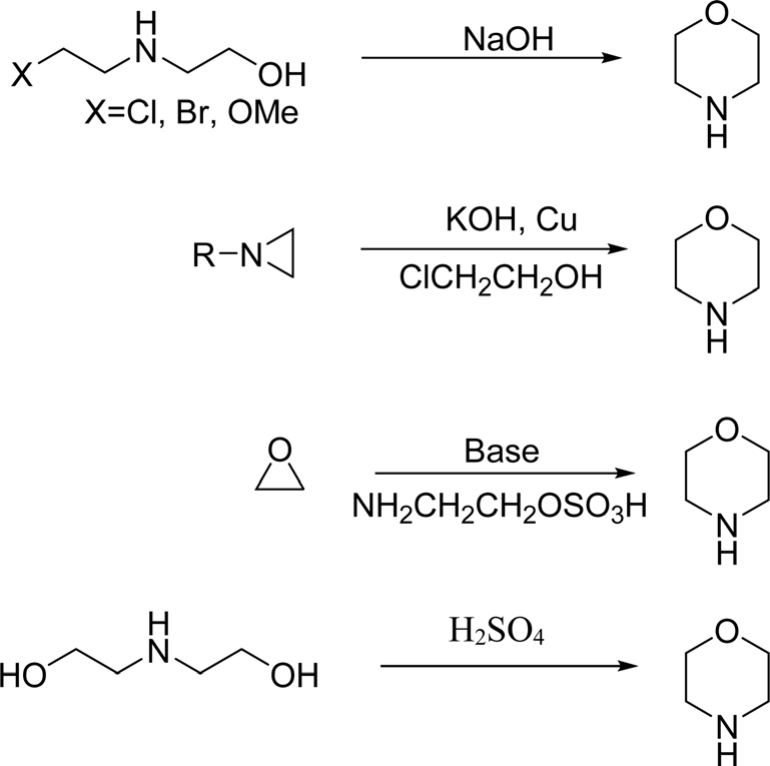
Conventional methods of synthesis of substituted morpholines.

**Scheme 2:**
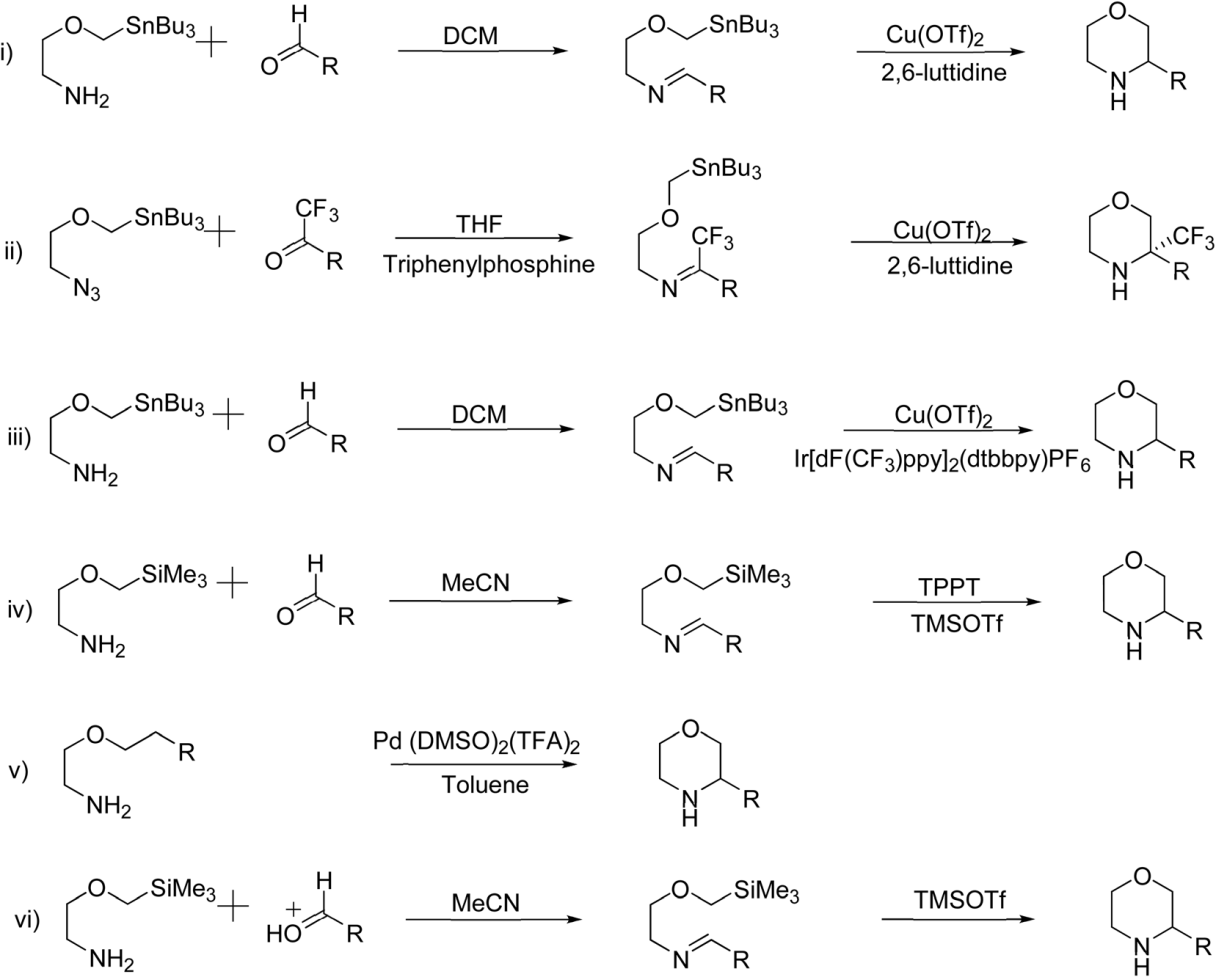
Recent methods for synthesis of substituted morpholines from organotin and organosilane compounds.

Several conventional methods have reported the formation of the morpholine ring with varying yields and practicality. Scheme 1 outlines the conventional methods, emphasizing their drawbacks, which include expensive reagents, extreme reaction conditions, and poor yields. Researchers have developed superior catalysts and approaches to overcome these challenges, as shown in Scheme 2, that enable more effective morpholine synthesis.^2,4,6^ A concise method for synthesizing morpholine using amino alcohols or diamines with substituted α-phenylvinylsulfonium salts has been reported (Scheme 2(i)). This approach, which requires no regulated temperature and pressure, offers excellent yields when utilising a base and dichloromethane (DCM) as solvent.^4^

The Bode group created the Stannyl Amine (SnA) protocol, which is a remarkable recent breakthrough in the synthesis of morpholine. Cu-II mediates a radical-based stoichiometric cyclisation between an aldehyde and SnA reagents (2-[(tributylstannyl)methoxy]ethan-1-amine or its derivatives) (Scheme 2(ii)). It is captivating owing to its wide range of substrates, consistently excellent yields, and the elimination of the necessity for protecting groups. Furthermore, the tin reagents in this protocol are readily available and show a respectable level of stability in both air and moisture. In addition to the previously reported stoichiometric variations of the SnA protocol, the same group subsequently introduced the catalytic variants. In these scenarios, an iridium catalyst is used in a continuous flow reaction under irradiation, or a ligand is conjugated with a catalytic quantity of Cu-II (Scheme 2(iii)). These SnA protocol variants often result in reliable synthetic channels that are impervious to moisture and air, and they have a variety of uses. Despite producing less effective results than copper catalysts, the irradiation approaches make scale-up and workup procedures easier. The Silicon Amine Protocol (SiAP), which uses silicon reagents rather than tin reagents, has been developed by the Bode group as an alternative approach to organotin reagents. Blue light and the organic photoredox catalyst 2,4,6-triphenylpyrylium tetrafluoroborate are necessary for this reaction (Scheme 2(iv)). The compliance SiA protocol with flow conditions is another benefit, in addition to its ability to remove hazardous tin byproducts.^2,6^

The Wacker-type aerobic reaction described here involves a Pd-catalyzed oxidative cyclization where Lu and colleagues employed a base-free Pd(DMSO)_2_(TFA)_2_ catalyst to cyclize alkenes into morpholine and related N-heterocycles efficiently (Scheme 2(v)). This method is eco-friendly and is bolstered by its moderate conditions and absence of further bases. It is also malleable because it enables oxidative acylation of C–H bonds. In contrast to conventional oxidative procedures, this recent innovation offers great selectivity and improved efficiency by using an aerobic oxidant to drive the process.^4^

A straightforward approach for synthesizing morpholine rings, as this reliably synthesises morpholine under moderate conditions without the need for high temperatures or additional catalysts. The amine group on 2-(trimethylsilyl)methoxyethanamine presumably condenses with the carbonyl group of the aldehyde, which leads to the formation of an imine intermediate. The trimethylsilyl (TMS) group serves as a protective agent, stabilizing the oxygen on the methoxy group and aiding in the cyclization process. After the imine is formed, the process yields a six-membered morpholine ring by intramolecular cyclisation (Scheme 2(vi)). Once the TMS group has been eliminated, the final substituted morpholine product may be obtained.^2,4^

**Scheme 3:**
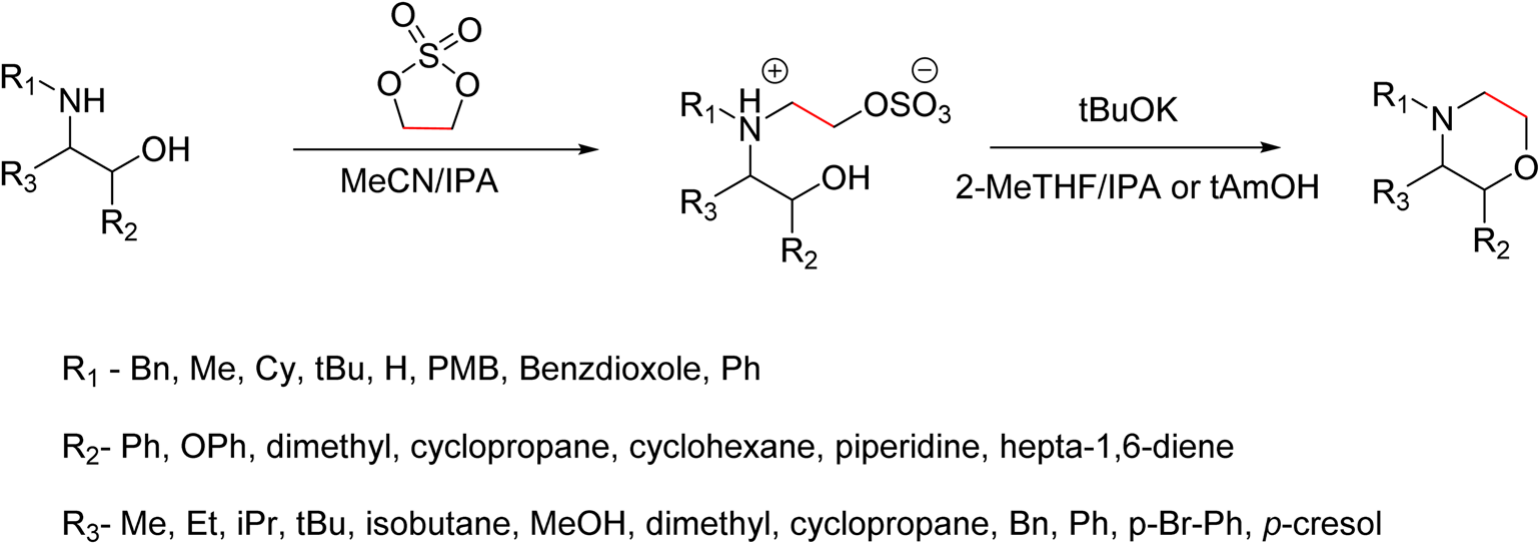
Green synthesis of substituted morpholines by monoalkylation of amines.

A recent advancement in the green and scalable synthesis of morpholines was reported by K. Ortiz *et al.* (Scheme 3). They synthesised morpholine from substituted 1,2-aminoalcohol using inexpensive reagents like ethylene sulfate and tBuOK, and thereby addressing limitations of traditional methods. In the conventional synthesis of morpholine from 1,2-aminoalcohol, the usually used reagents are chloroacetyl chloride and metal hydrides. And this will generate a morpholinone intermediate, which makes this conventional method complex and creates more waste and does not follow the green principles. However, this new approach adopts environmentally friendly conditions and inexpensive reagents. The synthesis begins with the selective monoalkylation of primary or secondary 1,2-aminoalcohols using ethylene sulfate. The reaction proceeds *via* an SN2 mechanism and results in zwitterionic intermediates, which are obtained as crystalline solids. The selectivity for monoalkylation is preferable to conventional alkylating agents such as dimethyl sulfate or ethylene oxide, which frequently result in overalkylation. Monoalkylation selectivity is influenced by steric hindrance around the nitrogen centre as well as the intrinsic reactivity of ethylene sulphate. They found that solvents like acetonitrile in combination with small amounts of water or isopropanol as cosolvents had improved the conversion of the product and facilitated its isolation. In the subsequent step, intramolecular cyclization of the zwitterionic intermediates with potassium *tert*-butoxide (tBuOK) leads to the formation of morpholines with good yield. In this cyclization they had been introduced green solvents like 2-MeTHF/IPA or *tert*-amyl alcohol and the reaction is carried out at moderate heat (40–60). The use of a wide range of substrates like α- or β-substituted amino alcohols and a variety of N-substituents enables the synthesis of structurally diverse morpholines. The practicality of this method is demonstrated by the successful large-scale synthesis of morpholine derivatives, one-pot conversion protocols, and its application in the synthesis of pharmaceutically relevant morpholine derivatives like reboxetine, phendimetrazine, and fingolimod. Overall, this method involves the use of easily accessible reagents, reduces the generation of waste, avoids the use of toxic solvents, and it aligns with the principles of green chemistry. It represents a significant advancement in the sustainable synthesis of morpholine-based scaffolds, especially where selective monoalkylation of primary amines is desired.^8^

### 1.2 FDA-approved drugs containing the morpholine ring

**Fig. 2:**
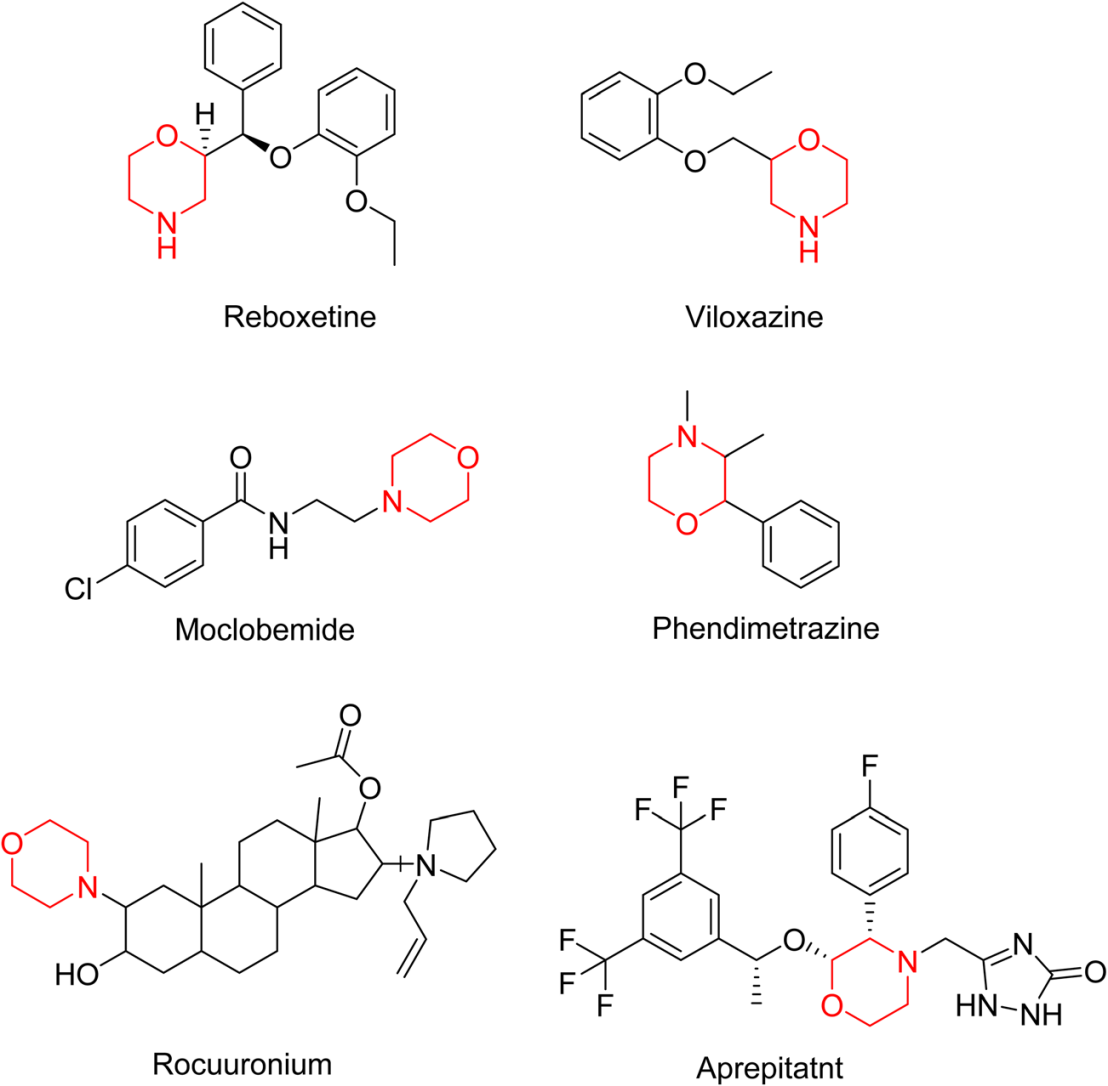
Structure of FDA-approved CNS drugs containing a morpholine ring.

Morpholine's frequent appearance in numerous FDA-approved drugs reflects its pharmaceutical importance. As of the 2003 World Drug Index database, more than 100 pharmaceutical drugs incorporate a morpholine ring in their structure, including several FDA-approved CNS drugs. Notable examples include reboxetine, viloxazine, moclobemide, phendimetrazine, rocuronium, and aprepitant (Fig. 2).^2–7^

Reboxetine, which is a selective norepinephrine reuptake inhibitor, features morpholine rings in its structure and is primarily utilized as an antidepressant as well as an anti-anxiety medication.^4,5,7,9,10^ Due to its high electron density, reboxetine undergoes metabolism *via* the cytochrome P450 enzyme, which involves the cleavage of the morpholine ring.^4,9^ Similar to reboxetine, viloxazine is a potent antidepressant functioning as a selective norepinephrine reuptake inhibitor. The extended-release formulation of viloxazine has recently received FDA approval for the management of ADHD. This drug contains a morpholine ring, which significantly influences its pharmacokinetic and pharmacodynamic properties. Viloxazine undergoes extensive metabolism through glucuronidation of hydroxyl groups, cleavage of the morpholine chain, and *N*-methylation of the morpholine ring.^4,11–16^ Moclobemide is a morpholine-containing drug that reversibly inhibits monoamine oxidase A. This drug is primarily used to treat depression and social anxiety.^4–7^ The morpholine in moclobemide is substantially metabolised (half-life 1–3 hours) and provides a superior safety profile than non-reversible MAO inhibitors. MAO activity is completely recovered after 16–24 hours.^4,6^ Aprepitant is a selective neurokinin-1 (NK1) receptor antagonist used to treat chemotherapy-induced nausea and vomiting (CINV) and postoperative nausea and vomiting (PONV). Although morpholine is an integral part of this drug, it decreases the basicity while increasing the potency.^4,6^ Phendimetrazine is an anorectic drug. It inhibits human dopamine transporters. The active metabolite (phenmetrazine) competes with norepinephrine and dopamine for the same presynaptic transporter. The morpholine ring in the structure of phendimetrazine is crucial to its CNS function because it improves the drug's interaction with norepinephrine pathways, resulting in the suppression of hunger signals.^4,6^ Rocuronium is a non-depolarizing neuromuscular blocking drug used during anesthesia. It is a competitive antagonist for the postsynaptic nicotinic receptor. The morpholine group contributes to the effectiveness of rocuronium.^5,6^

## 2 CNS active profile of morpholine

### 2.1 Inhibitors of the MAO enzyme

Parkinson's disease is marked by the gradual degeneration of neurons in the brain, resulting in impaired movement. The two isoforms, MAO-A and MAO-B, differ biochemically in their specific substrates and inhibitors. MAO-B is found on the raphe nuclei of serotonergic neuronal cell bodies, and its inhibition leads to an increase in dopamine levels in patients with Parkinson's disease. To achieve a therapeutic effect, at least 80% of this enzyme must be inhibited. Reversible and irreversible inhibitors are the two categories of MAO-B inhibitors. Reversible inhibitors resemble MAO substrates in structure and bind to the enzyme's active site, though they metabolise at a slow rate. In contrast, irreversible inhibitors initially bind reversibly, after which they are oxidised by the FAD cofactor, rendering them unavailable for amine metabolism.^17^

Monoamine oxidases (MAOs) are crucial in deactivating biogenic amines in both central and peripheral tissues. They are found on the mitochondrial outer membrane in two isoforms, MAO-A and MAO-B. Alzheimer's and Parkinson's diseases are two neurodegenerative diseases that can be effectively managed by targeting these enzymes. MAOs catalyze the oxidative deamination of neurotransmitters, including dopamine, epinephrine, norepinephrine, and serotonin, resulting in the formation of hydroxyl radicals-reactive oxygen species that cause oxidative damage to brain tissues. By inhibiting MAOs, MAO inhibitors can raise neurotransmitter levels in the brain, offering neuroprotective effects.^5,7,17,18^

**Scheme 4:**
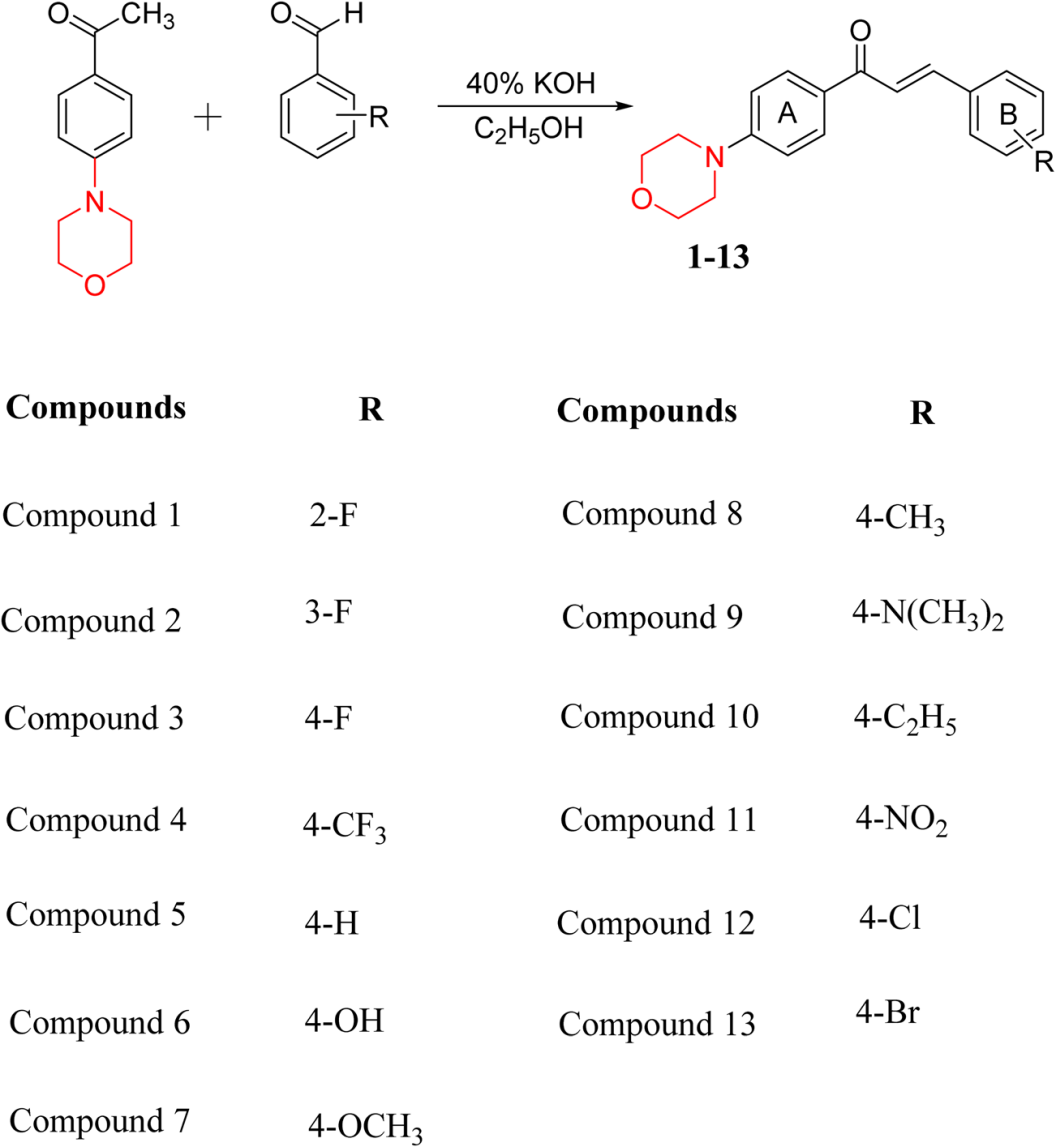
Synthesis of morpholine-substituted α,β-unsaturated ketones 1–13.

Mathew *et al.* developed four derivatives of morpholine-substituted α,β-unsaturated ketones (1–4) (Scheme 4) and discovered that they are highly selective MAO-B inhibitors possessing reversibility features. The most effective compounds in the series were morpholine-containing compound-1 and compound-2 with IC_50_ values of 0.14 ± 0.005 µM and 0.087 ± 0.008 µM, respectively, against hMAO-B. The position of the fluorine atom on ring B of the chalcone core varied among these compounds. The fluorine substitution at the *meta*-position showed a more potent MAO-B inhibitory effect than the *ortho* and *para*-positions of ring B of chalcones. Compound 2 reported a remarkably high selectivity index (SI) of 517.2 for MAO-B, indicating that this compound is a highly selective inhibitor of MAO-B. Additionally, the potency of compound 2 (0.087 µM) was greater than that of pargyline (0.097 µM), which is the standard hMAO-B irreversible inhibitor. Kinetics studies were carried out for these two lead compounds and found that they are competitive and reversible MAO-B inhibitors. Interestingly, all chalcones showed blood–brain barrier permeation in parallel artificial membrane permeation assay (PAMPA). *In-silico* studies revealed that compound 2 had π–π and hydrophobic interaction with key amino acid Tyr326, which is accountable for the biological response, and the molecular dynamics study concluded that compound 2-MAO-B complex is stable.^17^

R. Sasidharan *et al.* condensed several aromatic *para*-substituted aldehydes and 4-morpholine acetophenone to produce morpholine-containing α,β-unsaturated ketones (Scheme 4) in the presence of an alcoholic basic medium. Different derivatives were produced by adding different electron-donating and withdrawing groups to the different positions of the phenyl B ring of the parent chalcone. The ability of nine morpholine-based compounds 5–13 to inhibit MAO-A and MAO-B was examined in this work. At 1.0 µM, the majority of the drugs inhibited MAO-B by 50%. Most of the nine synthesized compounds showed a strong MAO-B inhibition. The inhibition of MAO-B by the unsubstituted compound 5 was more powerful than that of the other eight derivatives. MAO-B selectivity was shown by all nine derivatives. The potency of compound 5 (IC_50_ = 0.030 ± 0.062 µM) was twice that of the reversible MAO-B inhibitor Lazabemide (IC_50_ = 0.063 ± 0.015 µM) has a high selectivity index (SI = >1333.3), revealing the most significant inhibitor in the series.^7^

**Scheme 5:**
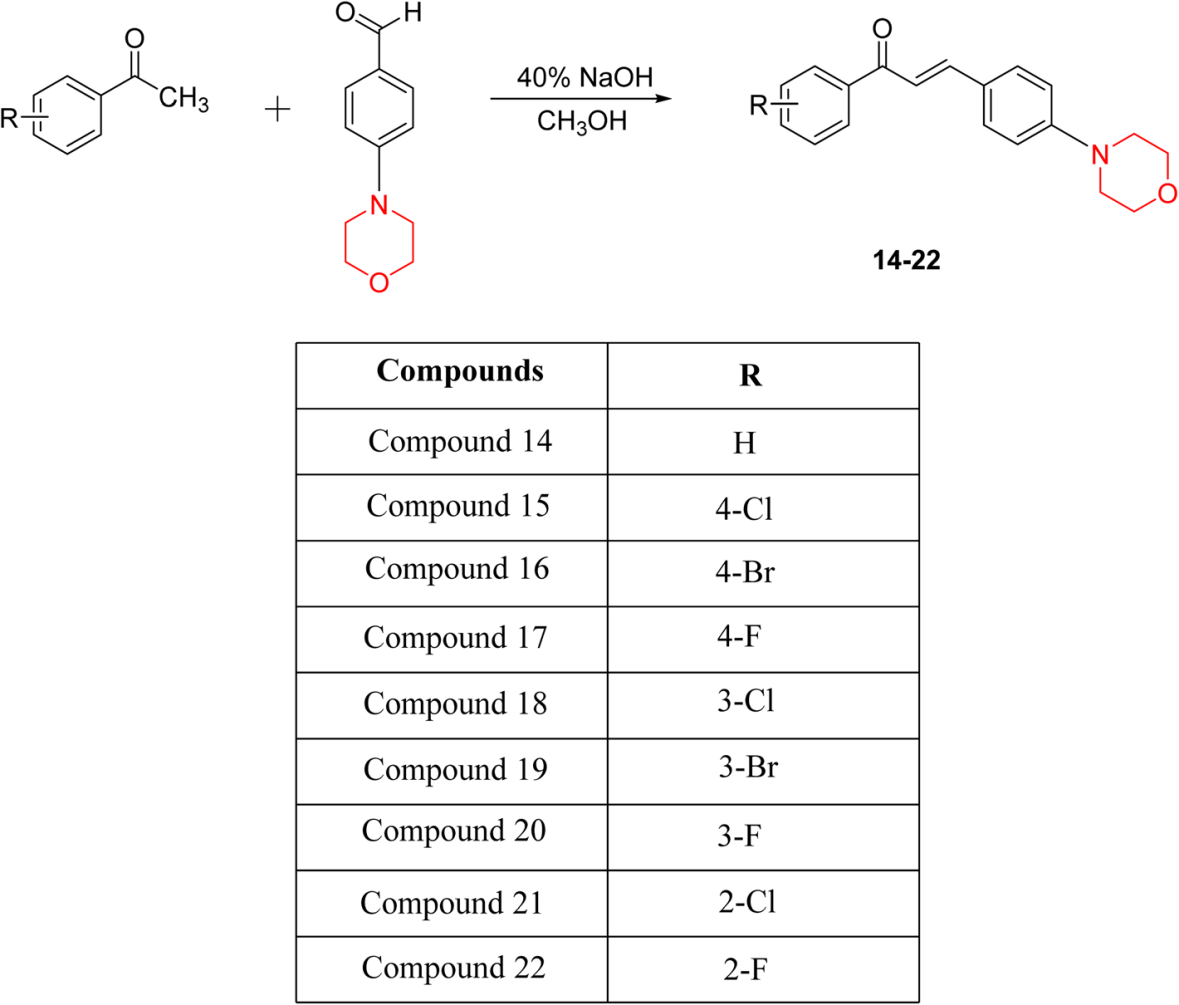
Synthesis of morpholine-substituted α,β-unsaturated ketones 14–22.

Another similar study by Lee *et al.* focuses on substituting different halogens on the phenyl A ring of the chalcones and evaluates the MAO inhibitory impact of the morpholine heterocyclic system on the *para* position of ring B of the phenyl system (Scheme 5). The compounds examined have less residual activity for MAO-B at 1 µM and MAO-A at 10 µM. Among the series of nine compounds (14–22), compound 18 had the highest inhibitory potential against MAO-B with an IC_50_ value of 0.065 ± 0.014 µM, trailed by compound-20 (IC_50_ = 0.078 ± 0.007 µM) and compound-19 (IC_50_ = 0.082 ± 0.012 µM). The compound 15 exhibited an IC_50_ of 0.82 ± 0.25 µM, showing the highest MAO-A inhibiting effect. In comparison to MAO-A, all of the compounds exhibited more inhibitory action against MAO-B, with high SI values for MAO-B. When this series of compounds was compared to reference compounds for the inhibition of MAO-B, compound 18 was found to be less potent when compared to the reference compound, a reversible MAO-B inhibitor, safinamide. However, it was just as potent as or even more so than pargyline. Kinetics and reversibility studies pointed out that compounds 17 and 18 were competitive and reversible inhibitors. Molecular docking of lead compounds 17 and 18 was carried out using 2V5Z as PDB ID and found out important pi–pi interaction between the morpholine-attached phenyl group and Tyr326. The docking scores of compound 17 and compound 18 were −10.64 ± 0.14 and −10.92 ± 0.08, respectively. Further molecular dynamics studies revealed the stability of these compounds with MAO-B and ADMET studies pointing out their favorable properties. In this study it was observed that the biological activity of morpholine–chalcone derivatives strongly depends on the type and position of halogen substitution. When a chlorine atom is placed at the third position of the aromatic ring, the compound shows a clear preference for MAO-B inhibition. This may be explained by the ability of chlorine to fit well within the hydrophobic pocket of MAO-B, thereby stabilizing the interaction. On the other hand, introducing a fluorine atom at the fourth position shifts the activity towards MAO-A inhibition. The smaller size and strong electronegativity of fluorine likely promote favorable interactions in the MAO-A active site. These findings indicate that even subtle changes in substitution pattern can switch selectivity between MAO isoforms, underlining the importance of structural modification during drug design. Overall, these morpholine-containing chalcones can be effective in the treatment of neurological disorders.^5^

**Scheme 6:**
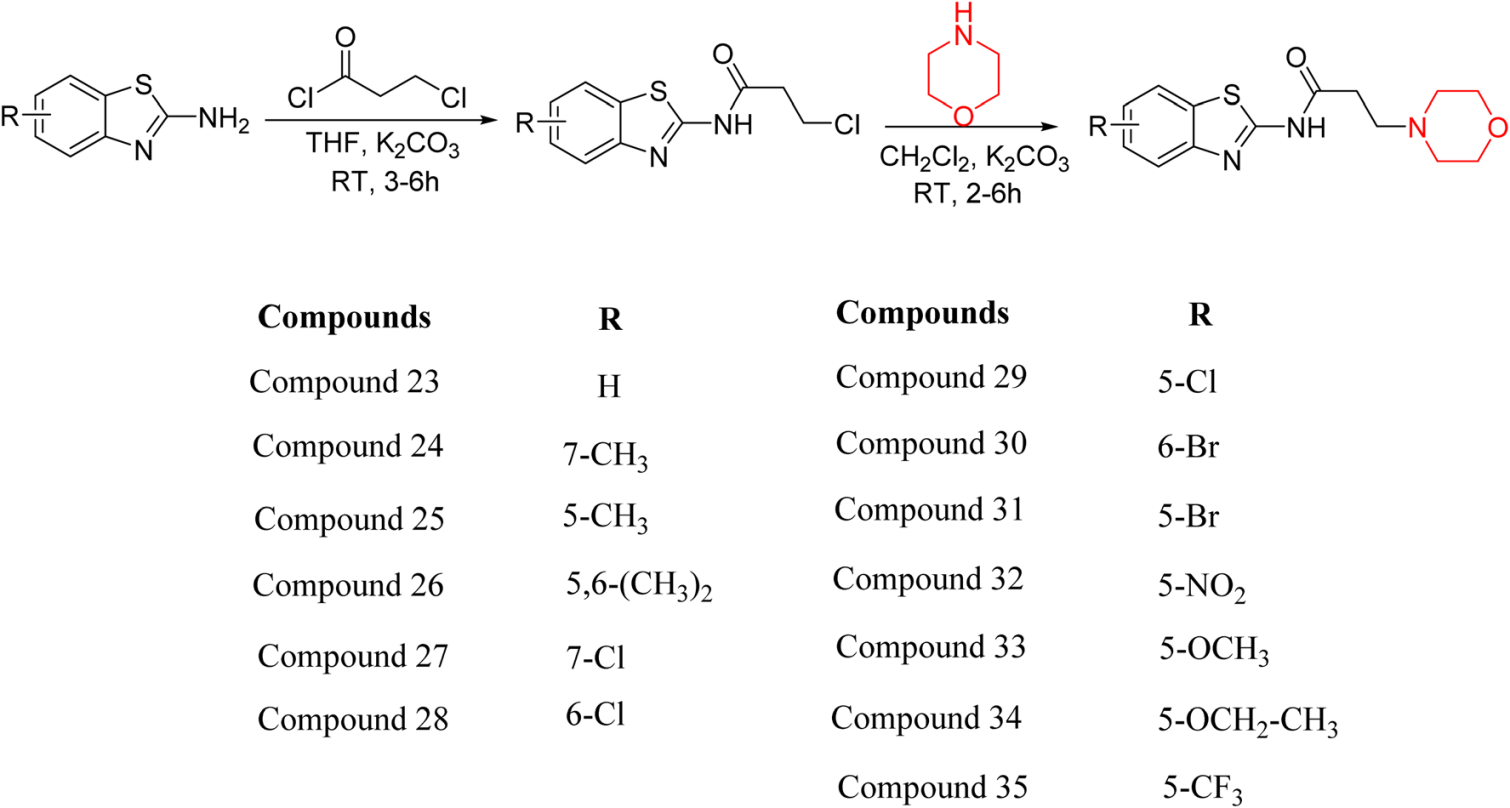
Synthesis of (benzo[*d*]thiazol-2-yl)-3-(morpholino-1-yl)propanamide derivatives 23–35.

Z. He *et al.* developed a series of (benzo[*d*]thiazol-2-yl)-3-(morpholino-1-yl)propanamide derivatives 23–35 by linking 2-amino substituted benzothiazole moiety and morpholine ring using a hydrogen bond donor or acceptor as a linker (Scheme 6). They assessed their MAO inhibitory activity and found that most of the synthesized compounds showed moderate MAO-A inhibition with an inhibitory rate ranging from 1.22% to 24%. The MAO-B inhibitory rate ranges from 8.32% to 20%. These novel compounds may have a better role in treating neurodegenerative disease.^18^

Molecular docking studies reported for morpholine-based MAO inhibitors have consistently shown binding to the substrate cavity in the vicinity of the flavin adenine dinucleotide (FAD) cofactor. The morpholine-based chalcone derivatives were found to preferentially bind to the MAO-B active site, where they participated in π–π stacking interactions with the aromatic residues Tyr326, Tyr398, and Tyr435, which are established to control MAO-B selectivity. Hydrophobic interactions with residues Leu171, Ile198, and Phe343 also contributed to the binding of the morpholine-based chalcone derivatives to the MAO-B active site. Molecular dynamics simulations (100 ns) carried out on selected lead compounds showed the formation of stable protein–ligand complexes, as indicated by low and consistent RMSD values and the absence of RMSF fluctuations at active-site residues. The sustained nature of π–π and hydrophobic interactions during the course of the simulation confirmed the conformational stability of morpholine-based inhibitors in the MAO-B binding pocket. In agreement with the binding interactions, morpholine-based MAO inhibitors were found to possess inhibitory potency in the low micromolar to nanomolar range, with some derivatives showing high selectivity for MAO-B. Besides their strong enzymatic inhibition, some morpholine derivatives of MAO inhibitors have been assessed for their pharmacokinetic properties as potential candidates for CNS drugs. The ADME properties of the lead compounds were predicted to have beneficial gastrointestinal absorption, acceptable volume of distribution, high permeability to the CNS (log *BB* > 0.3), and favorable clearance properties by the *in silico* ADME prediction tools SwissADME and pkCSM, without hepatotoxicity. The results indicate that morpholine derivatives of MAO inhibitors have pharmacokinetic properties that are amenable to CNS drug development.^5,7,17,18^

### 2.2 Inhibitors of cholinesterase

Alzheimer's disease (AD) is the most prevalent neurodegenerative condition currently affecting more than 50 million individuals, with projections estimating an increase to 152 million cases by 2050. AD is marked by memory deterioration, cognitive decline, and impaired reasoning skills, and there is no cure at present. Although the precise cause of AD remains uncertain however it is believed to involve the buildup of β-amyloid plaques, hyperphosphorylated tau neurofibrillary tangles, and a reduction in cholinergic neurotransmission. A deficiency in cholinergic neurons, including acetylcholine and butyrylcholine, is directly associated with Alzheimer's disease. Enzymes like acetylcholinesterase (AChE) and butyrylcholinesterase (BChE) break down acetylcholine into acetate and choline, leading to a reduction in cholinergic neurotransmission. Therefore, inhibiting AChE and BChE has emerged as a promising therapeutic approach to address AD. Currently approved AChE inhibitors such as donepezil, tacrine, rivastigmine, and galantamine improve neurotransmission but come with serious side effects, including nausea, vomiting, weight loss, liver toxicity, and reduced appetite, prompting ongoing research to find safer and more effective ChE inhibitors. Nitrogen-containing heterocyclic compounds are critical pharmacophores found in both natural and synthetic sources, significantly contributing to bioactive pharmaceutical development. Morpholine, among these, is widely used in pharmaceuticals and appears in many FDA-approved and experimental drugs due to its versatile applications.^19–23^

R. Sasidharan *et al.* developed a series of morpholine-containing α,β-unsaturated ketones (Scheme 4, 5–13) by condensing several aromatic *para*-substituted aldehydes and 4-morpholine acetophenone in the presence of an alcoholic basic medium. The substitutions in the B ring of the chalcone are changed with different electron-donating and withdrawing groups. The ability of nine morpholine-based compounds (5–13) to inhibit AChE and BChE was examined in this work. The presence of a *para*-dimethylamino group (compound 9) significantly enhanced AChE inhibition (IC_50_ = 6.1 µM), making it the most active compound of the series. This strong activity can be attributed to the electron-donating resonance effect of the dimethylamino group, which increases electron density on the aromatic ring and favors interactions within the AChE active site. But this *para*-dimethylamino group is less potent for BChE (IC_50_ = 18.09 µM). Bromine-substituted (compound 13) and chlorine-substituted (compound 12) morpholine derivatives also showed AChE inhibition, though with lower potency compared to the dimethylamino derivative. The moderate activity of halogen-substituted compounds may arise from their size and hydrophobic interactions within the enzyme pocket. Further kinetics and reversibility studies revealed that compound 9 and compound 13 were reversible and competitive inhibitors of AChE with a *K*_i_ value of 2.52 µM and 7.04 respectively. Additionally, cytotoxicity studies showed that these lead compounds are nontoxic to normal vero cells and have the potential to lower reactive oxygen species levels. Further docking studies add to the ability of these morpholine-containing lead compounds to inhibit AChE and so can be a promising candidate in the treatment of neurodegenerative disease.^7^

**Scheme 7:**
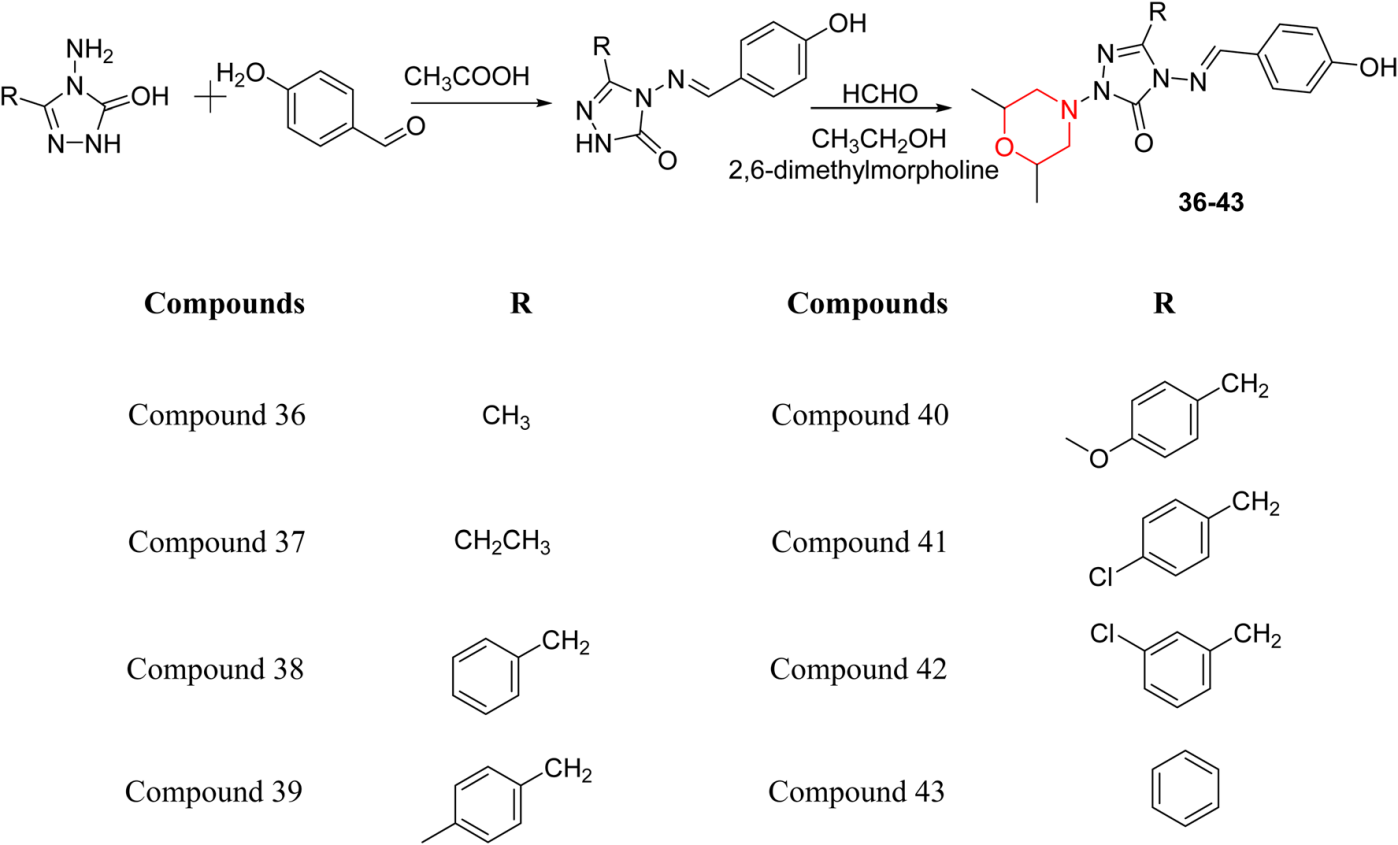
Synthesis of morpholine-substituted compounds 36–43.

S. Boy *et al.* synthesized a series of eight novel compounds, specifically 1-(2,6-dimethylmorpholino-4-yl-methyl)-3-substituted-4-(4-hydroxybenzylidenamino)-4,5-dihydro-1*H*-1,2,4-triazol-5-ones (36–43), by reacting 3-substituted-4-(4-hydroxybenzylidenamino)-4,5-dihydro-1*H*-1,2,4-triazol-5-ones with 2,6-dimethyl morpholine in the presence of formaldehyde (Scheme 7). They highlighted how the nature of the substituent at the 3rd position of the triazolone ring critically influences the inhibitory activity of morpholine-containing derivatives toward cholinesterases. Among these synthesized derivatives, compound 39, bearing a benzyl substituent, showed the most potent inhibition of BChE with an IC_50_ value of 34.97 µM. The enhanced BChE inhibition is likely due to the favorable fit of the benzyl group into the hydrophobic pocket of the enzyme, allowing stable π–π interactions and additional hydrogen bonding through the morpholine and hydroxybenzylidene oxygen atoms. Compound 40, which has a methoxybenzyl group, was the most effective at stopping AChE, with an IC_50_ value of 35.36 µM. Docking studies revealed that these lead compounds exhibit significant inhibitory effects on both AChE and BChE receptors. The AChE active site includes acyl and choline-binding regions as well as a peripheral site connected to the active site by a gorge. Inhibitors acting at the peripheral site and gorge are preferred due to their potential to influence the catalytic centre. Compound 40 interacts with AChE through amino acid residues within these sites, while compound 39 forms both direct and indirect hydrogen bonds with the Gln119, Tyr332, and Gly117 residues of the BChE enzyme. The oxygen atoms of the dimethylmorpholine and hydroxybenzylidene groups act as hydrogen acceptors in these interactions. Therefore, these novel compounds hold potential as treatments for Alzheimer's disease.^23^

**Scheme 8:**
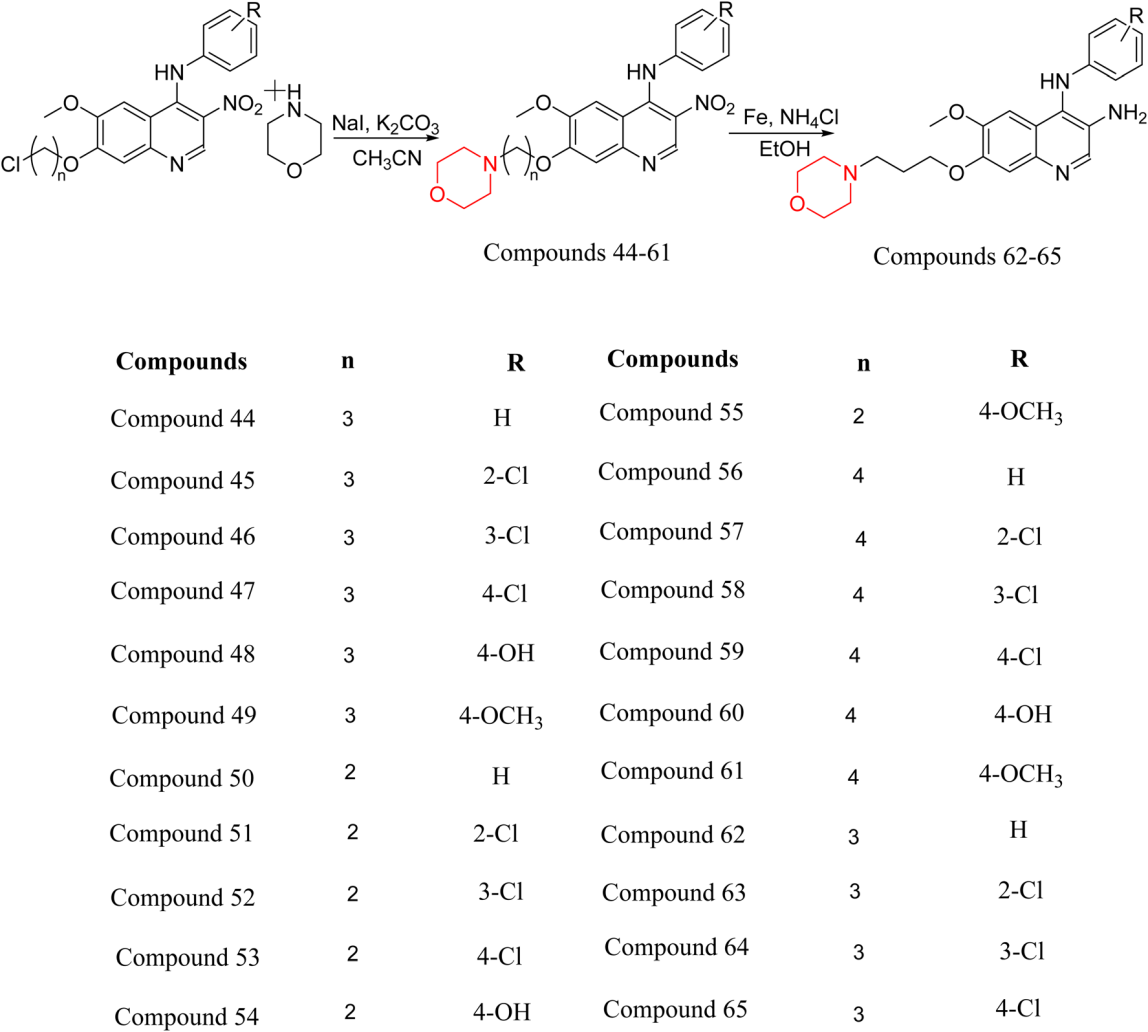
Synthesis of morpholine-substituted compounds 44–65.

Liu *et al.* synthesized a new series of 4-*N*-phenylamino quinoline derivatives with a morpholine group (44–65) (Scheme 8) and evaluated their anti-cholinesterase activity and ABTS radical scavenging potential for Alzheimer's disease (AD) treatment. Among these, compound 50 demonstrated strong AChE inhibition with an IC_50_ value of 1.94 ± 0.13 µM and moderate BChE inhibition at 28.37 ± 1.85 µM in comparison to the standard reference galantamine. The highest BChE inhibition was shown by compound 64 with an IC_50_ of 27.84 ± 2.16 µM. Most compounds had a selectivity index (SI) above 1, which indicates a stronger AChE inhibition than BChE inhibition. Compounds with a two-methylene linker between quinoline and morpholine (44–61) showed superior AChE inhibition compared to those with three- or four-methylene linkers; a similar trend was observed for BChE inhibition. Kinetic studies revealed that potent compounds 44 and 50 act as mixed-type AChE inhibitors. Docking studies showed that compound 44 binds to the CAS site of AChE, whereas its nitro group interacts with Tyr341 and Try337, and it also interacts with the PAS site through four amino acid interactions, including two carbon-hydrogen bonds formed between its morpholine moiety and Ser293. Compound 62 formed three carbon-hydrogen bonds between its morpholine moiety and Ser293. Overall, both compounds (44 and 62) showed similar amino acid interactions and binding orientations with slight variations in binding patterns. Additionally, compound 50 showed interactions with key amino acid residues in the PAS, AS, and OAH active sites of BChE, which suggests its potential for further investigation. Notably, compounds 44, 50, and 62 demonstrated the ability to cross the blood–brain barrier (BBB) and exhibited suitable solubility and lipophilicity for brain penetration.^20^

**Scheme 9:**
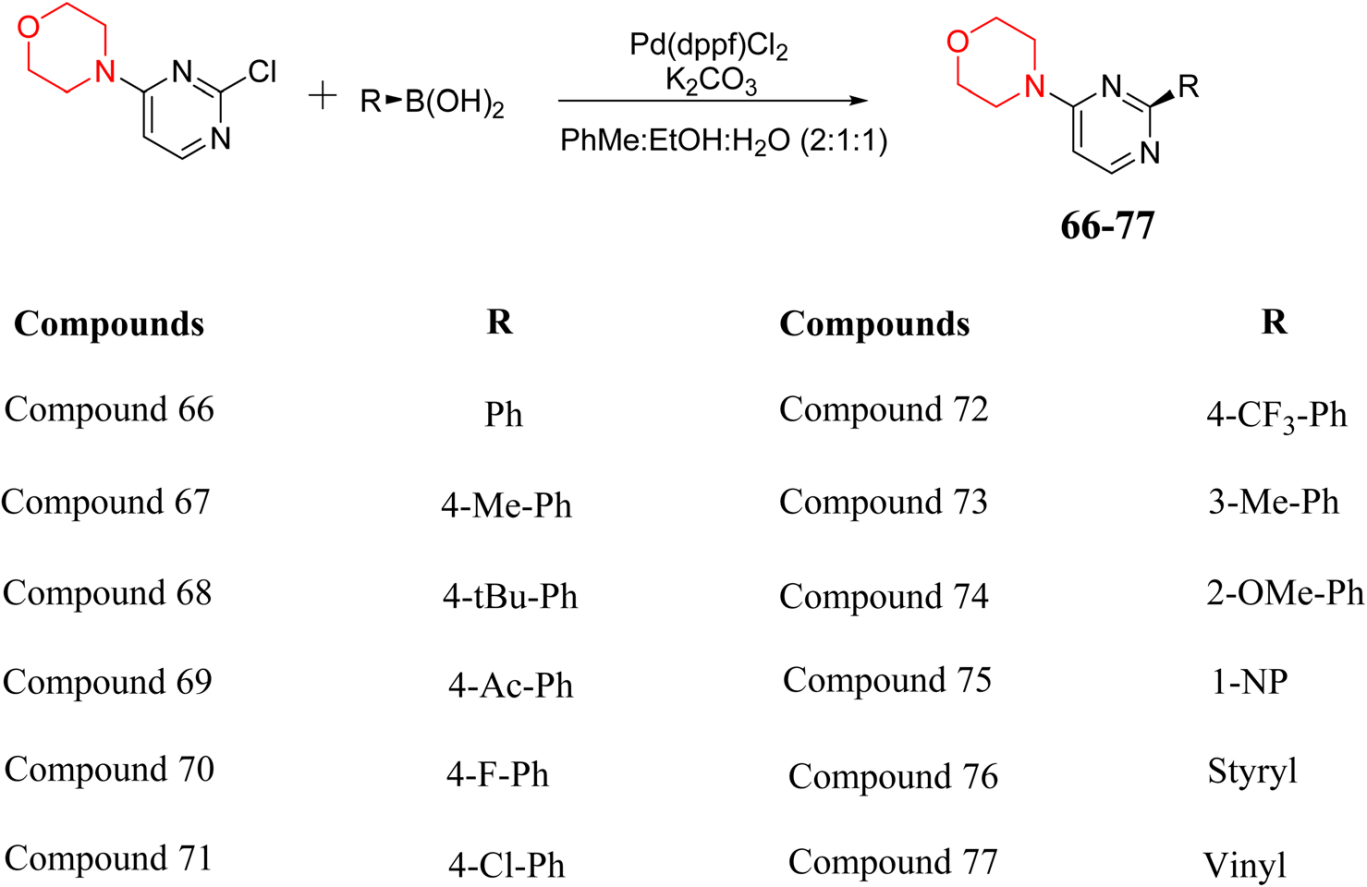
Synthesis of pyrimidine–morpholine hybrids (66–77).

S. Zaib and colleagues synthesized a series of pyrimidine–morpholine hybrids (66–77, shown in Scheme 9) as potent cholinesterase inhibitors with potential as anti-Alzheimer's agents. The twelve compounds were evaluated *in vitro* for their ability to inhibit AChE and BChE enzymes. Among them, compound 73, which has a *meta*-tolyl substitution, was the most promising and demonstrated strong AChE inhibition with an IC_50_ of 0.43 ± 0.02 µM, which is approximately 38 times more potent than the standard drug neostigmine (IC_50_ = 16.2 ± 1.01 µM). Additionally, compound 73 efficiently inhibited BChE, with an IC_50_ of 2.5 ± 1.12 µM, which was approximately three times more potent than donepezil (IC_50_ = 7.19 ± 1.14 µM). The strong activity of compound 73 may be attributed to the favorable steric orientation and electronic effects of the *meta*-methyl group, which enhance hydrophobic and π–π interactions within both enzyme active sites. The AChE was selectively inhibited by compound 74 (4-fluorophenyl) with an IC_50_ value of 8.9 ± 0.62 Μm, indicating that *ortho*-substitution may introduce steric hindrance, reducing optimal binding. However, *para*-substituents such as acetyl (compound 69) and fluoro (compound 70) had IC_50_ values of 1.4 ± 0.11 µM and 0.78 ± 0.11 µM, respectively. According to kinetic studies, compound 73 inhibits AChE in a non-competitive manner. The results of docking studies with lead compounds 69, 70, and 73 showed that compound 73 functions as a dual inhibitor for both AChE and BChE, whereas compounds 69 and 70 exclusively inhibit AChE. Docking of compound 69 into the AChE catalytic site revealed a hydrogen bond between ASP74 and the oxygen atom of the morpholine group (3.05 Å). In the active pocket between TYR124 and the morpholine ring, a π-alkyl interaction and a carbon–hydrogen bond have been noticed. Compound 70 displayed interactions within AChE's active site with key amino acids, where morpholine formed multiple interactions, including carbon-hydrogen bonds with SER125 and TYR133, and a π-alkyl interaction with TRP86. The most powerful compound 73 also interacted with important residues in AChE's active site, such as TRP86, TRP439, TYR337, TYR449, and PRO446. Docking of compound 73 in the BChE active site revealed multiple binding interactions, including π–π T-shaped, alkyl, π-alkyl, hydrogen bonds, and carbon-hydrogen bonds. Molecular dynamics simulations of the dual inhibitor compound 73 confirmed the stability of its protein–ligand complex with minimal deviations for both AChE and BChE. Additionally, ADME studies supported the potential of pyrimidine–morpholine hybrids as promising candidates for Alzheimer's disease treatment.^21^

**Scheme 10:**
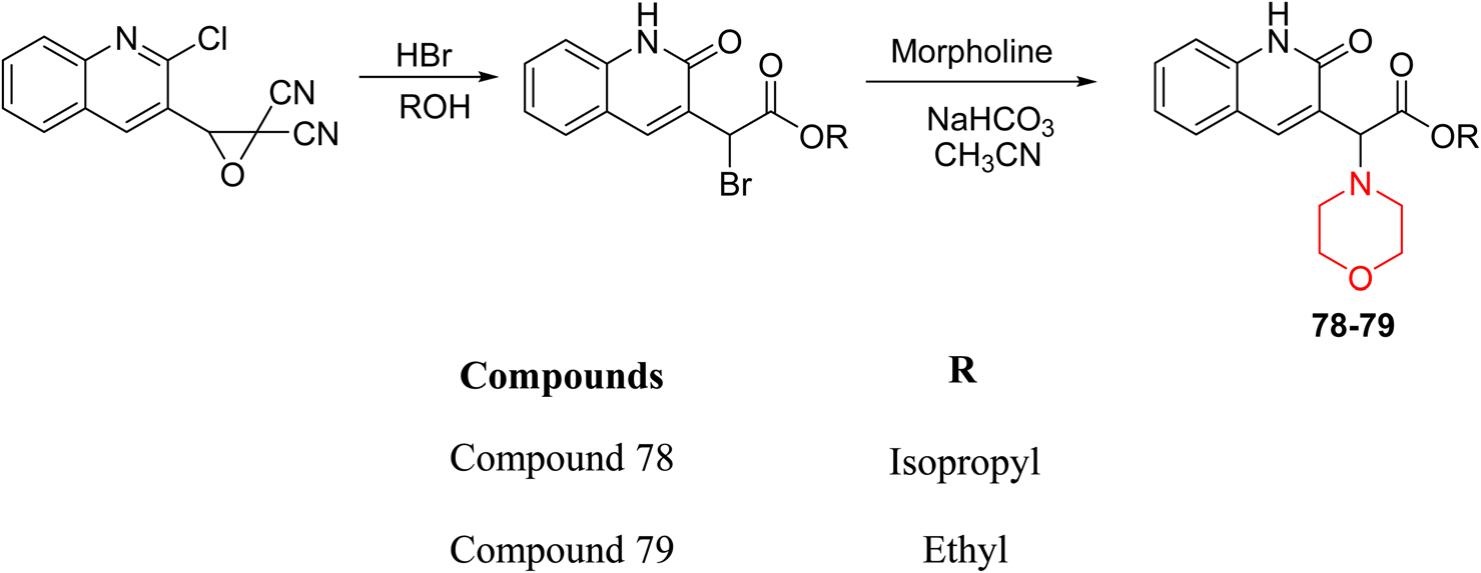
Synthesis of 2-quinolone-morpholines (78–79).

H. Alliouche *et al.* synthesized and characterized 2-quinolone-morpholine derivatives 78 and 79 (Scheme 10). They employed density functional theory (DFT) for molecular structure optimization, analyzed frontier molecular orbitals and molecular descriptors, and conducted NMR, optical, and electronic spectral analysis studies. When compared to the reference drug tacrine, docking studies showed that these compounds have a stronger affinity for butyrylcholinesterase (BChE) and acetylcholinesterase (AChE) as well as strong interactions with important amino acids. Towards AChE, both compounds obtained the same docking scores of −10.1, whereas for BChE, 78 and 79 had scores of −9.4 and −9.1, respectively. These compounds exhibit a strong affinity for AChE and BChE, making them interesting candidates for treating Alzheimer's disease.^24^

**Scheme 11:**
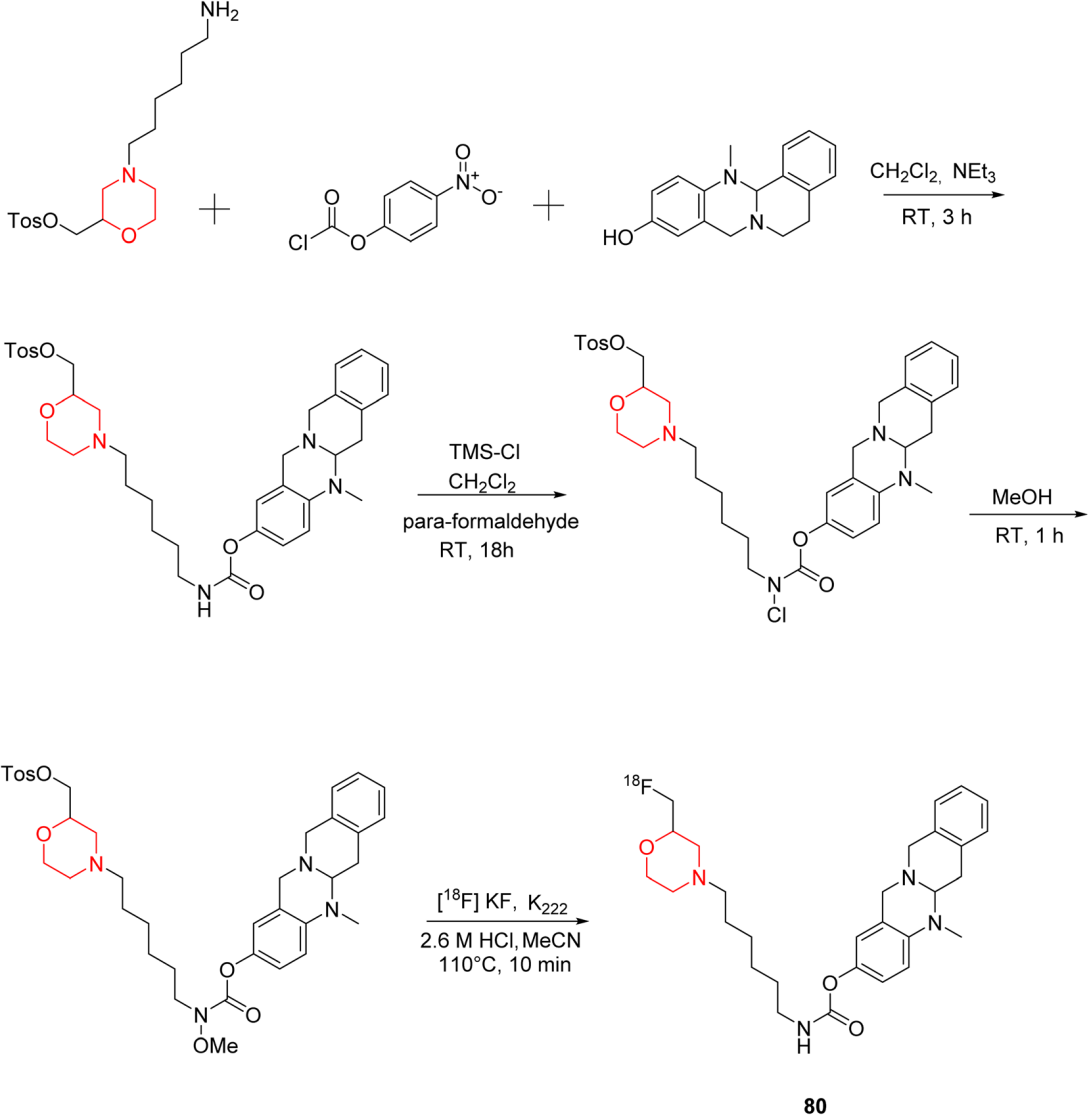
Synthesis of morpholine-substituted compound 80.

C. Gentzsch *et al.* designed and synthesized an ^18^F-labeled radiotracer 80 (Scheme 11) based on a carbamate-derived BChE inhibitor scaffold for PET imaging of BChE. Targeting the BChE enzyme with imaging probes holds promise for the early diagnosis of Alzheimer's disease. The hexylene linker between the carbamate and morpholine units is optimized to maintain both inhibitory strength and extended action duration, with the morpholine group specifically affecting the inhibition period. *In vitro* tests showed that compound 80's inhibitory potency (IC_50_ = 66.6 nM) closely matches that of the parent compound (IC_50_ = 49.3 nM). Additionally, *ex-vivo* autoradiography in mouse brains demonstrated strong brain tissue binding of this compound.^19^

**Fig. 3:**
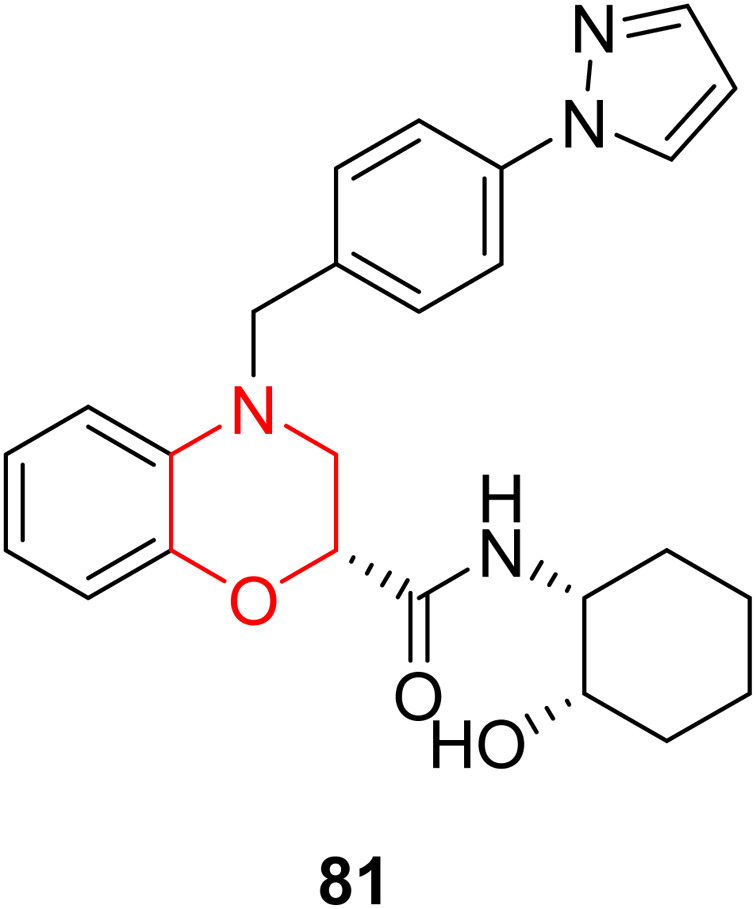
Structure of compound 81.

K. S. Abd-Elrahman *et al.* focused on 81 (Fig. 3), an AChE inhibitor developed earlier to evaluate its effects on neuroglial activation in both APP/PS1 and wild-type mice. Their research revealed that compound 81 significantly reduces the presence of Iba1-positive microglia and GFAP-positive astrocytes in the hippocampus of these mice while also limiting the recruitment of these cells to amyloid β plaques. With its dual effects of AChE inhibition and decreased neuroglial activation, compound 81 shows promise as a potential treatment for Alzheimer's disease, particularly in female subjects.^22^

Z. He *et al.* linked a 2-amino-substituted benzothiazole moiety and a morpholine ring using a hydrogen bond donor or acceptor as a linker and developed a series of (benzo[*d*]thiazol-2-yl)-3-(morpholino-1-yl)propanamide derivatives (23–35 in Scheme 6). The synthesised compounds were assessed for their inhibitory effects on AChE and BuChE enzymes. Most of them exhibited moderate AChE inhibition with inhibitory rates varying between 1.99% and 32.04% except for compound 26, which showed no activity against AChE. Among the tested derivatives, compound 30 demonstrated the highest inhibitory activity against AChE, reaching 32.04%. However, none of the compounds exhibited BuChE inhibition. This research highlights the potential of morpholine-based compounds as neuroprotective agents.^18^

Docking studies of morpholine-containing cholinesterase inhibitors demonstrate that these compounds bind within the deep active-site gorge of acetylcholinesterase (AChE) and butyrylcholinesterase (BChE), encompassing both the catalytic active site and the peripheral anionic site. The morpholine moiety facilitates hydrogen bonding interactions *via* its heteroatoms, whilst aromatic substituents participate in π–π stacking interactions with essential residues, including Trp84, Trp286, Phe295, and Tyr337, which are recognised for their significant role in cholinesterase inhibition. For quinoline-morpholine hybrids, molecular docking studies revealed favourable binding poses and binding energies with RMSD values of less than 1 Å, confirming the accuracy of the binding poses predicted. While extensive molecular dynamics simulations have not been extensively documented for such systems, the conservation of primary hydrogen bonding and π–π interactions in docking studies indicates the structural integrity of morpholine-based ligands in the binding pocket of cholinesterase and explains their inhibitory activity. In agreement with these binding features, the reported morpholine-based cholinesterase inhibitors exhibited inhibitory activity spanning low micromolar to sub-micromolar ranges against AChE and BChE.^7,20,21,23,24^

### 2.3 Norepinephrine reuptake inhibitors

Anxiety is a mental disease characterized by dread, tiredness, tension, excessive restlessness, and irritation.^25,26^ The exact etiology of anxiety disorder is unknown, although research suggests that it might be caused by oxidative stress. An increase in reactive oxygen species reduces the synthesis of dopamine, norepinephrine, and serotonin. Serotonin reuptake inhibitors or norepinephrine reuptake inhibitors are medications used to treat anxiety.^26^

**Scheme 12:**
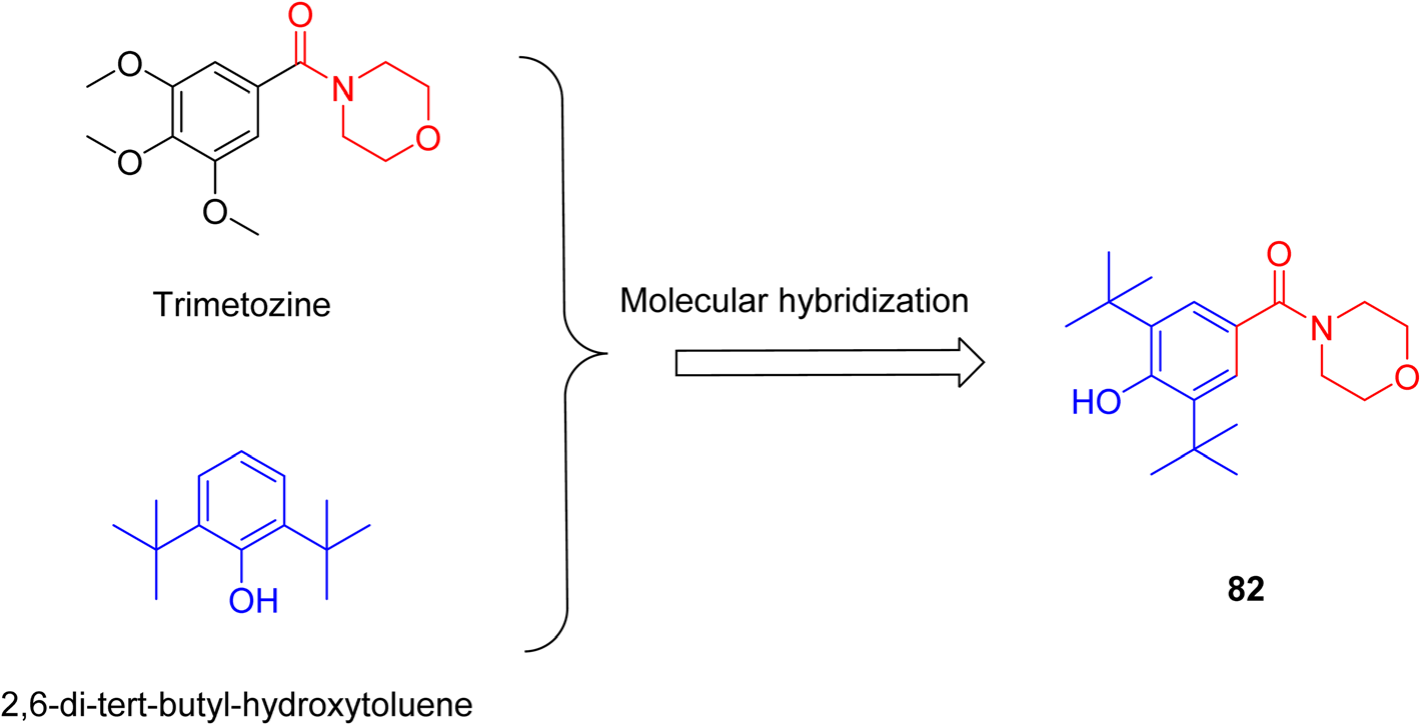
Design stratergy of compound 82.

Cabral *et al.* synthesised a novel compound 82, providing insight into pharmacological characteristics. The compound 82 was developed by molecular hybridization of the morpholine-containing anti-anxiety drug trimetozine with the antioxidant 2,6-di-*tert*-butyl-hydroxytoluene (Scheme 12). Docking experiments revealed substantial interactions with benzodiazepine binding sites while ADMET assays demonstrated the blood–brain barrier permeability. Mice have been assessed for motor performance using methods such as the wire test, rotarod test, and chimney test. The findings indicate that compound 82 at a high dosage of 20 mg kg^−1^ might produce a sedative effect or impair motor performance. However, at a dosage of 10 mg kg^−1^, the drug had potential anxiolytic effects that did not interfere with motor coordination. Pretreatment with flumazenil reduced the anxiolytic action of compound 82, indicating that it participates in the benzodiazepine binding site.^25^

To ascertain the potential therapeutic use of compound 82, J. Pereira *et al.* employed electrochemical and Density Functional Theory (DFT) techniques. This compound possessed two peaks in the redox mechanism, while its electrochemical behaviour was examined using voltammetry; one peak was caused by the oxidation of the BHT phenolic fraction, while the second peak was related to the oxidation of the amino group of the morpholine ring. Electrochemical analysis and the conjoint perspective of DFT offer substantial insights into a compound's oxidative behaviour.^26^ According to these two investigations, compound 82 has antioxidant and norepinephrine reuptake-inhibiting properties, making it a potentially effective therapy option for anxiety disorders.

**Fig. 4:**
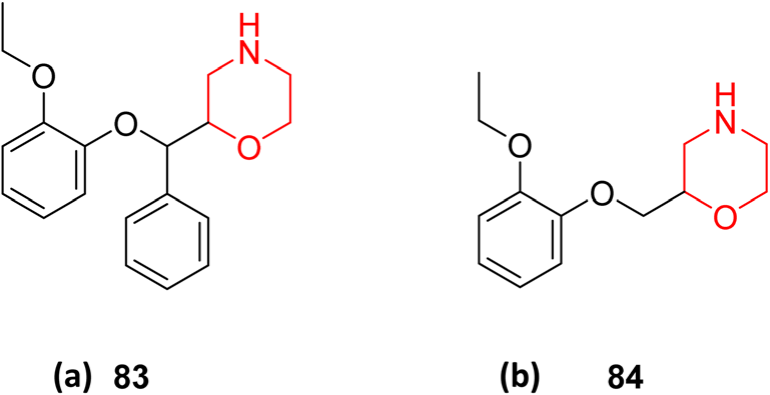
(a) Structure of reboxetine (83); (b) structure of viloxazine (84).

Obstructive Sleep Apnea (OSA) is a common sleep-related breathing disorder. Frequent arousals during sleep and recurrent narrowing and collapse of the pharyngeal airway are the primary features of this disorder. Untreated OSA can result in cardiovascular and neuropsychological disorders. In healthy people, several studies have demonstrated that the antimuscarinic medication hyoscine butyl bromide and the noradrenergic drug reboxetine enhance upper airway function during sleep.^27^ Reboxetine (83 in Fig. 4) is a selective norepinephrine reuptake inhibitor having a morpholine ring that is used as an antidepressant and anti-anxiety drug. Narcolepsy, obstructive sleep apnoea (OSA), and attention deficit hyperactivity disorder (ADHD) can all be effectively treated with it.^9,10,27,28^ According to a *meta*-analysis of randomized controlled trials (RCTs), reboxetine is safe and effective in treating negative symptoms of schizophrenia. It was also shown to be tolerable when used as an adjuvant medication to antipsychotic therapy.^10^

R. Lim *et al.* examined how 15 healthy individuals with OSA responded to reboxetine plus hyoscine butyl bromide. According to this study, reboxetine (4 mg) and hyoscine butyl bromide (20 mg) together lessen the severity of OSA in participants by reducing the apnoea/hypopnoea index (AHI) by more than 15 episodes per hour.^27^ The impact of the noradrenergic drug reboxetine alone and in conjunction with oxybutynin on the severity of OSA was further investigated by TJ Altree *et al.* They discovered that a single dosage of reboxetine improves snoring and nighttime oxygenation in patients with severe OSA while lowering the frequency of respiratory episodes. Furthermore, there is no extra benefit to employing oxybutynin for managing the severity of OSA. This study offers new insight into the noradrenergic mechanism in OSA by revealing that the noradrenergic drug reboxetine alone is effective in treating the condition.^28^

Attention deficit/hyperactivity disorder (ADHD) is a neurobehavioral condition characterized by a pattern of inattentiveness, hyperactivity, and impulsiveness that manifests in various kinds of scenarios, including home and school. ADHD is more common in children and adolescents than in adults. ADHD therapy authorized by the US FDA is categorized as stimulants (*e.g.*, methylphenidate, amphetamine, and lisdexamfetamine) or non-stimulants (*e.g.*, clonidine, atomoxetine, and guanfacine). Stimulants are considered first-line therapy for ADHD due to their better effectiveness and onset of action, but they have certain drawbacks, including the risk of abuse, cardiovascular problems, sleeplessness, weight loss, and hunger. Despite these restrictions, non-stimulants or a combination of stimulants and non-stimulants will be beneficial in ADHD treatment.^13,16^

Viloxazine (84 in Fig. 4) is a morpholine ring containing a non-stimulant that the US FDA authorised in April 2021 for the treatment of ADHD in children and adolescents. It increases norepinephrine levels by inhibiting norepinephrine reuptake as well as serotonin and dopamine levels in the prefrontal cortex, which is significantly linked to ADHD. Viloxazine is well-tolerated in clinical trials with relatively minor adverse effects, such as gastrointestinal problems.^11,13–16^

S. L. Faison *et al.* undertook a study to compare the pharmacokinetics and safety of combination treatment with the non-stimulant viloxazine extended-release (viloxazine ER) and stimulant methylphenidate to viloxazine ER or methylphenidate alone. This is a single-centre randomized research of 36 healthy people who received single doses of viloxazine (700 mg), methylphenidate (36 mg), or a combination of the two. The blood sample was taken over four days following the administration of drugs and analyzed using chromatographic tandem mass spectrometry. By examining their pharmacokinetics and assessing safety, no drug–drug interaction was observed when providing the combination when compared to therapy with either drug alone.^16^

**Fig. 5:**
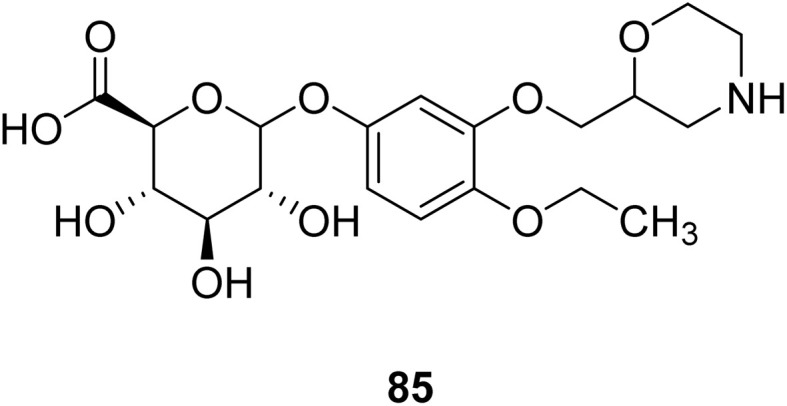
Structure of 5-hydroxyviloxazine glucuronide (85).

Nasser *et al.* investigated the pharmacokinetics of viloxazine ER and its primary metabolite, 5-hydroxyviloxazine glucuronide (5-HVLX-gluc) (compound 6 in Fig. 5), in which both have a morpholine ring in their structure. To assess the safety and effectiveness of viloxazine ER in the treatment of pediatric ADHD patients, data from four phase 3 multicenter, randomized, double-blinded, placebo-controlled, three-arm parallel group studies were analyzed using a population pharmacokinetic model. They also assessed how missing a dosage of viloxazine for one to four days might affect its pharmacokinetics. According to the model, body weight significantly impacts the dosage of viloxazine; children and adolescents who weigh more have lower drug exposure, while those who weigh less have higher drug exposure. Additionally, this model demonstrated that although the drug concentration quickly returns to its steady state, missing a dosage (one to four days) had no discernible impact on the therapeutic and safety profile.^14^ Further, they conducted another study to measure the benefits and risks of viloxazine ER treatment. The Likelihood to be Helped or Harmed (LHH) effect size measures the overall benefit-risk ratio that must be considered when selecting a treatment, and the Number Needed to Treat (NNT) measures the benefit of treatment, whereas the Number Needed to Harm (NNH) measures the risk of discontinuing treatment. On evaluation, the LHH value for viloxazine ER varies from 5 to 13, implying that the patient will benefit 5 to 13 times more than the risk from viloxazine medication. The NNT value was less than ten, while NNH values more than or equal to ten indicate that the viloxazine ER therapy is safe and effective.^13^ Psychometric tools are used to assess patients' symptoms and functional impairments during clinical trials of psychiatric medications. In a clinical trial of viloxazine extended release, Nasser *et al.* used the ADHD Rating Scale-5 (ADHD-RS-5) to measure the severity of symptoms and the Weiss Functional Impairment Rating Scale-Parent (WFIRS-P) to measure functional impairment. They correlated these scales with Clinical Global Impressions (CGI) scales, which evaluate functional impairment in terms of clinical relevance. They discovered that a “much improved” rating on the Clinical Global Impressions Improvement (CGI-I) scale is linked to around a 55% improvement on the ADHD-RS-5 and a 40% improvement on the WFIRS-P scale. These findings will help medical professionals and scientists better understand how therapy affects people with ADHD.^12^

### 2.4 Restoration of dopamine content

Drug addiction leads significantly to mortality, major health problems, and societal challenges in many nations. Addiction is caused by central nervous system stimulants that raise dopamine levels in the brain above the physiological level. The central and peripheral nerve systems include G-protein-coupled dopamine receptors. Dopamine receptors possess five different subtypes: D1, D2, D3, D4 and D5. Among them, D3 plays a crucial part in drug addiction.

**Scheme 13:**
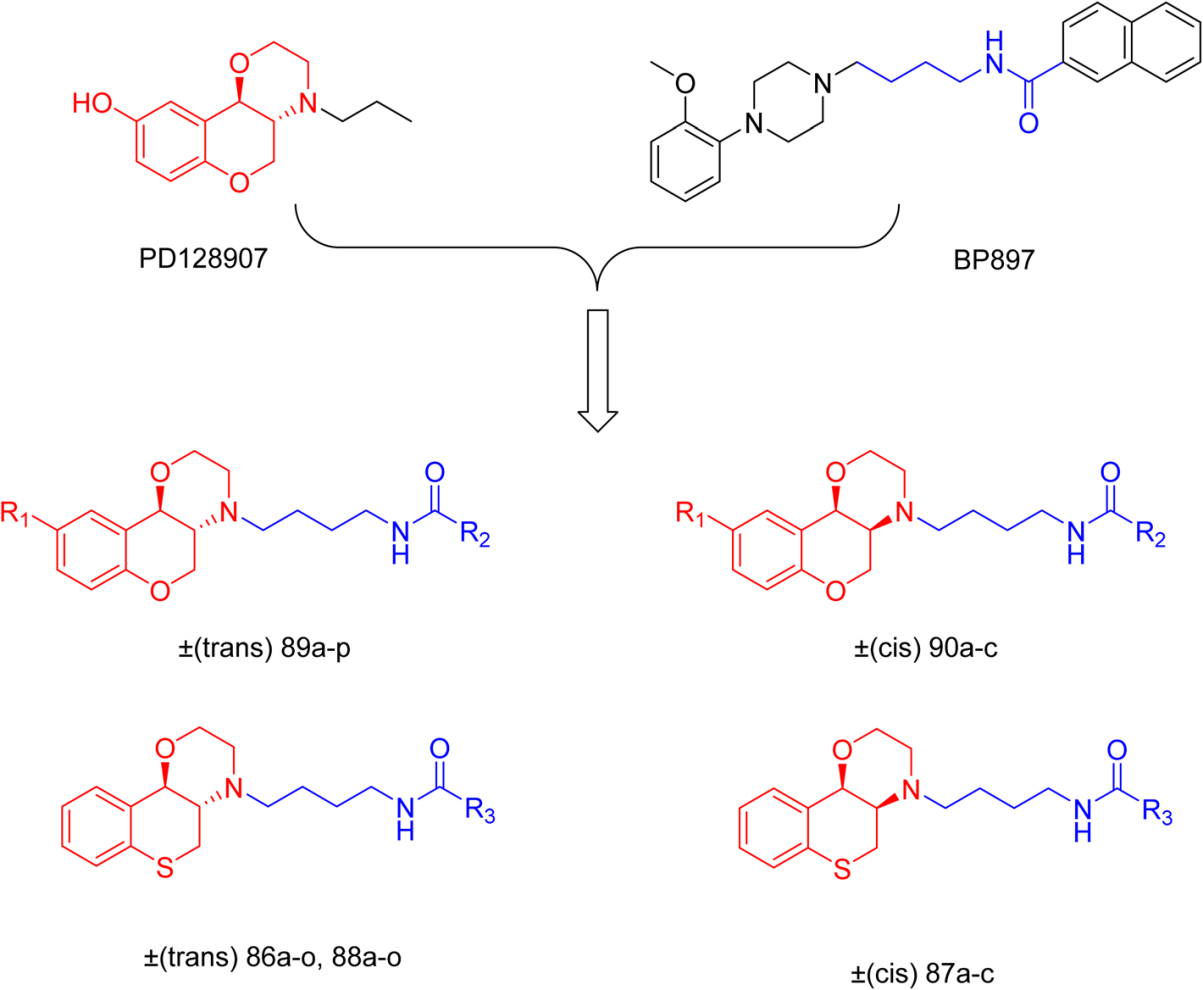
Design strategy for selective dopamine receptor agonists.

**Scheme 14:**
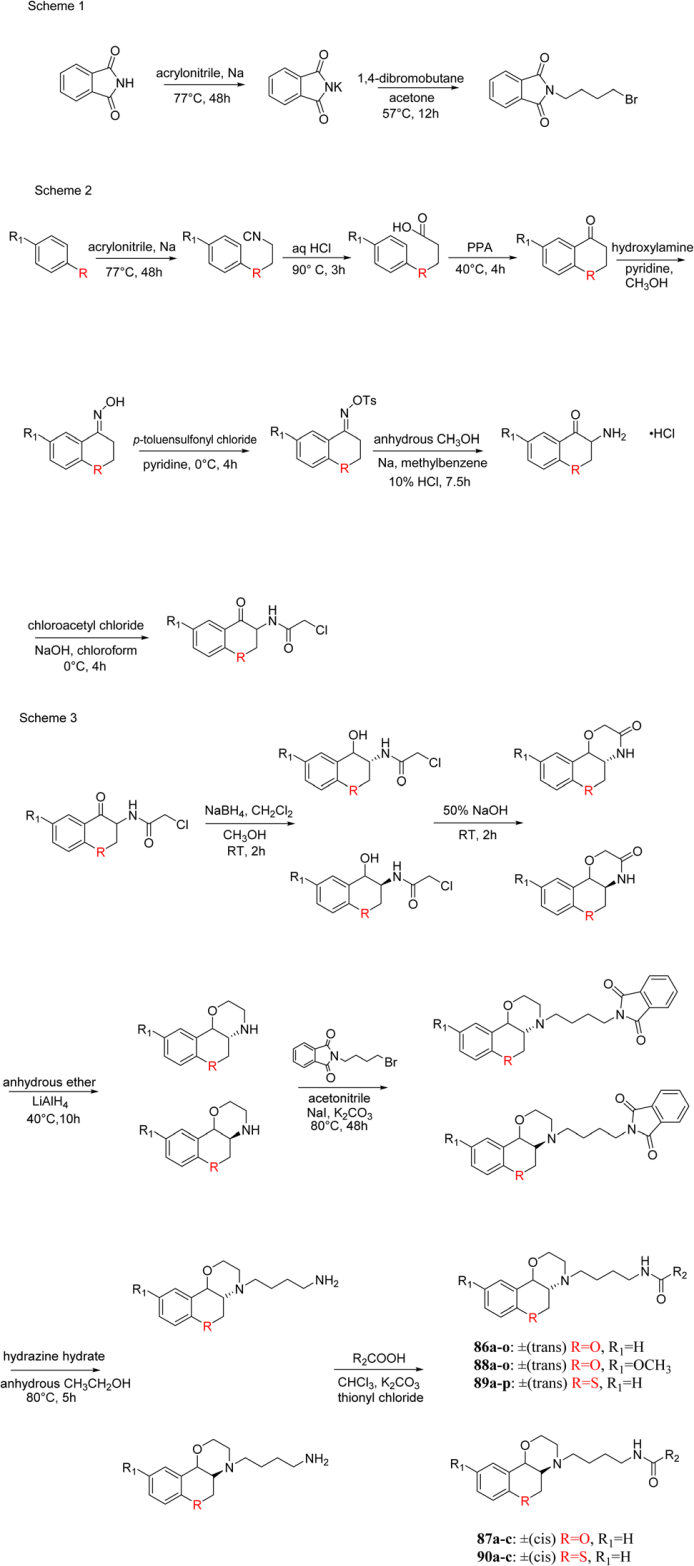
Synthesis of benzopyranomorpholine and benzothiophenemorpholine derivatives 86–90.

J. Cai *et al.* designed and synthesized a series of 33 bitopic benzopyranomorpholine analogues (86a–o, 87a–c, and 88a–o) and 19 benzothiophene morpholine analogues (89a–p and 90a–c) (Scheme 14) and assessed for their anti-drug addiction potential. These analogues were designed from potent D3 receptor partial agonists PD128907 and BP897 (Scheme 13). All of the synthesized compounds are tested for human D3 and D2 binding assays utilising PD128907 and BP897 as reference materials. Results are converted to equilibrium dissociation constants (*K*_i_ values) based on the IC_50_ value. The D3 receptor exhibited greater binding affinity and selectivity than the D2 receptor for the majority of the synthesized compounds. In the case of benzopyranomorpholine analogue, compounds 88a–o were discovered to be excellent binders, among which compound 88h demonstrated superior binding affinity compared to reference compounds with a *K*_i_ value of 1.08 ± 0.16 nm, while in the benzothiophene morpholine series, compound 89b exhibited maximum binding affinity with *K*_i_ value of 1.32 ± 0.16 nM. Stereochemistry has a significant impact on affinity since in both series it was found that the compounds with a *trans*-morpholine ring have a higher affinity for D3 than compounds with a *cis*-morpholine ring. Animal behavioural models of naloxone-induced withdrawal symptoms in morphine-dependent mice were used to evaluate the capacity of the compounds to mitigate the symptoms of opioid addiction. The results demonstrated that in the benzopyranomorpholine series, compound 88h outperformed the reference drug (sulpiride) in minimizing withdrawal symptoms in morphine-dependent rats, while in the case of the benzothiophenemorpholine series, compounds 89a and 89d were the best-performing ones. Additionally, the lead compounds 88h, 89a, and 89d were docked into the binding pockets of D3R and D2R. It was discovered that it is well occupied in the D3 receptor's binding pockets and that it exhibits significant interactions with critical amino acids, including Ser409, Glu90, Ser366, Tyr373, and Asp110. So these novel series of drugs can be effective in treating drug addiction.^29,30^

These two studies on benzopyranomorpholine and benzothiophene morpholine derivatives lead us to the conclusion that molecules with morpholine rings play an important role in anti-addiction treatment. When it comes to drug addiction treatment, benzothiophene morpholine derivatives work more effectively than benzopyranomorpholine derivatives.

Morpholine derivatives as dopamine receptor ligands, especially D3 subtype, have been studied in detail by structure-based methods. Docking calculations using the crystal structure of the human D3 receptor showed that bitopic benzopyranomorpholine derivatives bind to both the orthosteric binding site and a secondary extracellular pocket, which is essential for D3 selectivity over D2 receptors. The protonated morpholine nitrogen atom usually formed electrostatic interactions with conserved acidic residues like Asp110, while heteroaromatic or aromatic moieties were involved in hydrogen bonding and π–π stacking interactions with residues like Glu90, Ser366, Tyr373, Phe345, and His349. These stabilizing interactions are in line with the high binding affinities measured experimentally, with several derivatives showing nanomolar D3 receptor affinity (*K*_i_ ≈ 0.9–10 nM) and high D3/D2 selectivity ratios. Although detailed molecular dynamics simulations were not provided uniformly, the interaction profiles inferred from docking calculations suggest stable receptor–ligand complexes that explain the observed *in vitro* binding and *in vivo* anti-addiction activity.^29,30^

### 2.5 Inhibitors mTOR kinase

The Mechanistic Target of Rapamycin (mTOR) is a protein kinase from the phosphatidylinositol-3-kinase-related kinase family, which forms two distinct multiprotein complexes called mTOR complex 1 (mTORC1) and mTOR complex 2 (mTORC2). These complexes differ mainly due to the presence of a protein named Raptor in mTORC1 and Rictor in mTORC2. mTORC1 regulates cellular energy, stress responses, amino acids, oxygen levels, and growth factors, while mTORC2 governs cytoskeletal organization, cell cycle progression, and survival. Dysregulation of the mTOR pathway is implicated in cancer, type 2 diabetes, and neurodegenerative disorders such as Parkinson's, Alzheimer's, Huntington's disease, epilepsy, and stroke. First-generation mTOR inhibitors, such as rapamycin and its analogs, function as allosteric inhibitors of mTORC1; however, they initiate upstream signaling that constrains their clinical effectiveness. Moreover, these inhibitors, known as rapalogs, have poor blood–brain barrier (BBB) penetration, presenting a significant challenge in finding mTOR inhibitors with optimal BBB permeability. Developing selective TORKi is also difficult due to structural similarities between the catalytic sites of mTOR and phosphoinositide-3-kinase (PI3K).^31,32^

**Scheme 15:**
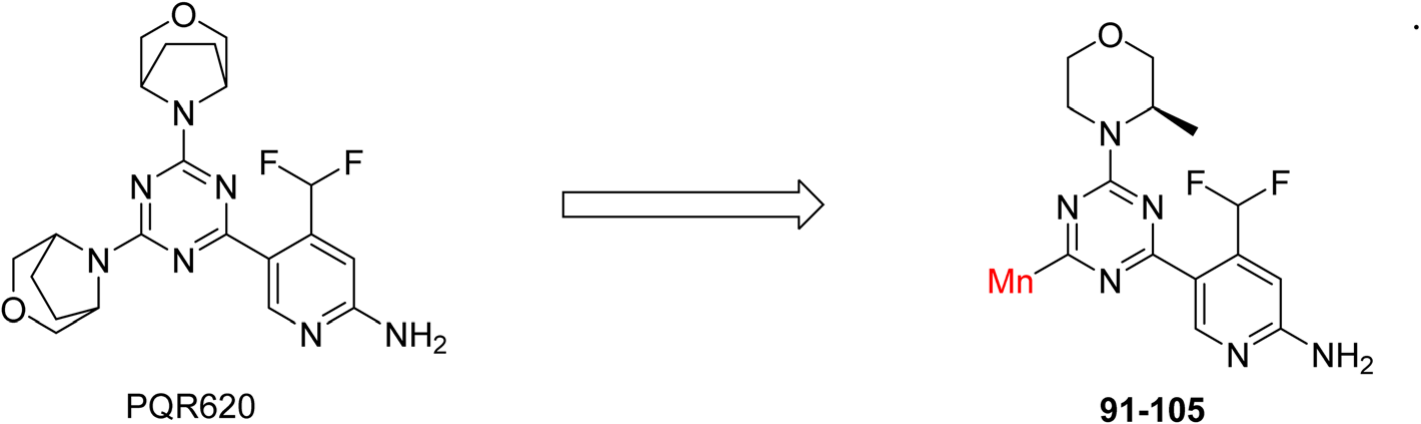
Design of morpholine-substituted derivatives.

**Scheme 16:**
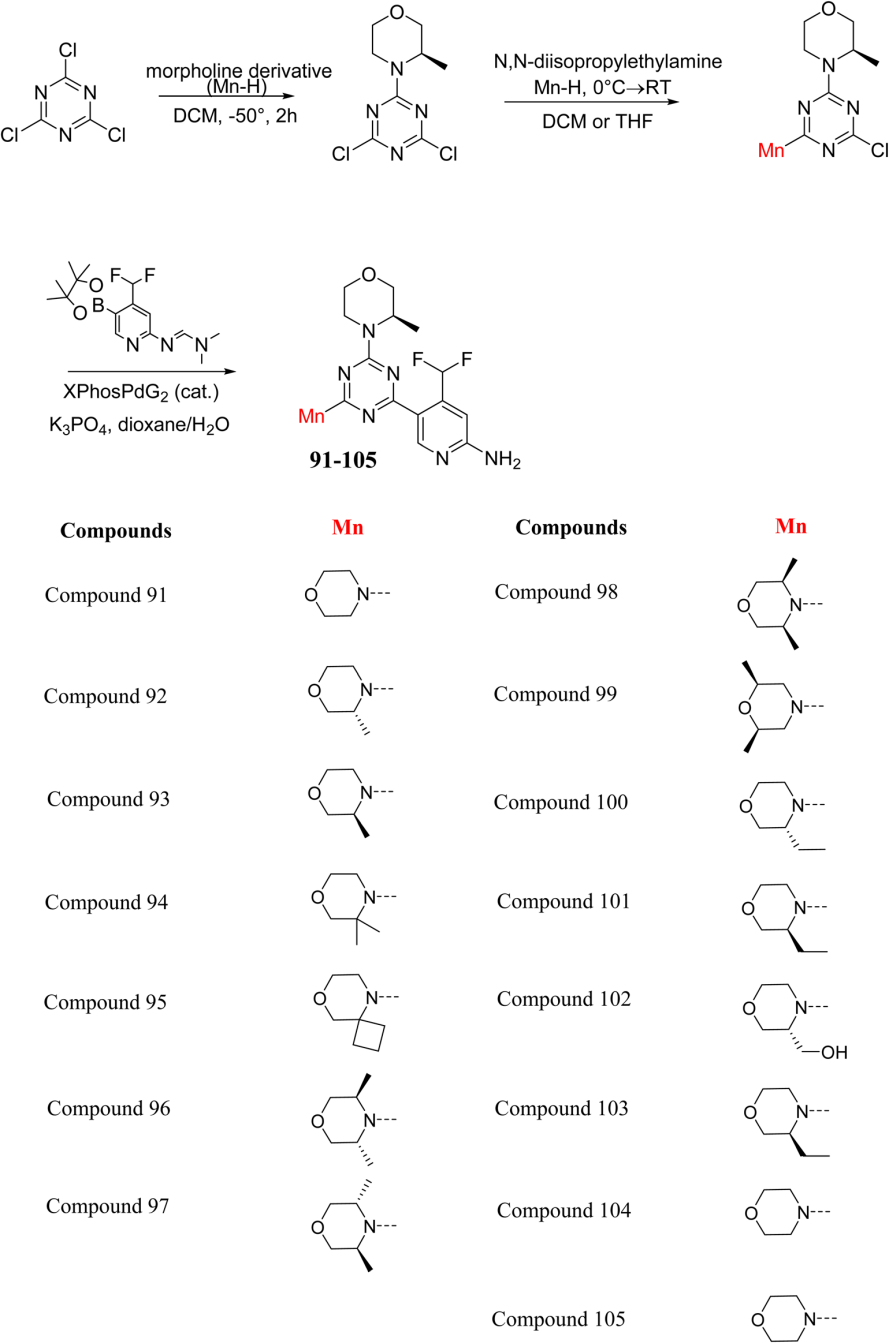
Synthesis of morpholine-substituted derivatives 91–105.

C Borsari *et al.* previously discovered the compound PQR620 as an mTOR inhibitor, but they found it to have less metabolic stability in humans, so they further developed a series of compounds (91–105, Scheme 16) by modifying the morpholine ring of the compound PQR620 (Scheme 15). In their previous study, they found that compound PQR620, having two morpholine rings in its structure, undergoes rapid metabolism in humans by oxidation of the bridged morpholine. At the same time, triazine and 4-(difluoromethyl)pyridine-2-amine did not show any metabolic reaction. So modifying the morpholine ring can overcome this problem and lead to the development of highly stable mTOR inhibitors. All the synthesized compounds were tested for *in vitro* binding to the catalytic subunit of mTOR and PI3Kα and for inhibition of the PI3K/mTOR pathway in A2058 cells. Among these compounds, 94 with a *K*_i_ value of 4.7 ± 0.38 nM and compound 97 with a *K*_i_ value of 3.6 ± 0.98 nM were the most potent compounds. The morpholine moiety contributes to the selectivity of compounds towards mTOR rather than PI3K. Further computational study of compounds 94 and 97 was performed, and their interaction with the ATP-binding site of the respective kinase was investigated by using the X-ray structure of mTOR complexed with PI103 (PDB ID 4JT6). They found out that the mTOR-compound 97 complex has shown key interactions, including a hydrogen bond interaction between the oxygen atom of the substituted morpholine ring and Val2240 backbone. Compared to compound 94, compound 97 demonstrated greater stability towards CYP1A1 metabolism. Additionally, research revealed that compound 97 does not interact with non-targets. Compound 97 was assessed for its metabolic stability in hepatocytes of mice, rats, dogs, and humans and compared with that of compound PQR620. They found that compound 97 outperformed compound PQR620 and exhibited intermediate metabolic stability in mice, rats, dogs, and humans. blood–brain permeability assay *in vivo* model showed that compound 97 can cross the BBB and it has a greater concentration in the brain than plasma. Additionally, compound 97 has no effect on P-gp-mediated efflux, recommending that it can be used to treat seizures associated with tuberous sclerosis complex (TSC).^32^ Overall, the SAR suggests that morpholine substitution is essential for mTOR selectivity over PI3K. In particular, hydrogen bonding from morpholine oxygen (as in compound 97) is critical for high affinity and stability. Moreover, modifications on the morpholine ring not only reduce oxidative metabolic liability but also maintain blood–brain barrier permeability without triggering P-gp efflux. Together, these findings demonstrate that careful morpholine optimization preserves the inhibitory activity while improving metabolic resilience and pharmacokinetic balance, with compound 97 emerging as the most promising representative of the series.

Structure-based research has already shown that morpholine is an important hinge-binding substructure of ATP-competitive mTOR inhibitors. Crystallographic and docking analyses show that the morpholine oxygen and nitrogen atoms are involved in important hydrogen-bond interactions with the backbone of Val2240 in the mTOR hinge region, while properly substituted heteroaromatic cores are extended into the affinity pocket to interact with residues such as Lys2187, Asp2195, Tyr2225, and Asp2357. Sterically hindered or bridged morpholine analogues selectively bind to the deeper mTOR hinge pocket, thus increasing selectivity against closely related PI3K isoforms. Several morpholine-containing scaffolds display sub-micromolar to low-nanomolar mTOR inhibitory potency (IC_50_ ≈ 0.007–0.15 µM), which is consistent with stable binding modes as observed in structure-based studies. While molecular dynamics simulation data are not consistently presented for all series, available crystallographic and docking data offer strong support for stable and selective morpholine-mTOR interactions, thus explaining the observed biochemical potency and CNS relevance.^31,32^

### 2.6 β-secretase inhibitors

The β-secretase (BACE1) enzyme has a significant role in the treatment of Alzheimer's disease since it is responsible for the amyloid β plaque deposition, which is included in the pathogenesis of Alzheimer's disease. The enzyme cleaves the extracellular domain of Amyloid Precursor Protein (APP) into an N-terminal fragment (C99), which is further processed by γ-secretase into neurotoxic peptides like Aβ40 and Aβ42, contributing to the disease. Developing BACE1 inhibitors with effective blood–brain barrier penetration remains a significant challenge.

**Scheme 17:**
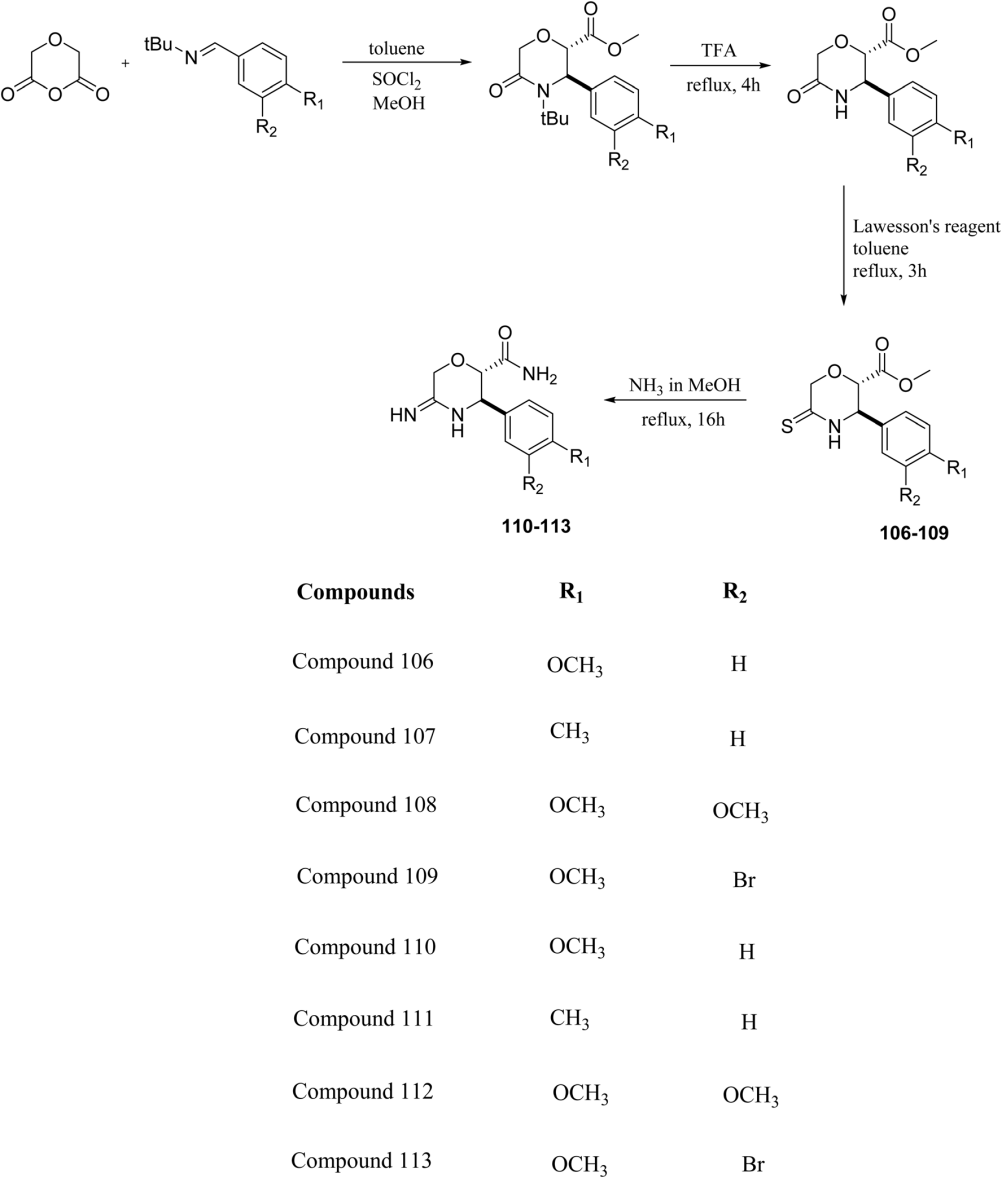
Synthesis of morpholine-substituted derivatives 106–113.

L. Calugi *et al.* developed novel C-2 substituted morpholine derivatives (Scheme 17) containing a thioamide (106–109) or an amidino group (110–113) and evaluated them for their drug-likeness and BACE1 inhibitory activity. Drug-likeness was assessed by CNS Multiparameter Optimization (CNS MPO), which scores six physicochemical properties: partition coefficient, molecular weight, hydrogen bond donors, topological polar surface area, distribution coefficient at physiological pH, and pKa. All synthesized morpholine-thioamide compounds 106–109 showed superior CNS MPO of greater than 4, whereas in the case of amidino (110–113), only compound 111 satisfies the physiological profile of a CNS drug. In BACE1 inhibition assays, compound 106 demonstrated the highest potency, achieving 40% inhibition at 10 µM. The presence of sulfur in thioamide compounds, which act as hydrogen bond acceptors, contributed significantly to activity, as thioamides outperformed amidino derivatives. Further, they evaluated the most potent compound 106 for molecular docking studies and revealed significant hydrogen bonding interactions, and the phenyl substituent in the morpholine ring established a π stacking interaction, which enhanced its binding affinity.^33^

Morpholine-derived BACE-1 inhibitors primarily function through direct binding to the catalytic aspartate dyad (Asp32 and Asp228) in the active site of the enzyme. In C-2 substituted morpholine analogs, especially those incorporating cyclic thioamide or amidino moieties, micromolar levels of BACE-1 inhibition were observed, with IC_50_ values in the range of 5–6 µM for representative compounds. Molecular docking studies showed that the thioamide moiety is essential for hydrogen bonding interactions with the catalytic aspartates, with the morpholine oxygen atom contributing to binding affinity in the S1/S3 pockets. Further π–π stacking interactions between aromatic substituents and residues in the flap domain (*e.g.*, Tyr71) also improve binding affinity. While comprehensive molecular dynamics studies were not generally presented, docking-based conformational analyses clearly indicate that the morpholine core is an appropriate non-covalent template for BACE-1 inhibition with desirable CNS drug-like properties.^33,34^

### 2.7 Sigma-1-receptor activation

The Sigma-1 receptor (Sigma-1 R) is an intracellular chaperone protein widely distributed across various organs, particularly in the brain's nigrostriatal pathway. It plays a key role in reducing oxidative stress and supports essential chaperone functions, including protein folding, translocation, and degradation. Additionally, it helps in regulating lipid and cholesterol balance by interacting with the insulin-induced gene 1 protein. Sigma-1 R is a promising therapeutic target for Parkinson's disease due to its involvement in addressing protein misfolding and lipid-related dysfunctions implicated in the disease progression. Furthermore, it aids in managing stress and anxiety by modulating dopamine receptors (D1 and D2) and regulating anxiolytic proteins.^35,36^

**Fig. 6:**
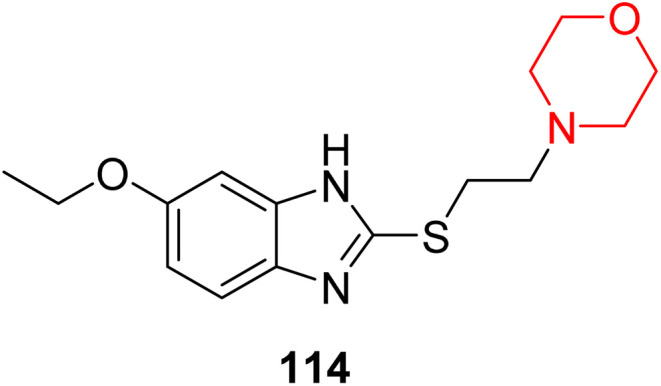
Structure of afobazole (114).

I. Kadnikov *et al.* investigated the anxiolytic drug afobazole, chemically known as 5-ethoxy-2-[2-(morpholino)-ethylthio]benzimidazole dihydrochloride (Fig. 6, 114), for its potential to restore dopamine levels *via* the Sigma-1 receptor. Using a 6-hydroxydopamine (6-OHDA)-induced mouse model, they administered afobazole, PRE-084 (a selective Sigma-1R agonist), BD-1047 (a selective Sigma-1R antagonist), and combinations of afobazole with either PRE-084 or BD-1047 and evaluated the effects. Afobazole demonstrated notable Sigma-1R affinity with a *K*_i_ value of 5.9 µM. The study revealed that afobazole helped restore dopamine levels in the 6-OHDA-induced model and improved motor performance in rotarod tests. These findings highlight the role of the Sigma-1 receptor in dopamine restoration and suggest that Sigma-1 agonists like afobazole could serve as promising therapeutic candidates for Parkinson's disease.^35^

M. Voronin *et al.* explored the involvement of the Sigma-1 receptor (Sigma1R) in the anxiolytic effects of fabomotizole (ethoxy-2-[2-(morpholino)-ethylthio]benzimidazole dihydrochloride) (114 in Fig. 6) by using a combination of *in vivo* behavioural experiments and *in silico* docking studies. Behavioural results showed that fabomotizole produced anxiolytic-like effects in BALB/c mice during elevated plus maze (EPM) tests, which were completely inhibited by Sigma1R antagonists BD-1047 and NE-100. Docking studies using Sigma1R structure bound to (+)-pentazocine (PDB ID: 6DK1) revealed that fabomotizole interacts with the Sigma1R binding site similar to the known agonists and forms key interactions such as hydrogen bonds and salt bridges with amino acid residues like Glu172 and Phe107. The morpholine group of fabomotizole exhibited rotational flexibility, enabling interactions with the 6DK1 binding site akin to those of pentazocine. This study highlights Sigma1R's role in the anxiolytic activity of fabomotizole, offering new insights into Sigma1R-mediated mechanisms of anxiolysis and emphasizing the multitarget pharmacological profile of fabomotizole, which broadens its potential in anxiety disorder treatment.^36^

### 2.8 Monoacylglycerol agonist

**Fig. 7:**
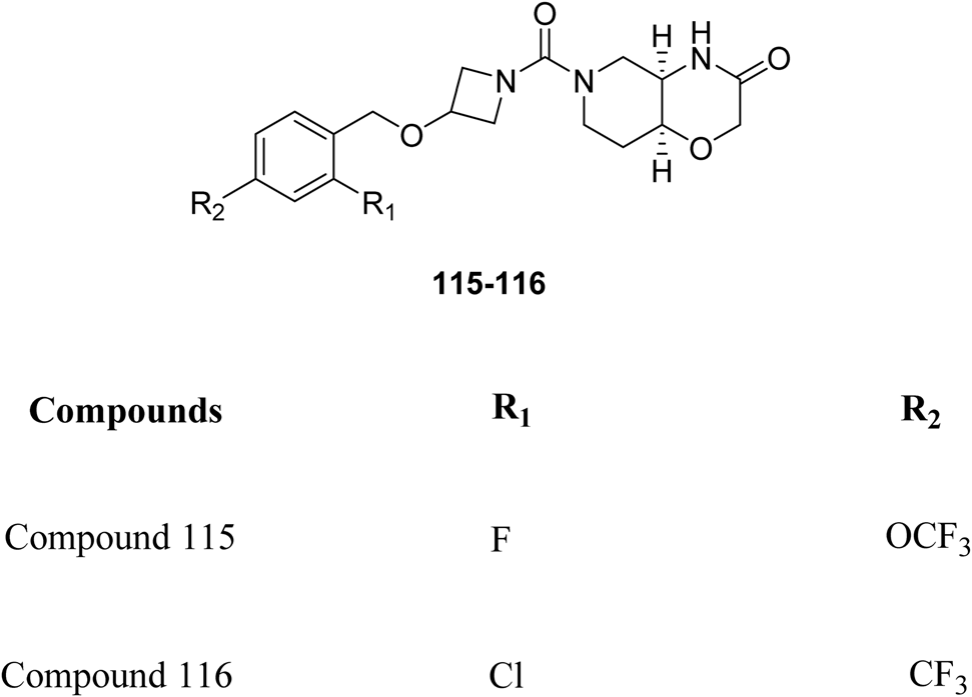
Structure of morpholine-3-one derivatives, 115–116.

Novel morpholine-3-one derivatives were investigated by Y. He *et al.* as reversible PET radioligands for imaging monoacylglycerol lipase (MAGL), which is a crucial target in neurodegenerative and neuroinflammatory disorders. Two candidates (115 and 116, Fig. 7) were selected from the synthesized compounds due to their high affinity and appropriate physicochemical characteristics for neuroimaging. Both showed remarkable selectivity and specificity for MAGL in vitro autoradiographic investigations. Using both wild-type (WT) and MAGL knockout (KO) mice in PET experiments, compound 116 demonstrated *in vivo* specificity. Although low brain uptake due to P-glycoprotein efflux posed a challenge, compound 116 emerged as a promising lead for MAGL imaging.^37^

### 2.9 Cannabinoid receptor agonist

**Fig. 8:**
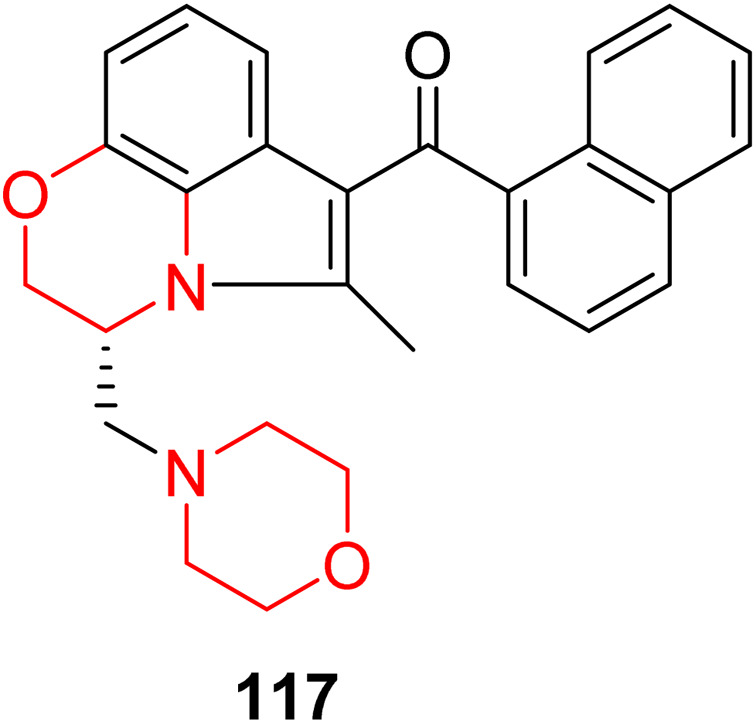
Morpholine-ringed cannabinoid agonist WIN 55,212-2 (117).

P. Valenzuela *et al.* investigated the effects of adolescent exposure to the morpholine-ringed cannabinoid agonist WIN 55,212-2 (117, Fig. 8) on the adult nigrostriatal dopaminergic system. Their findings revealed that WIN 55,212-2 elevated extracellular dopamine levels in the dorsolateral striatum (DLS) and increased the activity of dopamine neuron populations in the substantia nigra pars compacta (SNc) in adulthood. These effects coincided with a significant decrease in the extracellular gamma-aminobutyric acid (GABA) levels in the SNc. Furthermore, the increased dopamine neuron activity brought on by adolescent cannabis exposure was reversed by injecting bicuculline, which is a GABAa receptor antagonist, into the ventral pallidum (VP).^38^

The role of ghrelin/GHS-R1A in the rewarding and reinforcing actions of the cannabinoid CB1 agonist WIN 55,212-2 (117), which includes a morpholine moiety, was investigated by C. Charalambous *et al.*^39^ Their research concentrated on dopamine release, endocannabinoid levels, and GABA modulation in the nucleus accumbens shell (NACSh). They found that WIN 55,212-2 induced a transient decrease in GABA levels, while the levels of the endocannabinoids (anandamide and 2-arachidonoylglycerol) increased and dramatically boosted dopamine release in the NACSh. Notably, these cannabinoid-induced effects were significantly reduced by pretreatment with the GHS-R1A antagonist JMV2959.^39^

Further, D. Gomez *et al.*^40^ looked at the impact of long-term exposure to the synthetic cannabinoid WIN 55,212-2 on the dopamine-releasing properties of heroin and other cannabinoids. They discovered that WIN 55,212-2 triggers both cross-tolerance to heroin's dopamine-releasing effects and tolerance to its own. This was evidenced by a diminished capacity of both WIN and heroin to enhance the frequency and amplitude of dopamine release events in rats with prior exposure to WIN.^40^

Brewer *et al.* examined somatic and anxiety-like behaviours during both induced and spontaneous withdrawal in order to comprehensively assess cannabis withdrawal in male and female rats using WIN 55,212-2. They discovered that both male and female rats developed dependence after receiving long-term doses of the synthetic cannabinoid WIN 55,212-2.^41^

**Fig. 9:**
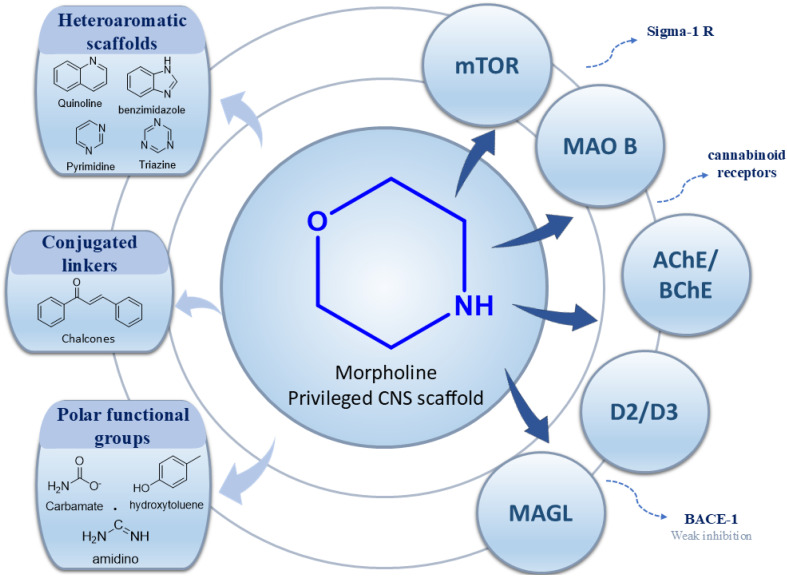
Schematic representation of morpholine as a privileged scaffold in CNS drug discovery. Structural conjugation with heteroaromatic scaffolds, conjugated linkers, and polar functional groups enables modulation of activity across multiple CNS-relevant targets. Targets are positioned relative to the morpholine core based on reported potency and affinity trends, with inner proximity reflecting stronger interactions and outer placement indicating weaker or exploratory activity.

Based on the collective biological activity data discussed across multiple CNS targets, a schematic overview summarising morpholine–centred structure–activity relationships is presented in Fig. 9 (Table 1).

**Table 1:** Summary of IC_50_ values (µM) of morpholine-based compounds against multiple CNS-relevant targets. The listed targets correspond to key neurodegenerative pathways, including monoamine metabolism (MAO-A/B), cholinergic neurotransmission (AChE/BChE), kinase signalling (mTOR), amyloid-β production (β-secretase), endocannabinoid regulation (MAGL), and sigma-1 receptor–mediated neuroprotection. Potency values are reported as IC_50_ or *K*_i_ as provided in the original references. “—” indicates that the corresponding biological evaluation was not reported or not performed in the cited study

Compounds	Structure	IC_50_ (µM)							
		MAO A	MAO B	AChE	BChE	mTOR	β-secretase	MAGL	Sigma1R
1	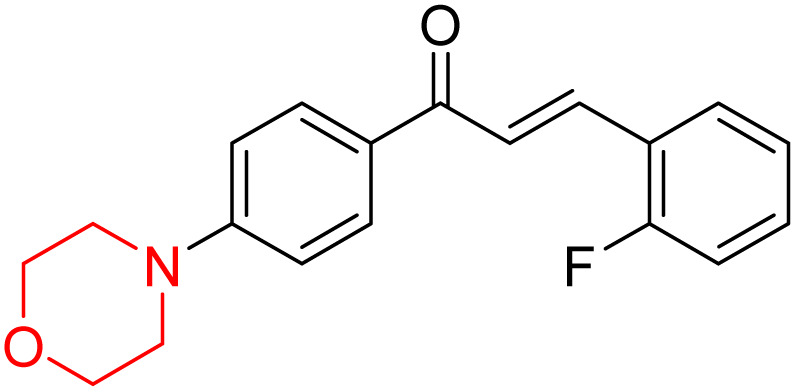	18.69 ± 0.34	0.14 ± 0.005	48.06 ± 0.55	—	—	—	—	—
2	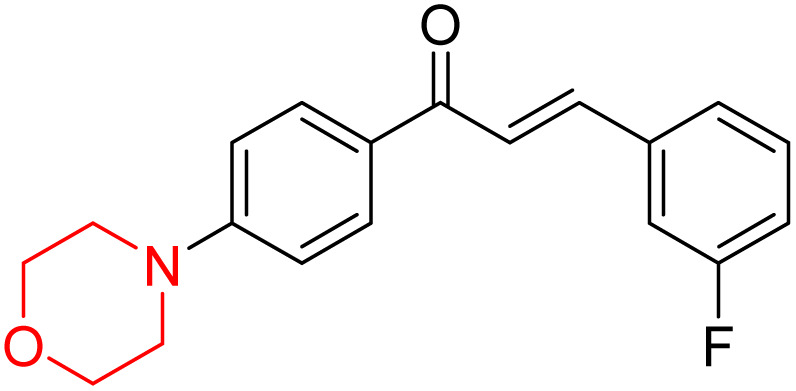	45.0 ± 2.8	0.087 ± 0.008	>50.0	—	—	—	—	—
3	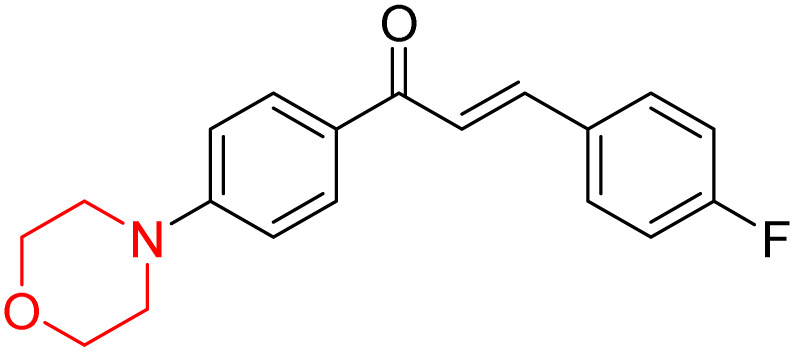	>50.0	0.21 ± 0.006	>50.0	—	—	—	—	—
4	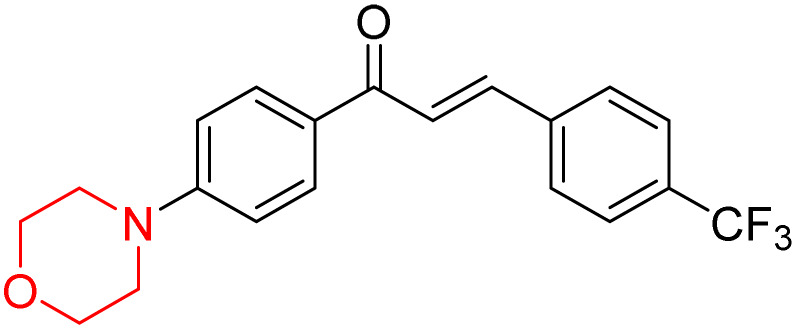	>50.0	2.30 ± 0.20	24.52 ± 0.27	—	—	—	—	—
5	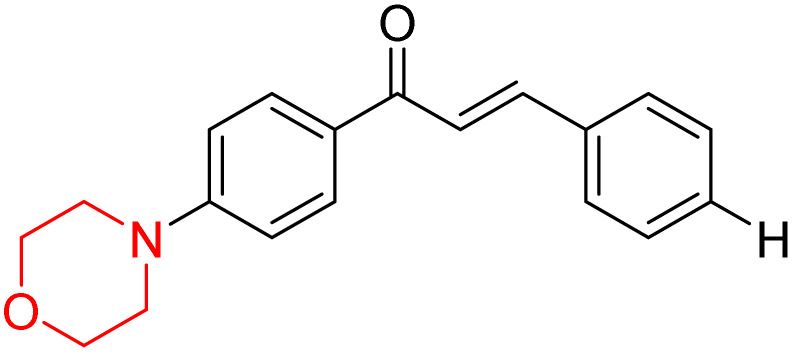	>40	0.030 ± 0.062	16.1 ± 2.24	>40	—	—	—	—
6	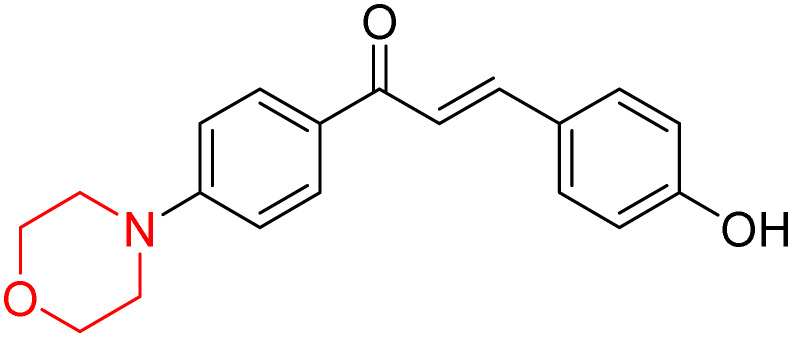	>40	0.70 ± 0.23	30.2 ± 3.24	>40	—	—	—	—
7	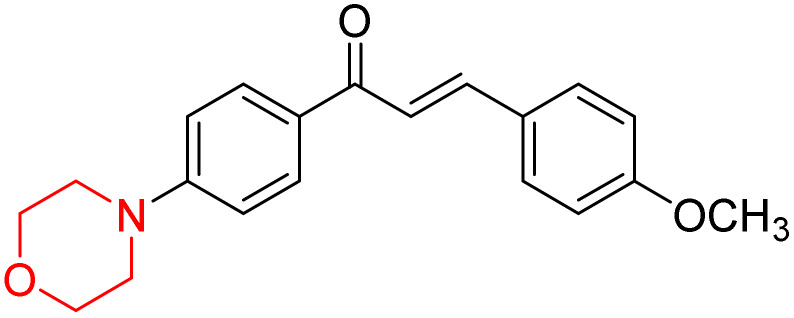	>40	1.01 ± 0.08	>40	>40	—	—	—	—
8	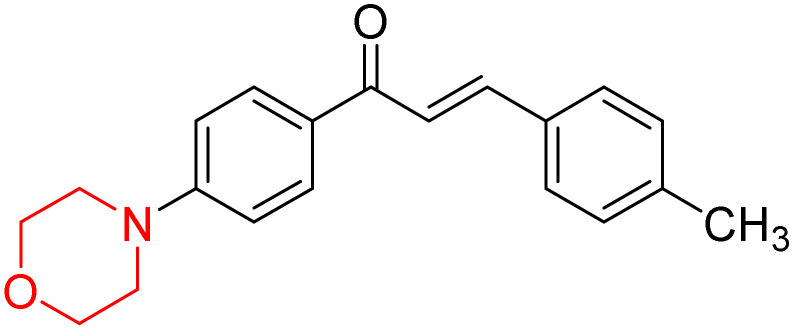	25.8 ± 1.58	0.33 ± 0.03	28.42 ± 0.02	>40	—	—	—	—
9	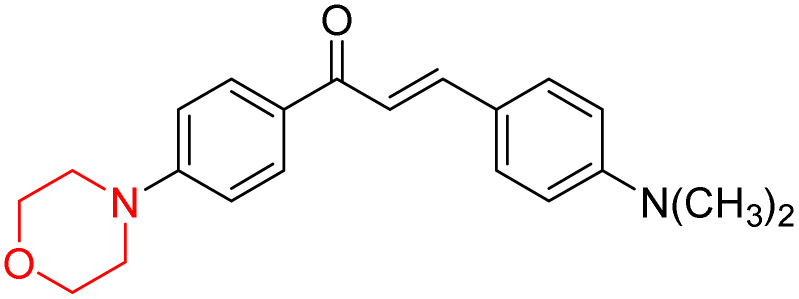	>40	1.31 ± 0.26	6.1 ± 0.0048	18.09 ± 0.38	—	—	—	—
10	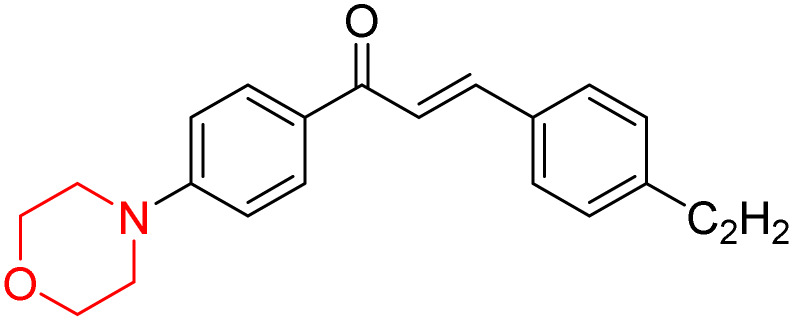	8.7 ± 1.32	0.64 ± 0.04	>40	>40	—	—	—	—
11	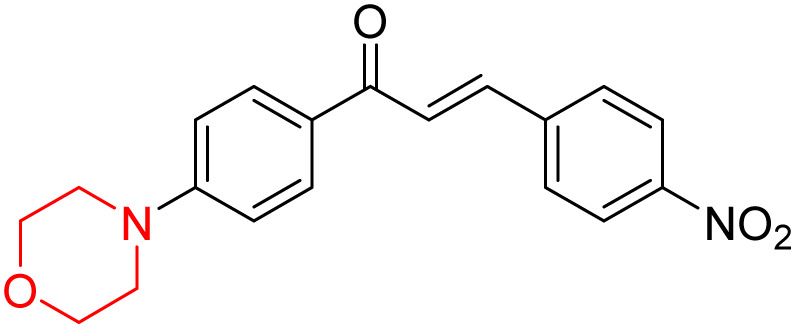	7.1 ± 0.41	0.25 ± 0.05	20.48 ± 1.10	24.83 ± 0.34	—	—	—	—
12	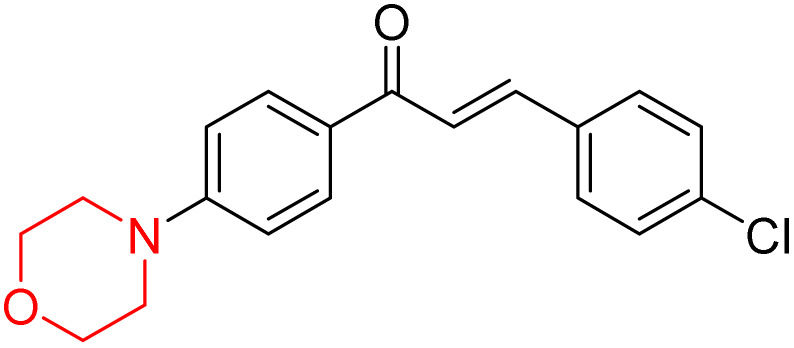	>40	0.32 ± 0.16	12.07 ± 1.18	>40	—	—	—	—
13	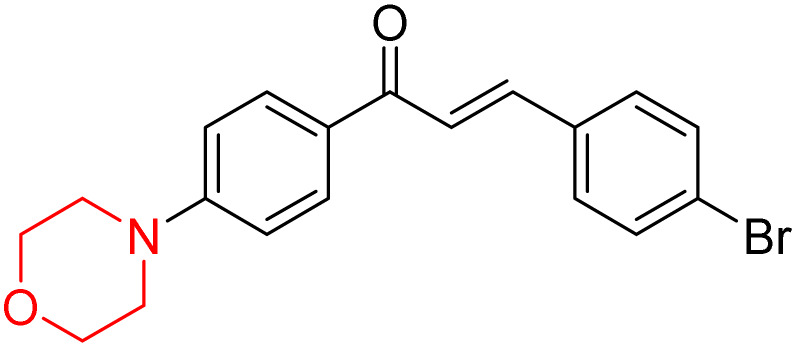	>40	0.36 ± 0.16	12.01 ± 2.13	>40	—	—	—	—
14	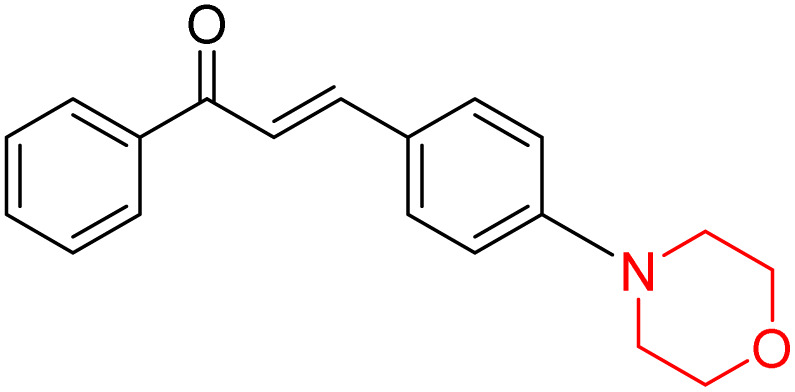	11.97 ± 0.27	0.21 ± 0.010	—	—	—	—	—	—
15	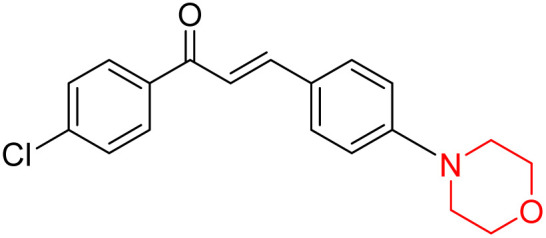	0.82 ± 0.25	0.13 ± 0.01	—	—	—	—	—	—
16	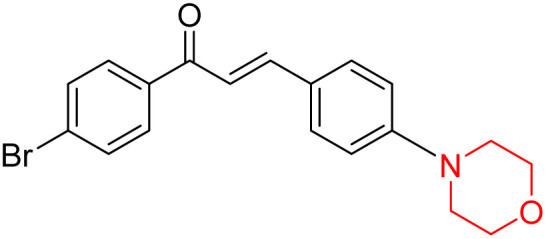	11.67 ± 1.38	0.17 ± 0.04	—	—	—	—	—	—
17	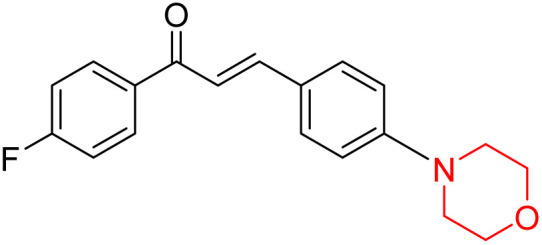	7.61 ± 0.19	0.095 ± 0.012	—	—	—	—	—	—
18	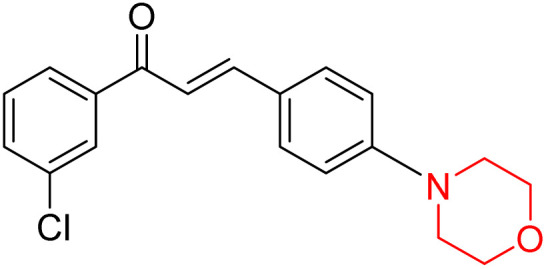	4.30 ± 0.043	0.065 ± 0.014	—	—	—	—	—	—
19	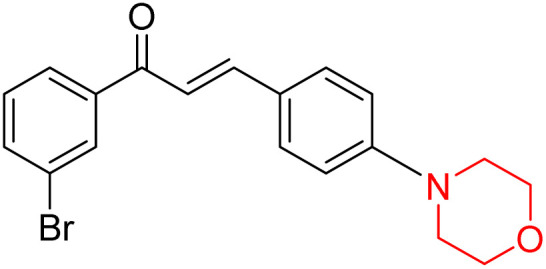	3.04 ± 0.068	0.082 ± 0.012	—	—	—	—	—	—
20	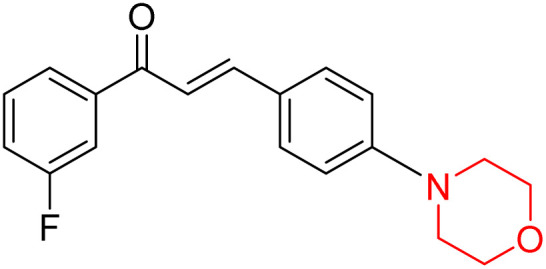	2.89 ± 0.62	0.078 ± 0.007	—	—	—	—	—	—
21	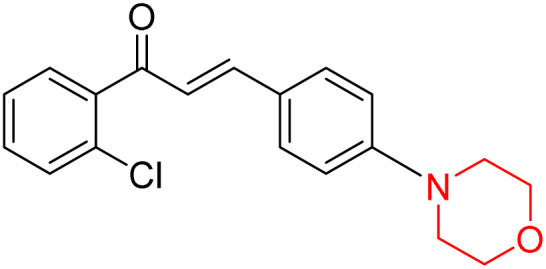	15.37 ± 0.81	0.15 ± 0.02	—	—	—	—	—	—
22	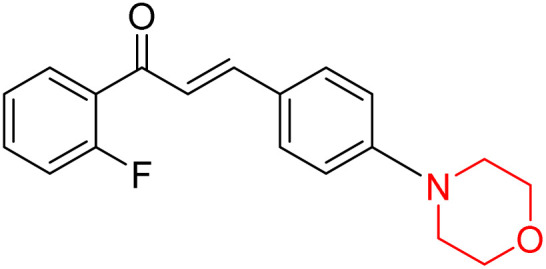	4.28 ± 0.052	0.13 ± 0.05	—	—	—	—	—	—
23	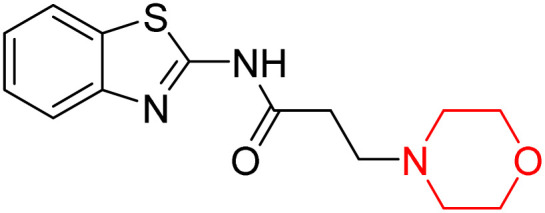	—	—	>200	>200	—	—	—	—
24	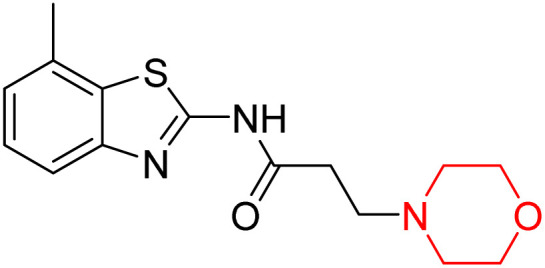	—	—	>200	>200	—	—	—	—
25	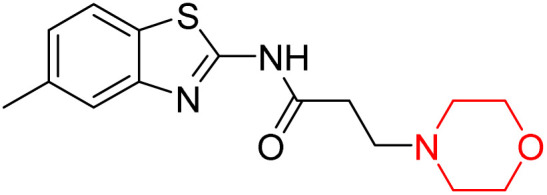	—	—	>200	>200	—	—	—	—
26	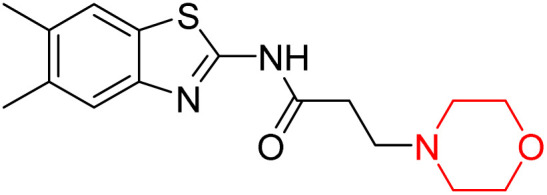	—	—	>200	>200	—	—	—	—
27	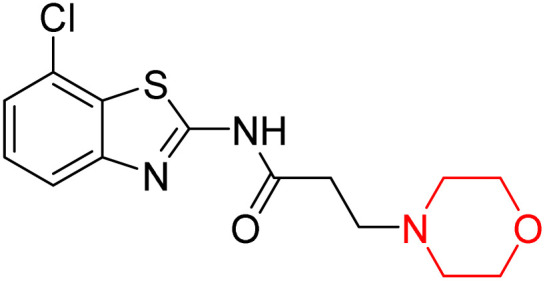	—	—	>200	>200	—	—	—	—
28	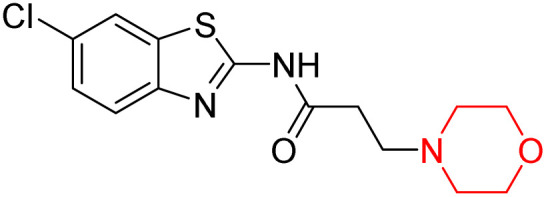	—	—	>200	>200	—	—	—	—
29	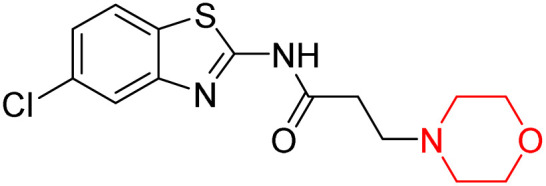	—	—	>200	>200	—	—	—	—
30	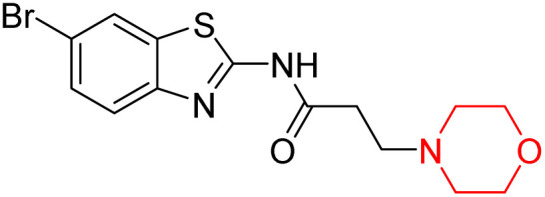	—	—	>200	>200	—	—	—	—
31	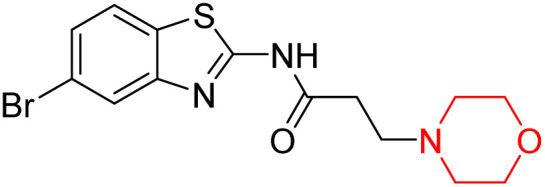	—	—	>200	>200	—	—	—	—
32	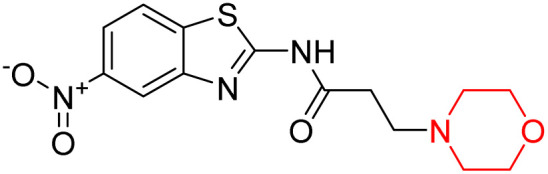	—	—	>200	>200	—	—	—	—
33	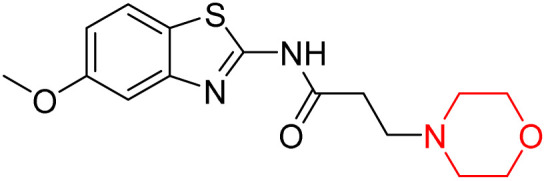	—	—	>200	>200	—	—	—	—
34	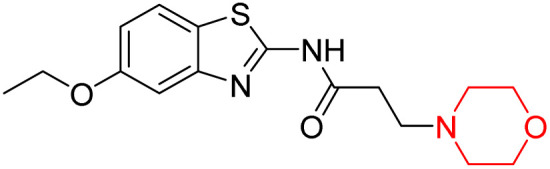	—	—	>200	>200	—	—	—	—
35	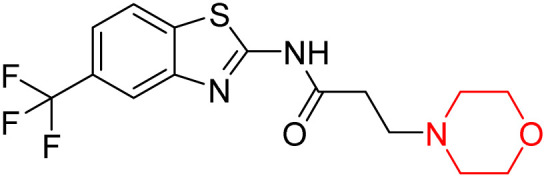	—	—	>200	>200	—	—	—	—
36	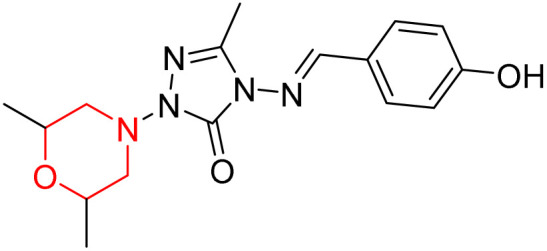	—	—	51.20	38.79	—	—	—	—
37	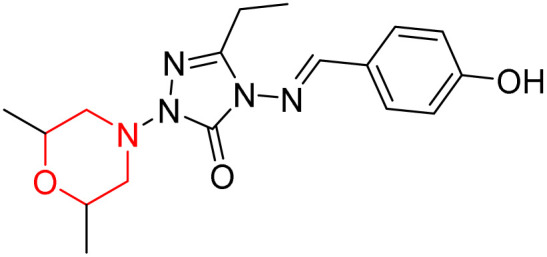	—	—	44.18	40.93	—	—	—	—
38	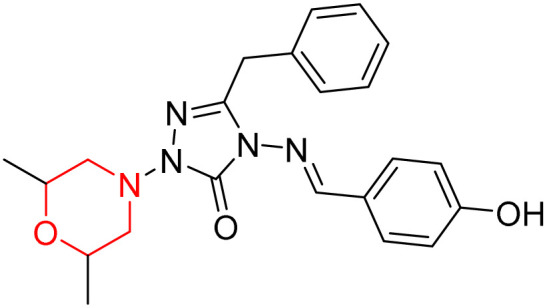	—	—	50.84	38.07	—	—	—	—
39	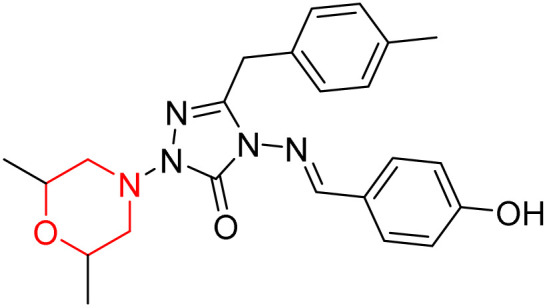	—	—	49.23	34.97	—	—	—	—
40	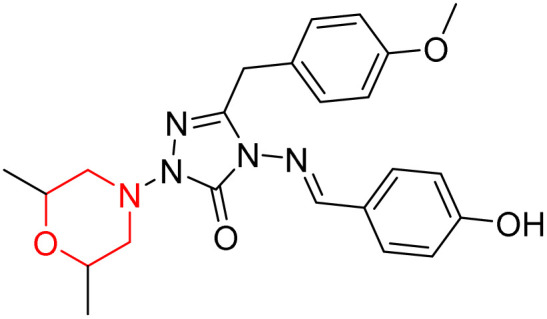	—	—	35.36	44.75	—	—	—	—
41	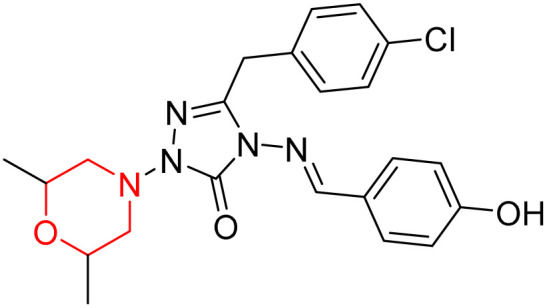	—	—	41.73	42.80	—	—	—	—
42	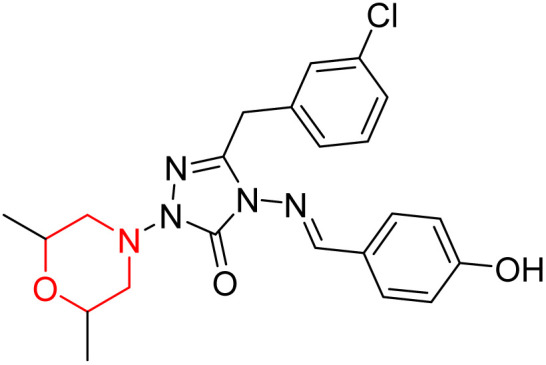	—	—	55.31	43.73	—	—	—	—
43	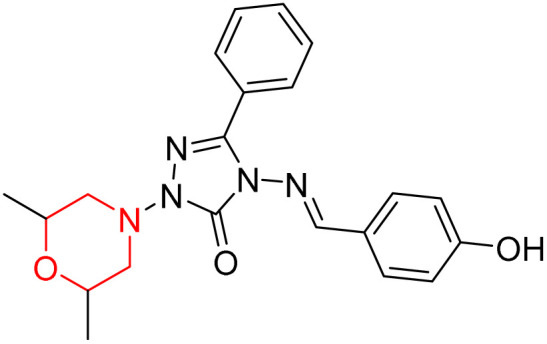	—	—	35.29	44.49	—	—	—	—
44	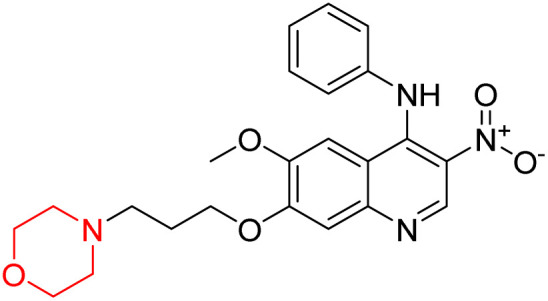	—	—	4.94 ± 0.15	80.91 ± 2.31	—	—	—	—
45	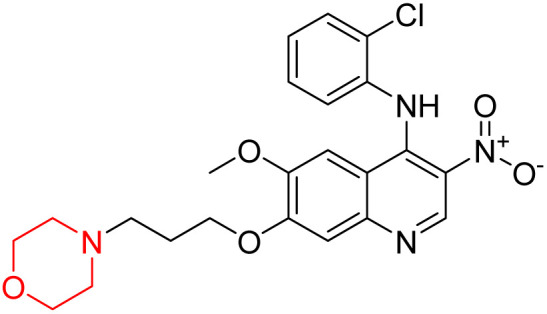	—	—	12.51 ± 0.21	130.38 ± 4.62	—	—	—	—
46	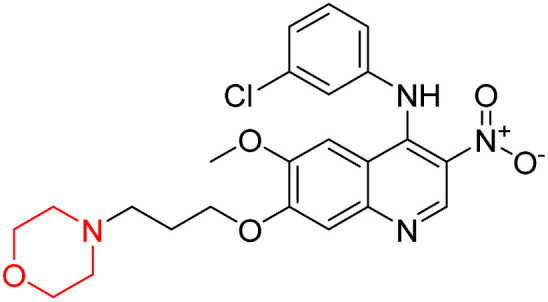	—	—	32.40 ± 0.16	114.12 ± 5.36	—	—	—	—
47	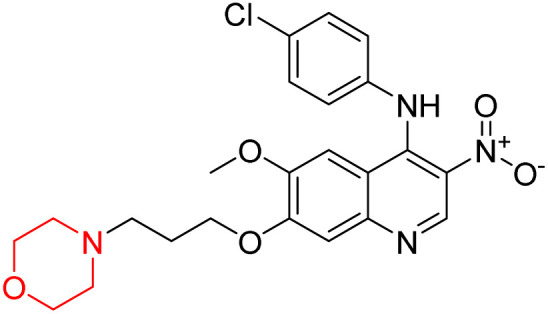	—	—	88.68 ± 1.10	82.39 ± 3.06	—	—	—	—
48	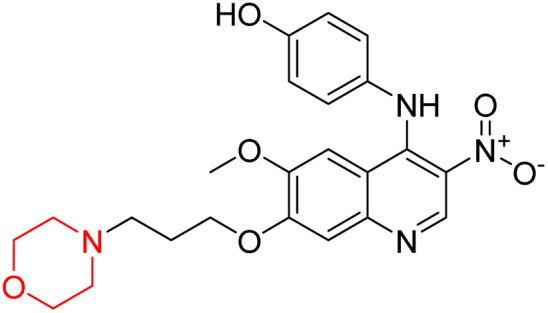	—	—	34.47 ± 0.09	>150	—	—	—	—
49	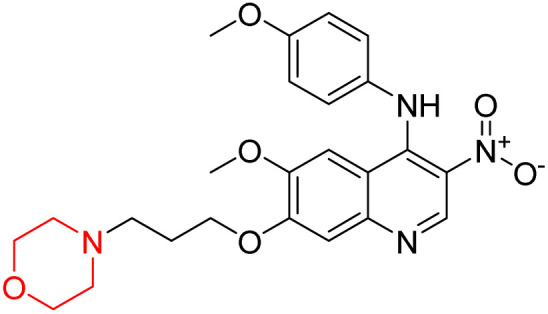	—	—	12.80 ± 0.55	96.60 ± 4.75	—	—	—	—
50	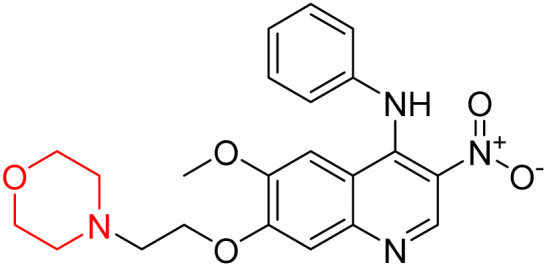	—	—	1.94 ± 0.13	28.37 ± 1.85	—	—	—	—
51	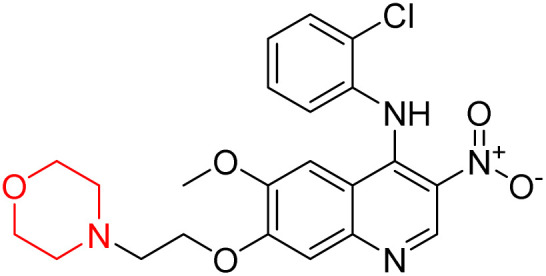	—	—	6.46 ± 0.77	81.08 ± 4.03	—	—	—	—
52	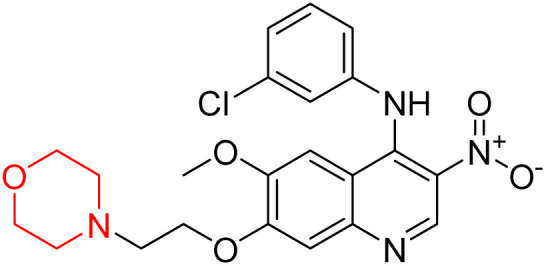	—	—	10.01 ± 0.52	110.96 ± 5.59	—	—	—	—
53	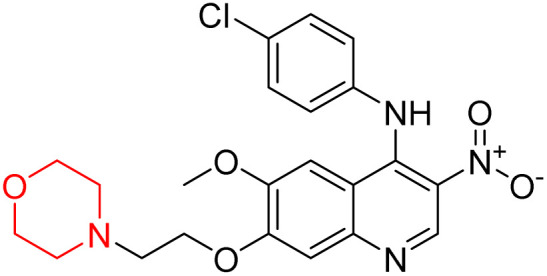	—	—	8.63 ± 0.40	76.81 ± 3.76	—	—	—	—
54	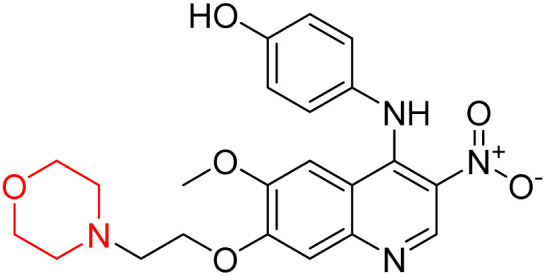	—	—	18.49 ± 1.74	>150	—	—	—	—
55	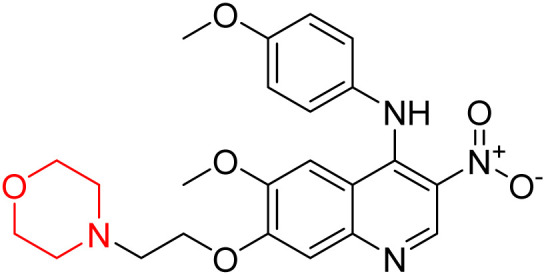	—	—	9.08 ± 0.53	63.92 ± 2.87	—	—	—	—
56	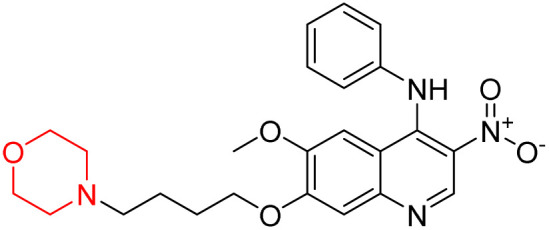	—	—	13.34 ± 0.07	>150	—	—	—	—
57	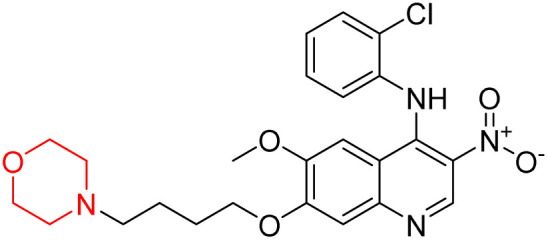	—	—	85.57 ± 0.04	>150	—	—	—	—
58	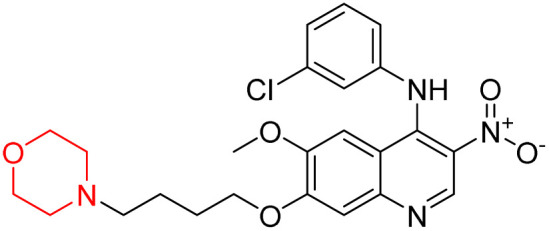	—	—	>150	116.25 ± 5.36	—	—	—	—
59	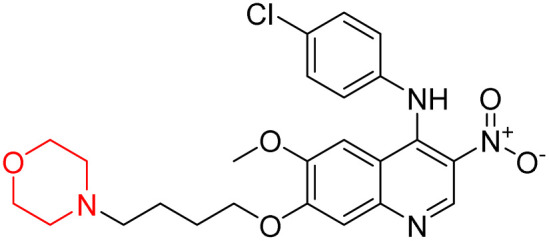	—	—	90.46 ± 0.06	98.10 ± 0.02	—	—	—	—
60	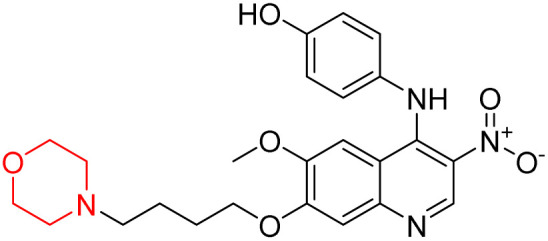	—	—	36.81 ± 0.45	>150	—	—	—	—
61	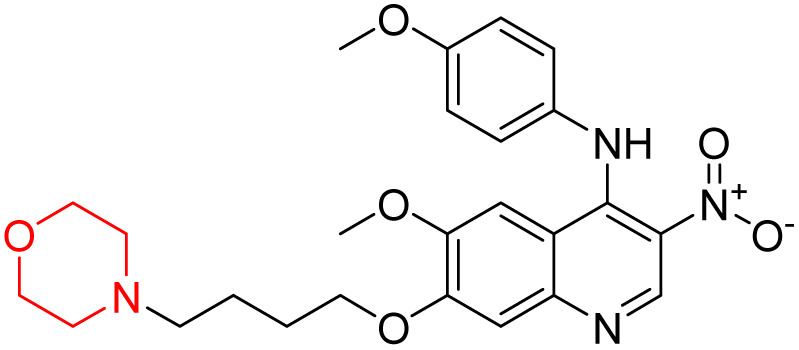	—	—	18.92 ± 0.51	106.49 ± 1.16	—	—	—	—
62	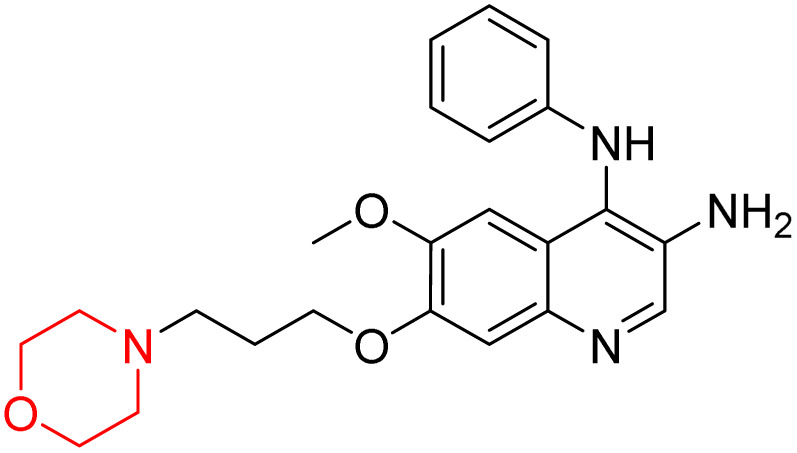	—	—	9.13 ± 0.42	30.53 ± 2.48	—	—	—	—
63	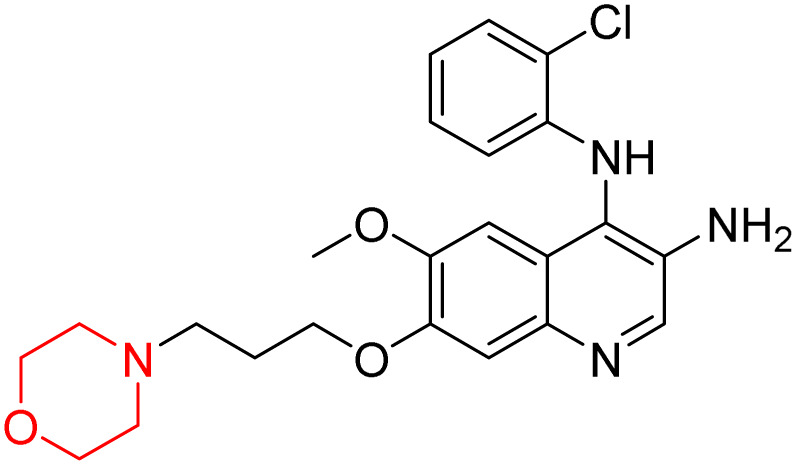	—	—	43.28 ± 0.68	130.10 ± 3.76	—	—	—	—
64	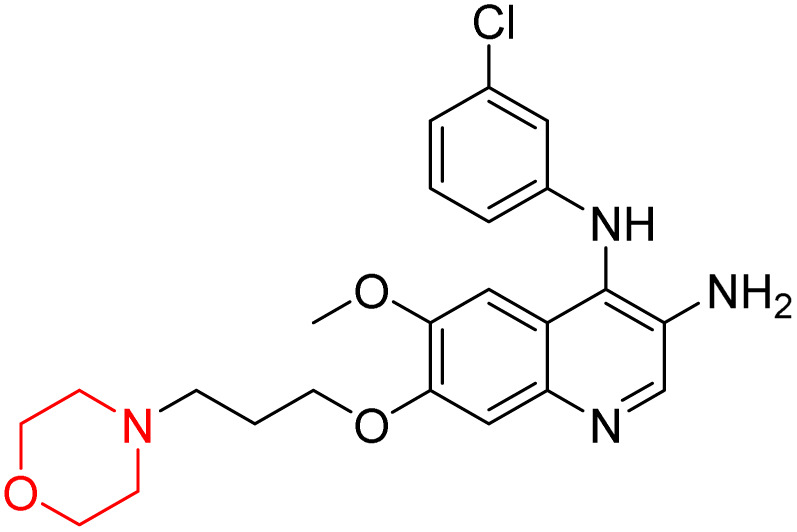	—	—	22.06 ± 0.33	27.84 ± 2.16	—	—	—	—
65	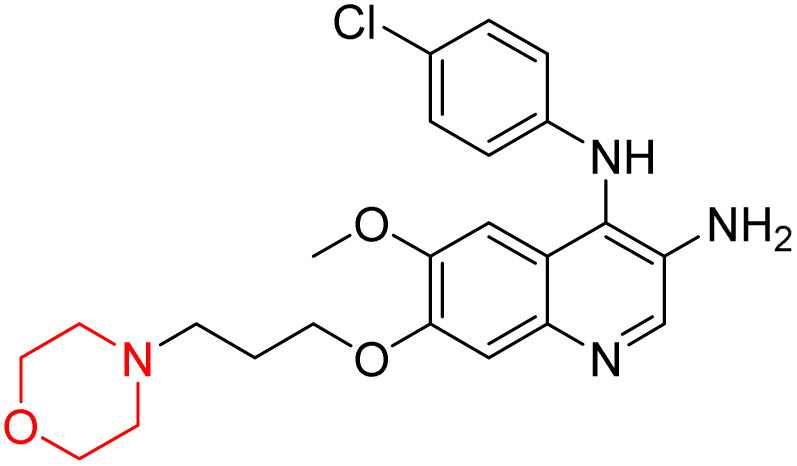	—	—	21.84 ± 0.20	145.00 ± 4.25	—	—	—	—
66	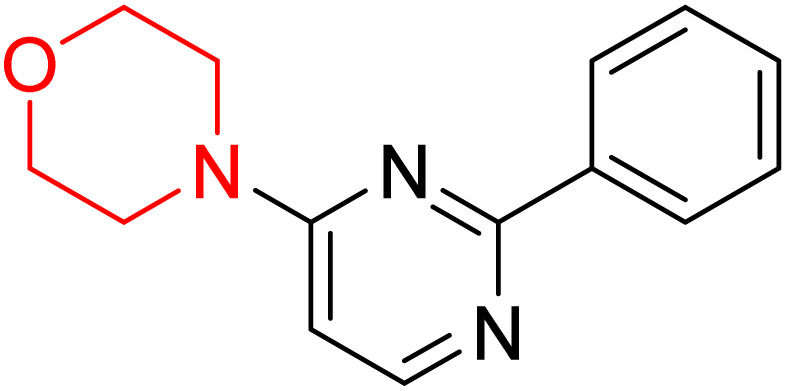	—	—	8.66 ± 0.52	32	—	—	—	—
67	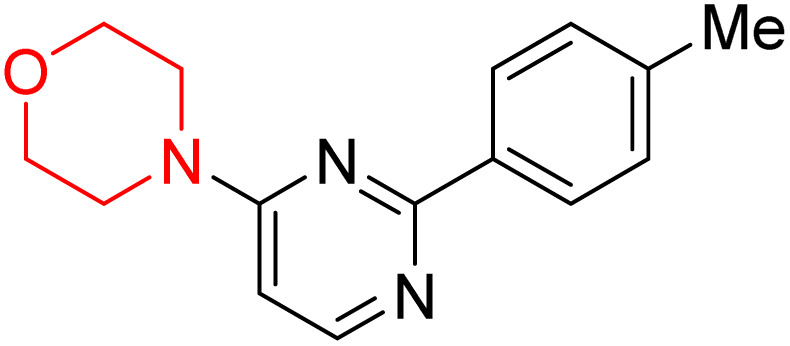	—	—	5.6 ± 0.42	37	—	—	—	—
68	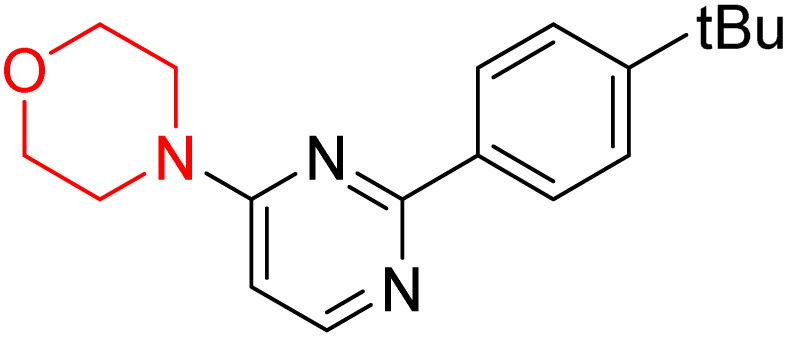	—	—	3.4 ± 0.21	40	—	—	—	—
69	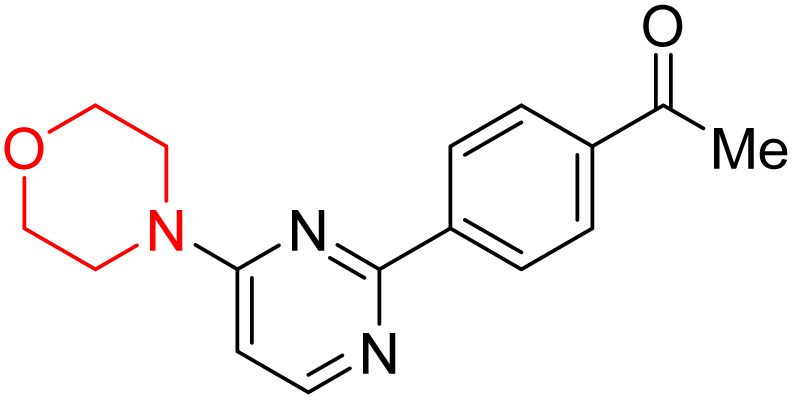	—	—	1.4 ± 0.11	17	—	—	—	—
70	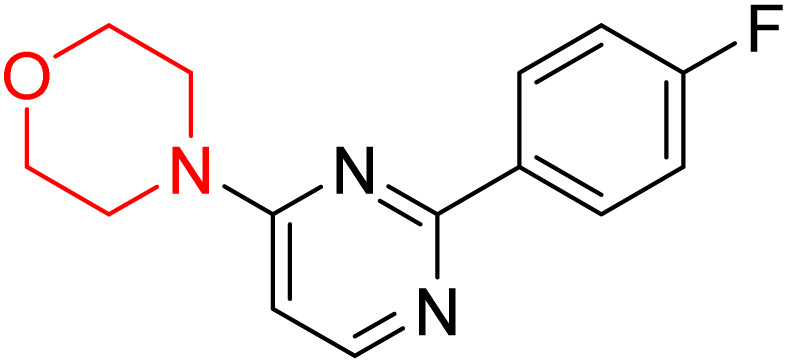	—	—	0.78 ± 0.11	23	—	—	—	—
71	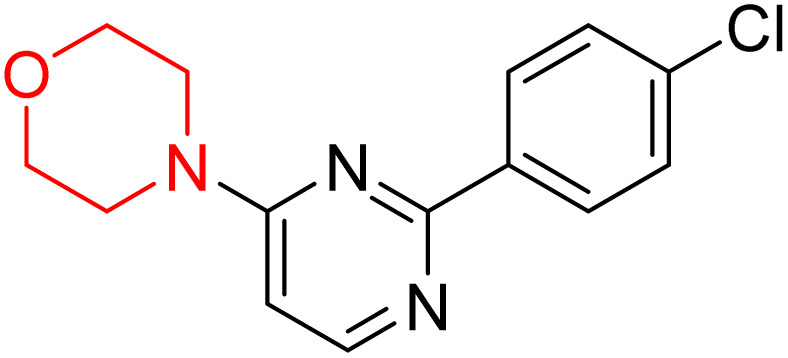	—	—	2.8 ± 0.12	43	—	—	—	—
72	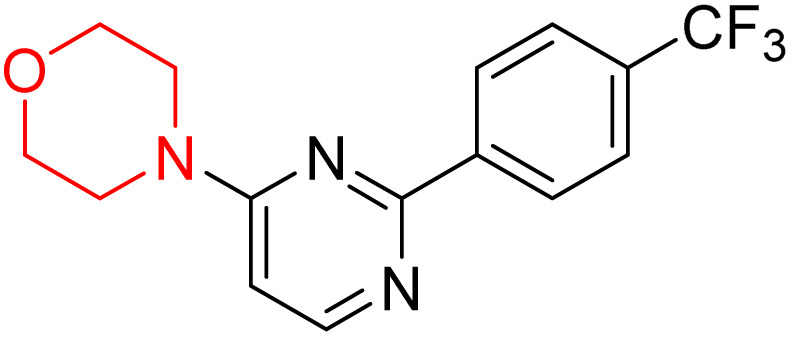	—	—	3.9 ± 0.22	27	—	—	—	—
73	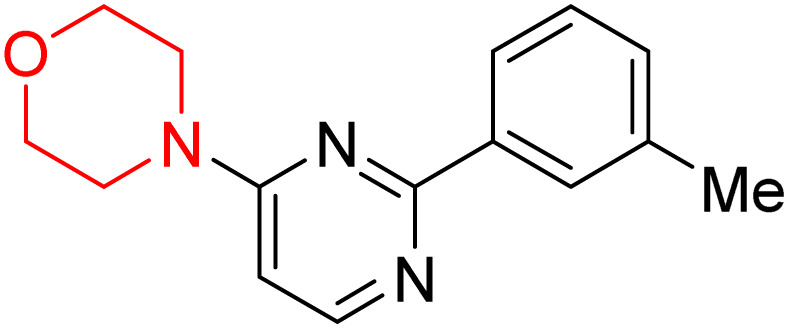	—	—	0.43 ± 0.02	2.5 ± 0.12	—	—	—	—
74	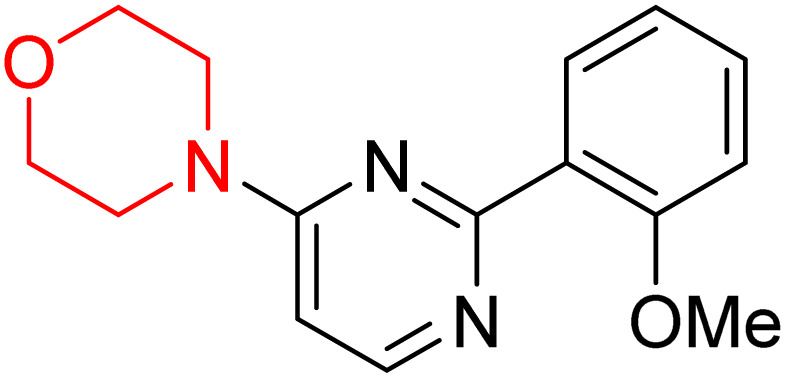	—	—	6.2 ± 0.47	8.9 ± 0.62	—	—	—	—
75	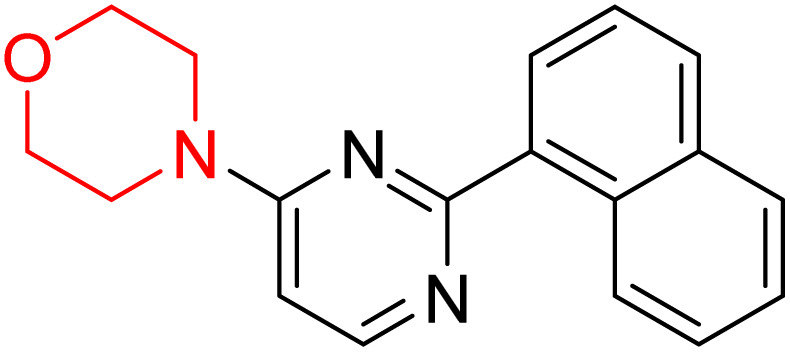	—	—	6.1 ± 0.47	28.7 ± 2.23	—	—	—	—
76	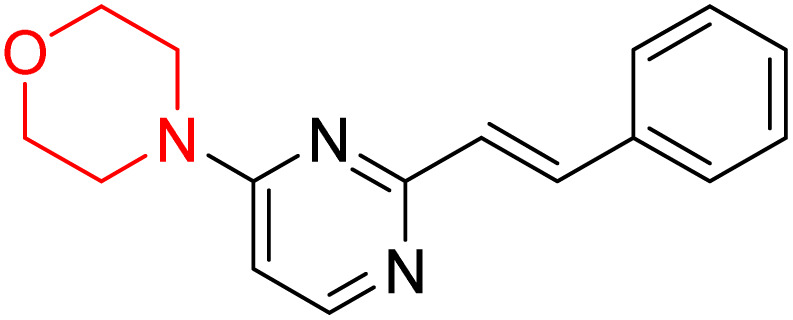	—	—	2.9 ± 0.22	13.4 ± 0.22	—	—	—	—
77	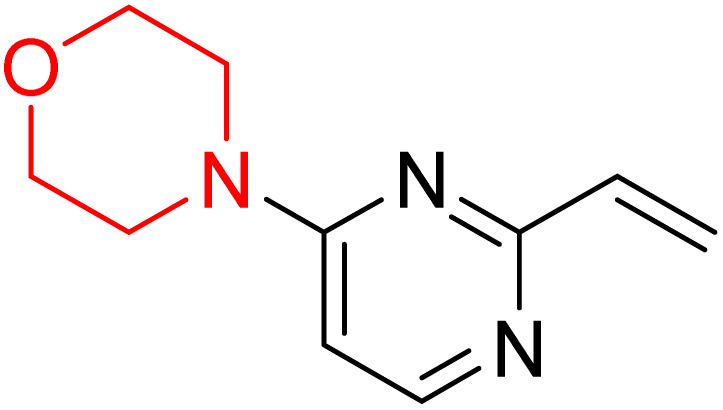	—	—	5.0 ± 0.40	34	—	—	—	—
78	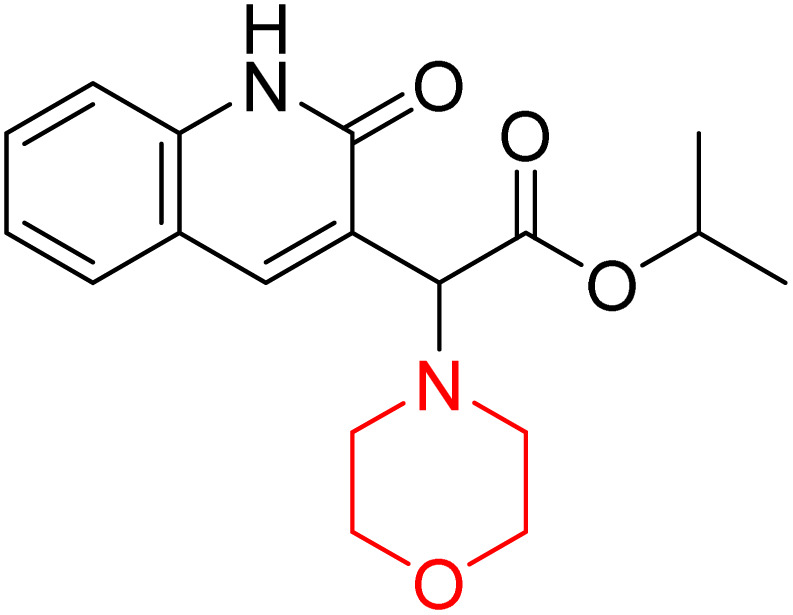	—	—	—	—	—	—	—	—
79	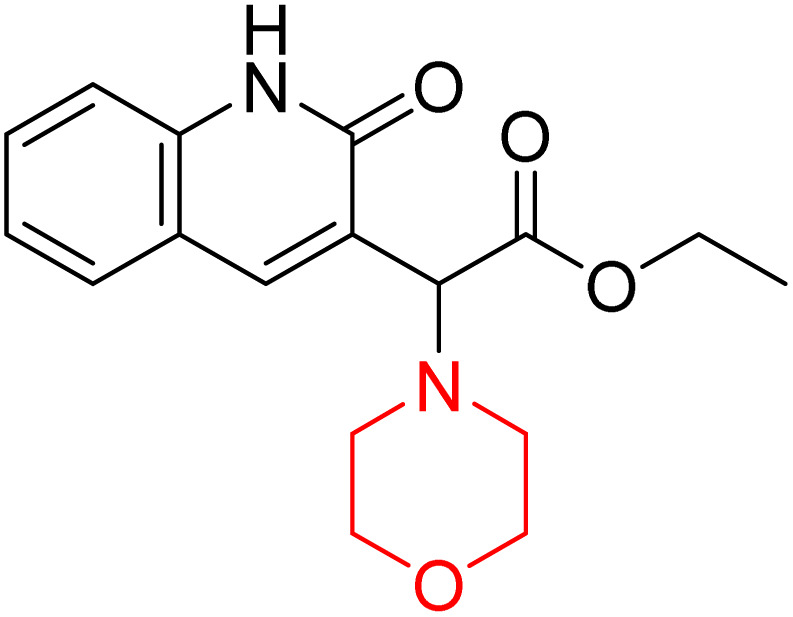	—	—	—	—	—	—	—	—
80	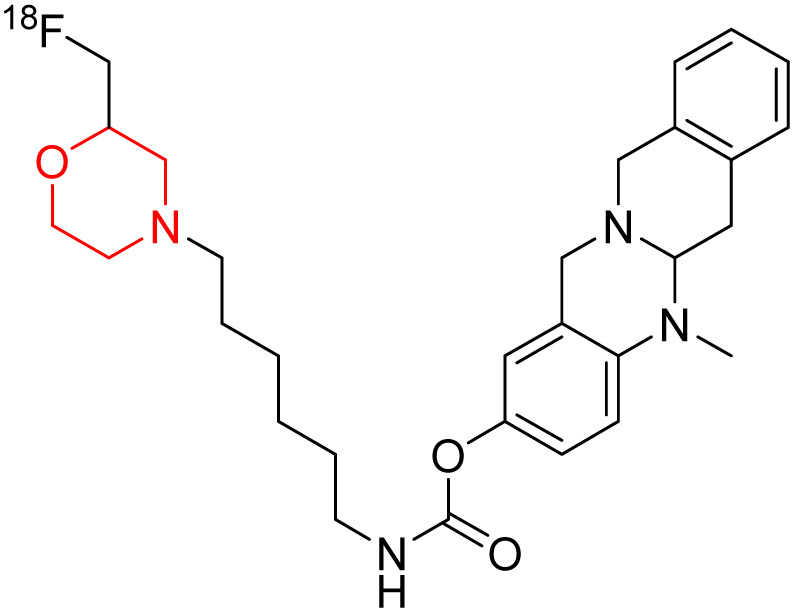	—	—	—	0.066	—	—	—	—
81	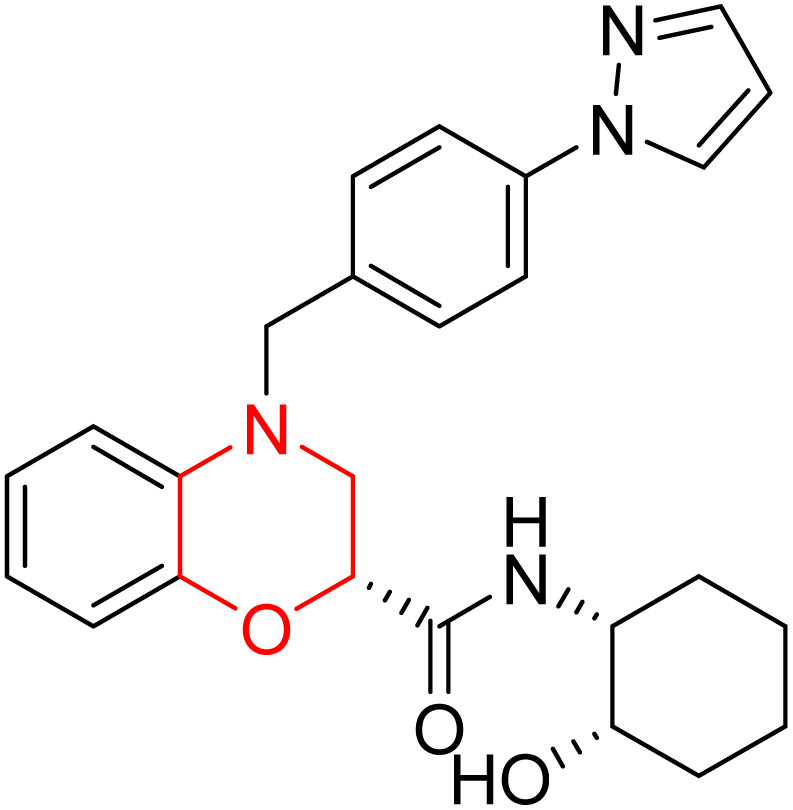	—	—	—	—	—	—	—	—
82	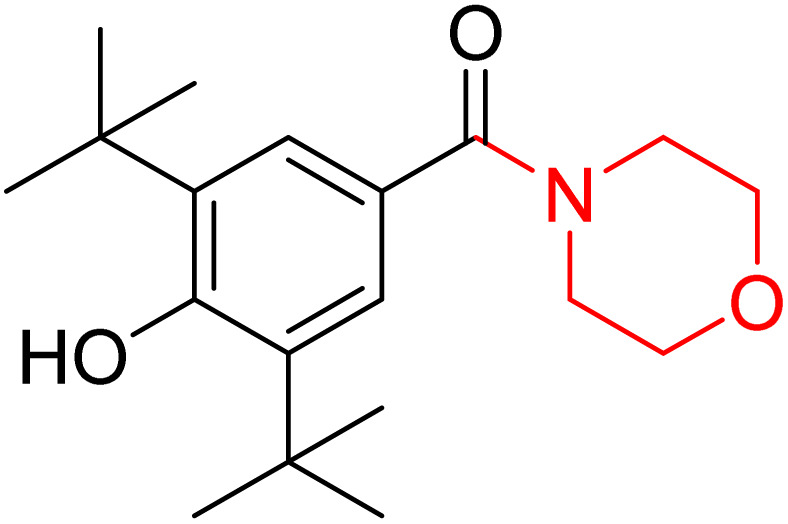	—	—	—	—	—	—	—	—
83	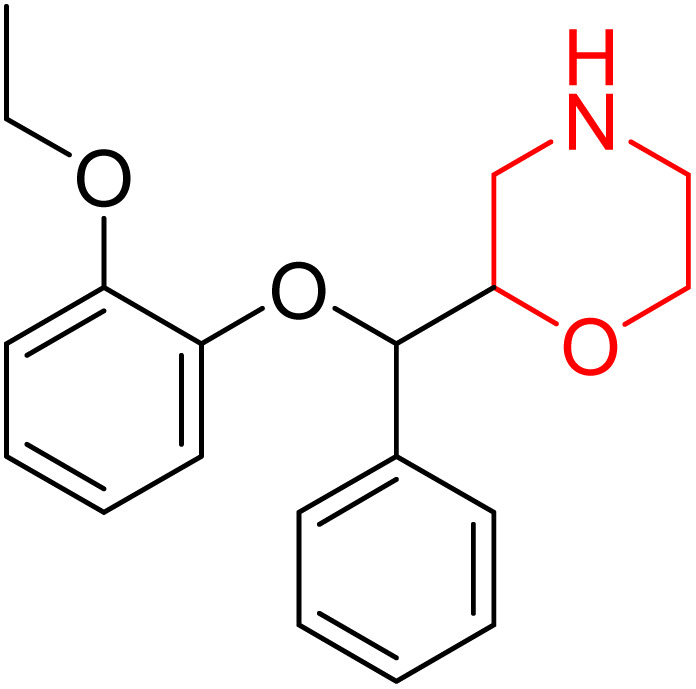	—	—	—	—	—	—	—	—
84	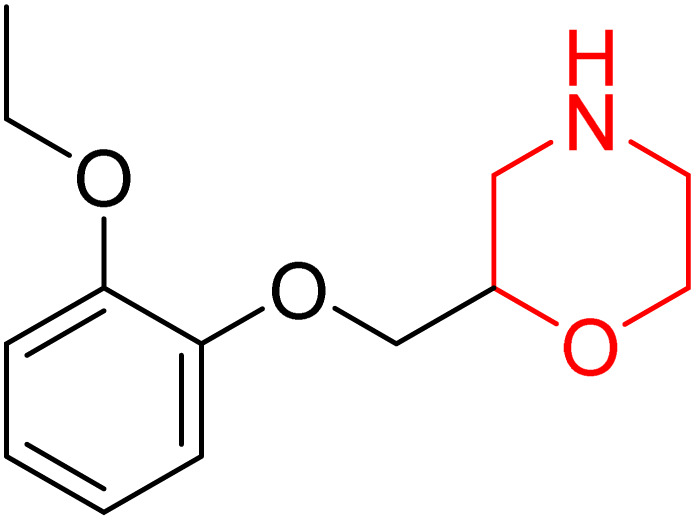	—	—	—	—	—	—	—	—
85	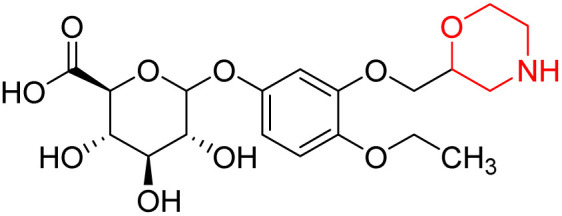	—	—	—	—	—	—	—	—
86	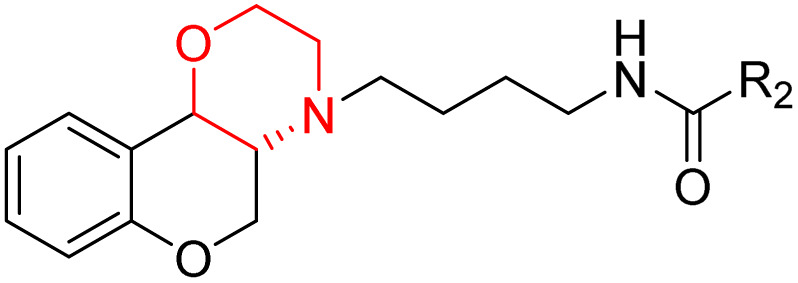	—	—	—	—	—	—	—	—
87	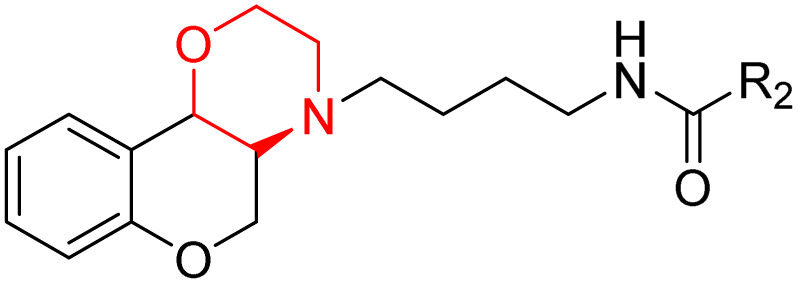	—	—	—	—	—	—	—	—
88	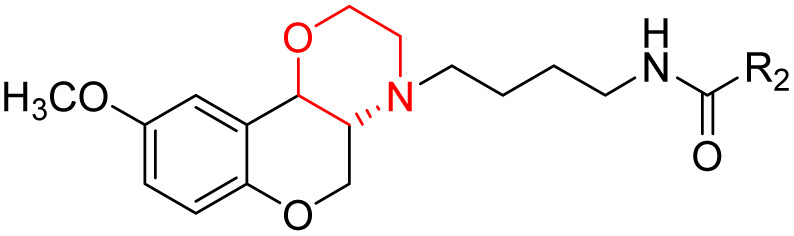	—	—	—	—	—	—	—	—
89	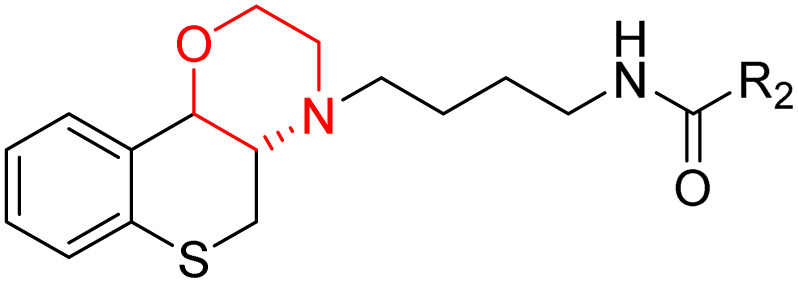	—	—	—	—	—	—	—	—
90	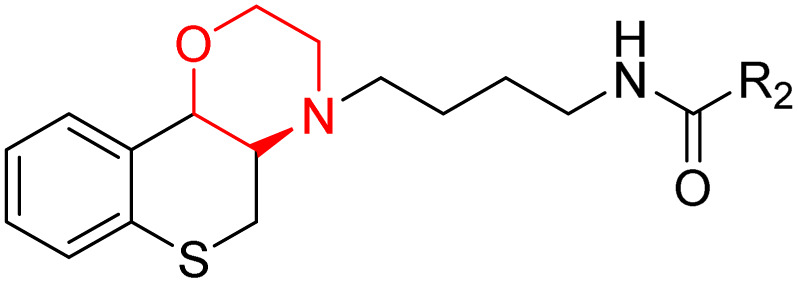	—	—	—	—	—	—	—	—
91	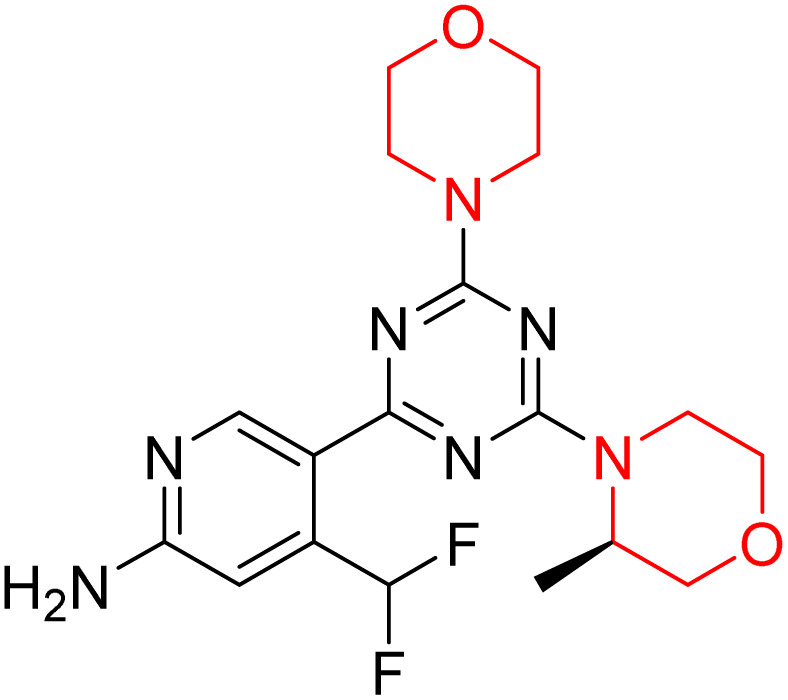	—	—	—	—	0.012	—	—	—
92	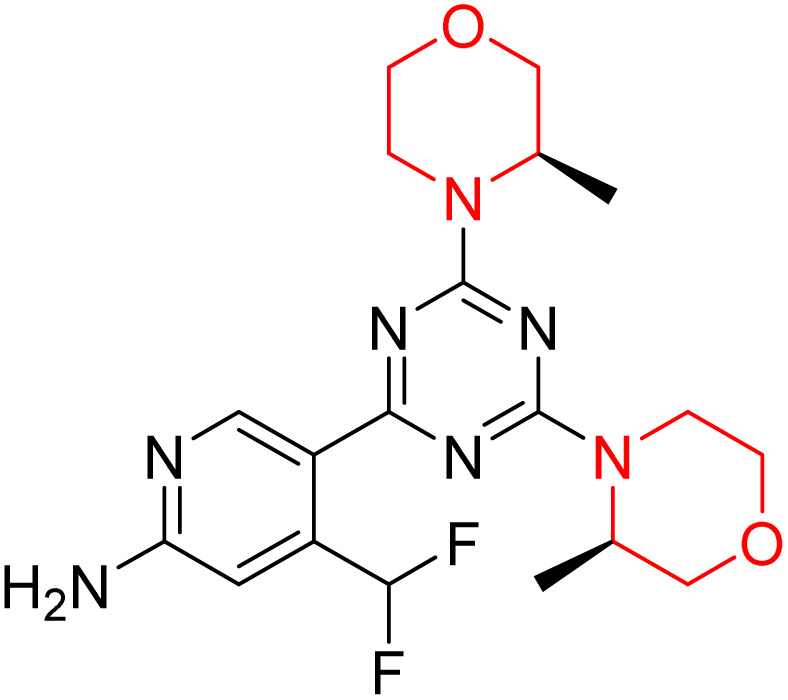	—	—	—	—	0.021	—	—	—
93	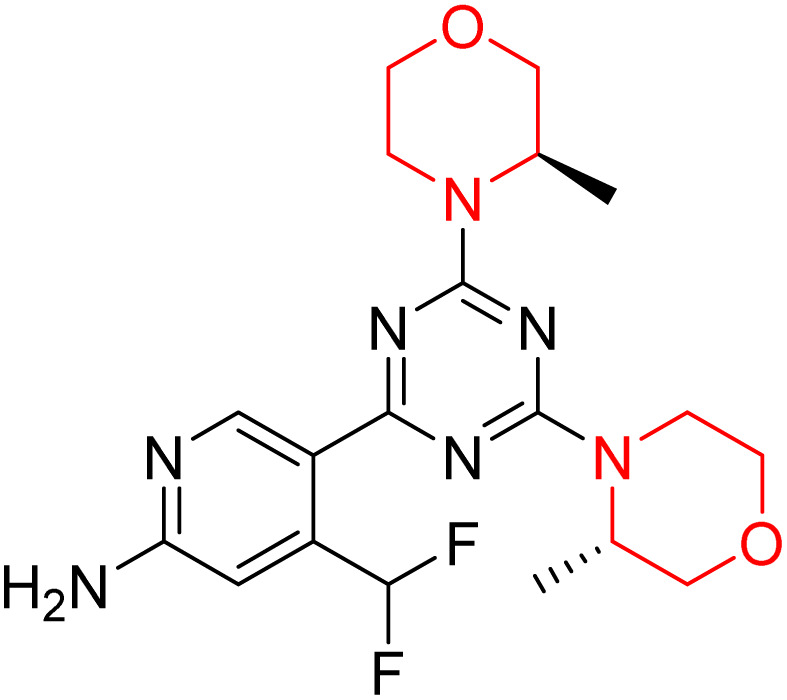	—	—	—	—	0.007	—	—	—
94	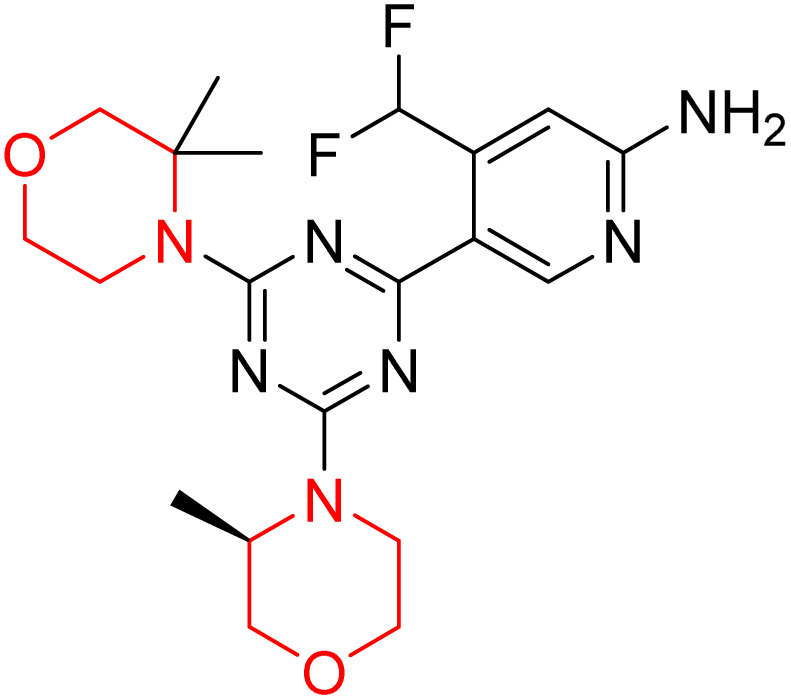	—	—	—	—	0.017	—	—	—
95	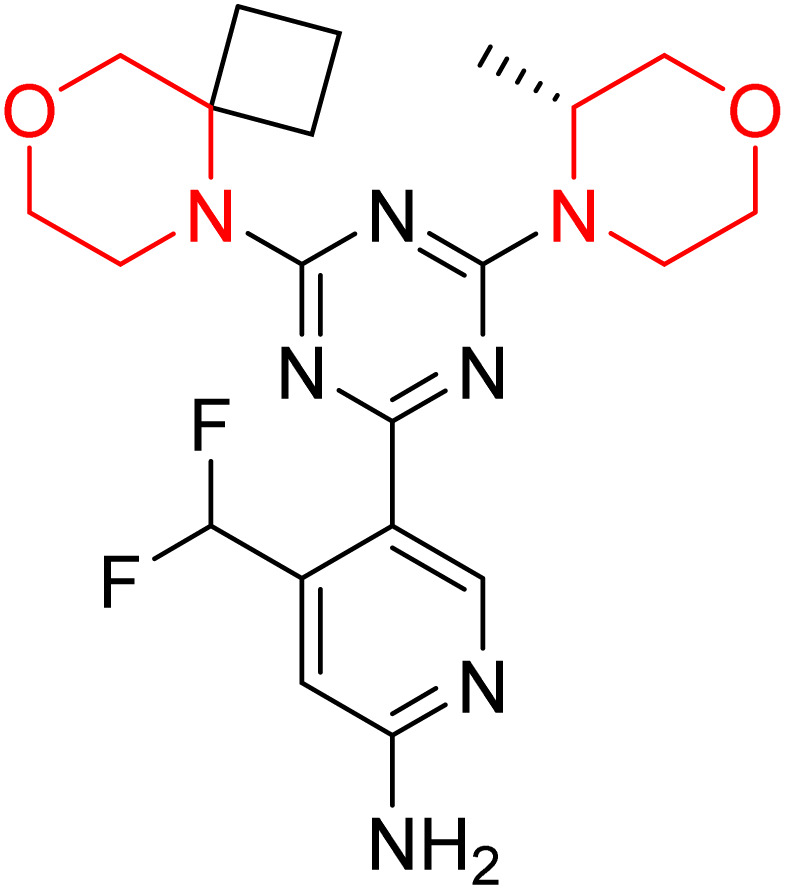	—	—	—	—	0.038	—	—	—
96	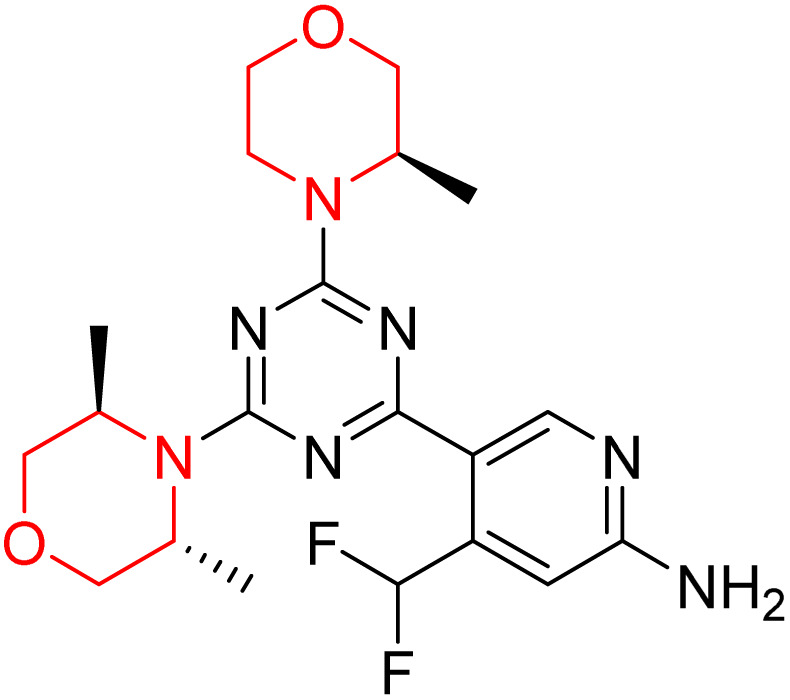	—	—	—	—	0.009	—	—	—
97	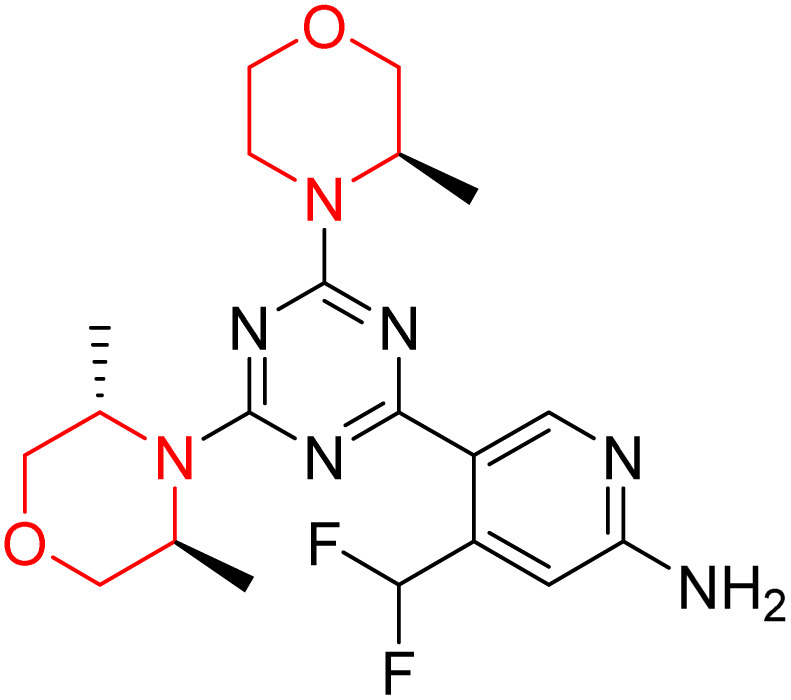	—	—	—	—	0.005	—	—	—
98	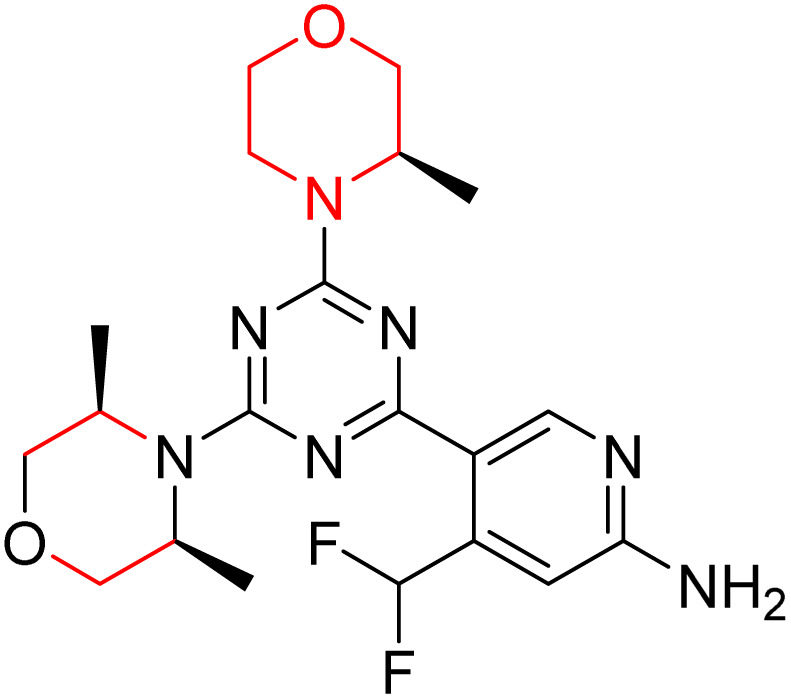	—	—	—	—	0.064	—	—	—
99	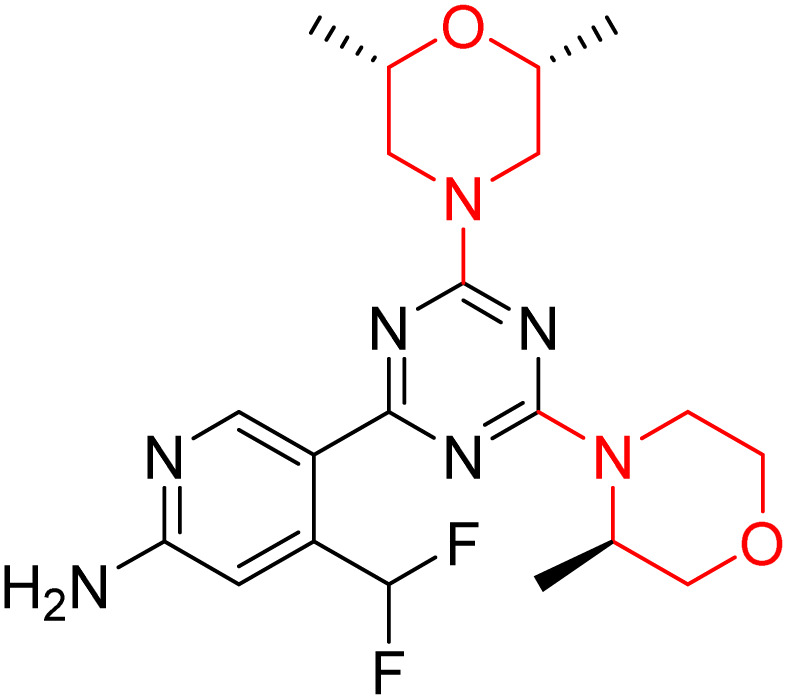	—	—	—	—	0.027	—	—	—
100	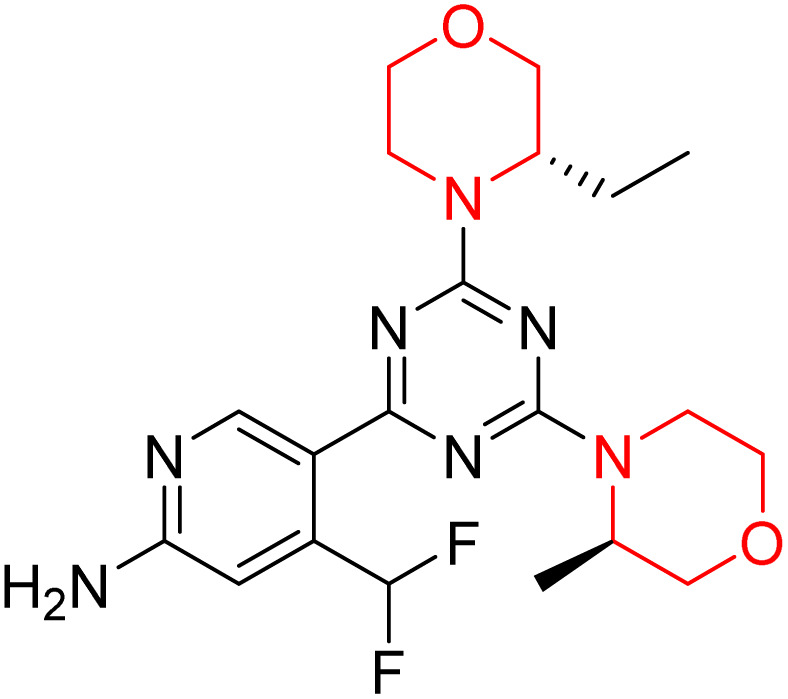	—	—	—	—	0.101	—	—	—
101	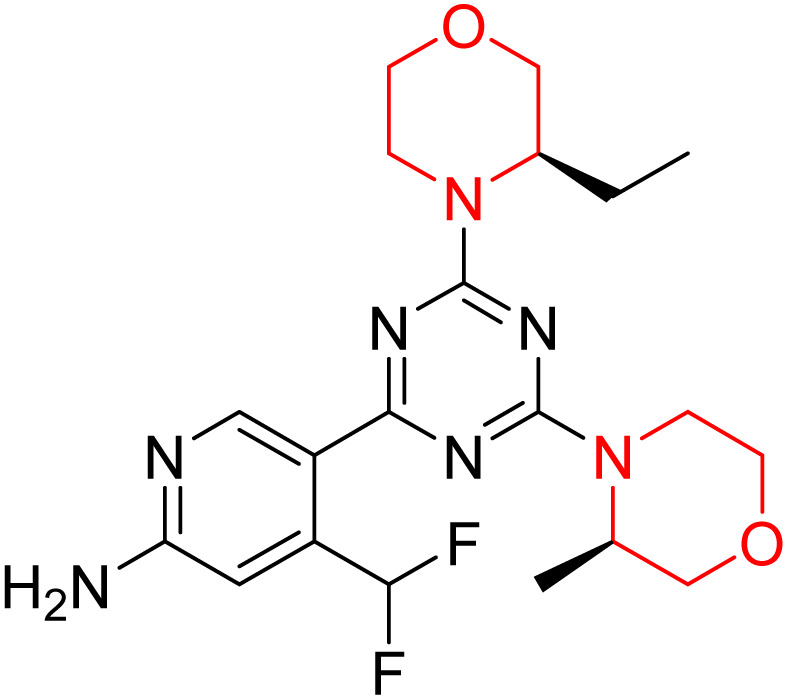	—	—	—	—	0.075	—	—	—
102	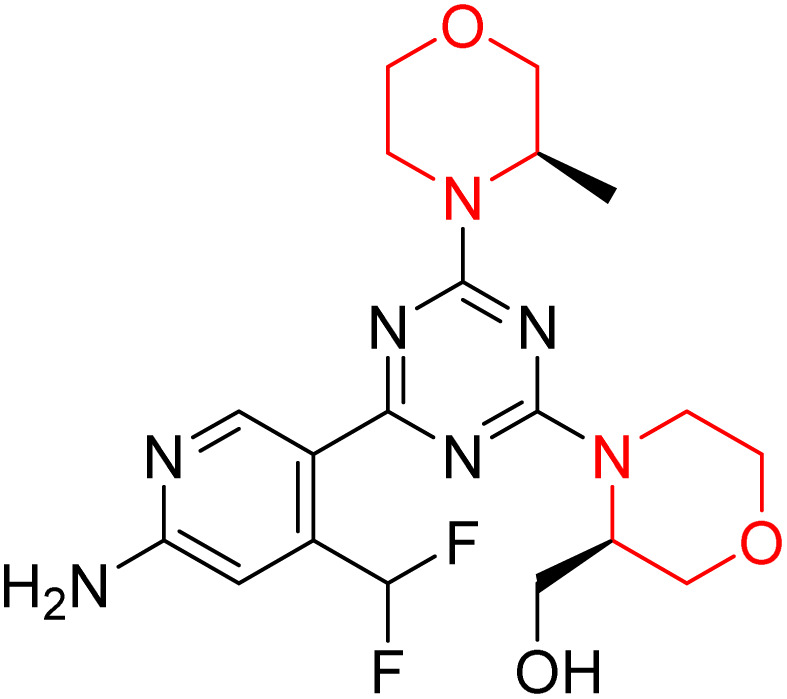	—	—	—	—	0.057	—	—	—
103	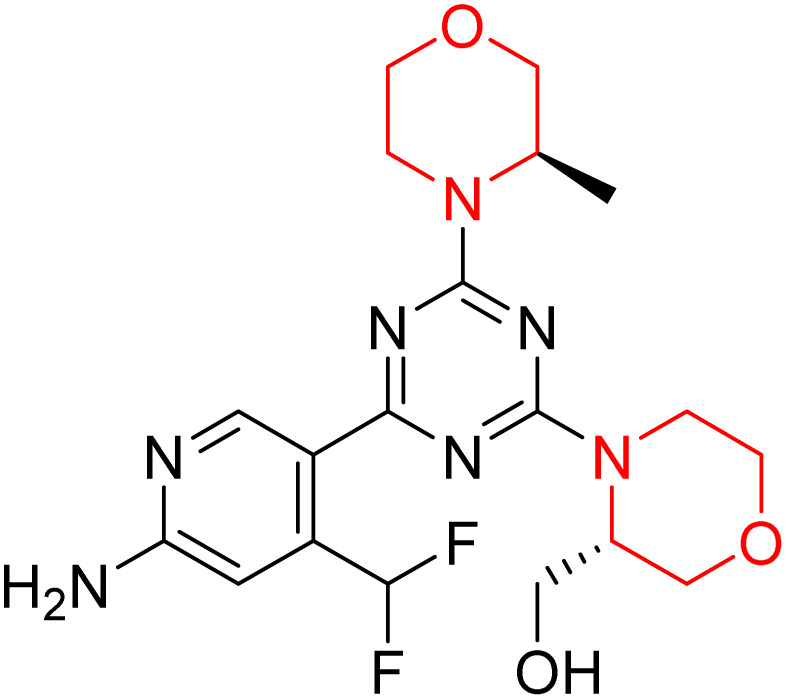	—	—	—	—	0.035	—	—	—
104	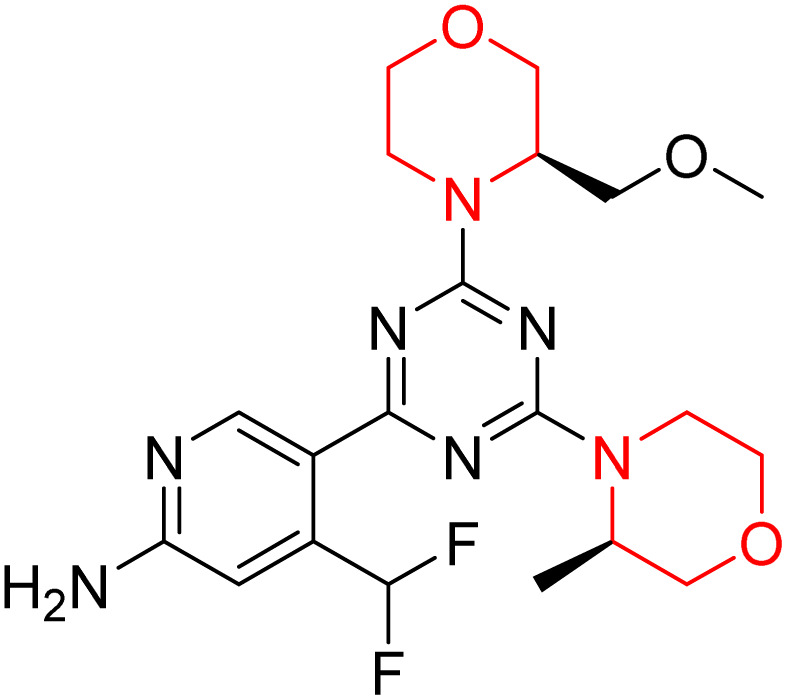	—	—	—	—	0.151	—	—	—
105	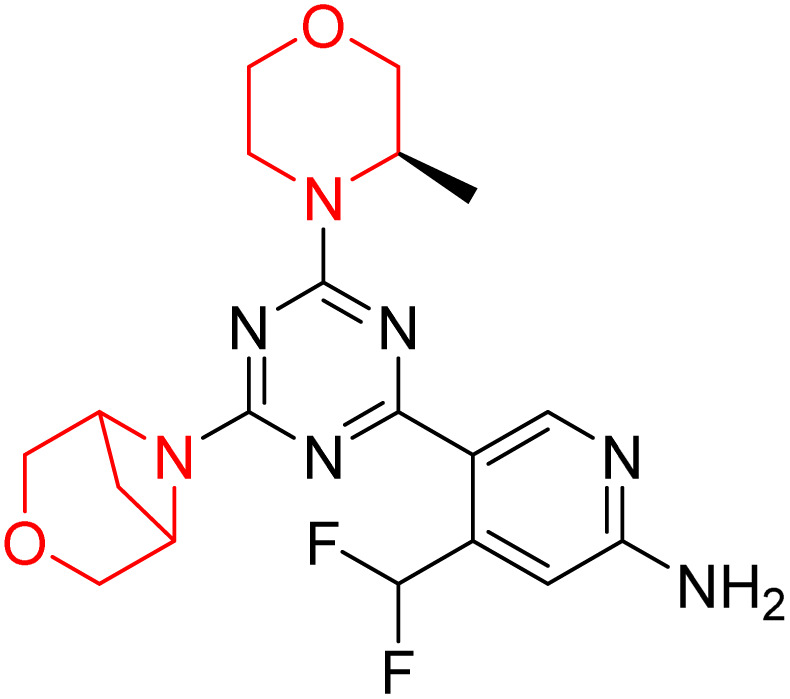	—	—	—	—	0.156	—	—	—
106	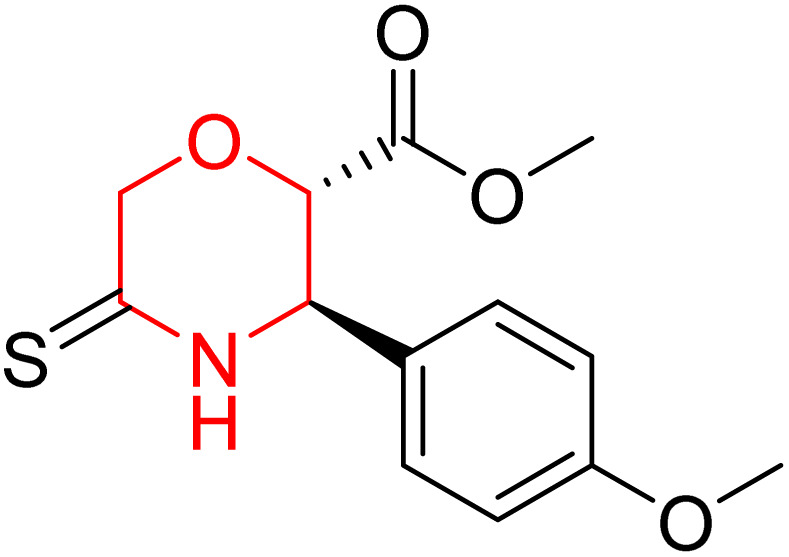	—	—	—	—	—	5.7 ± 1.1	—	—
107	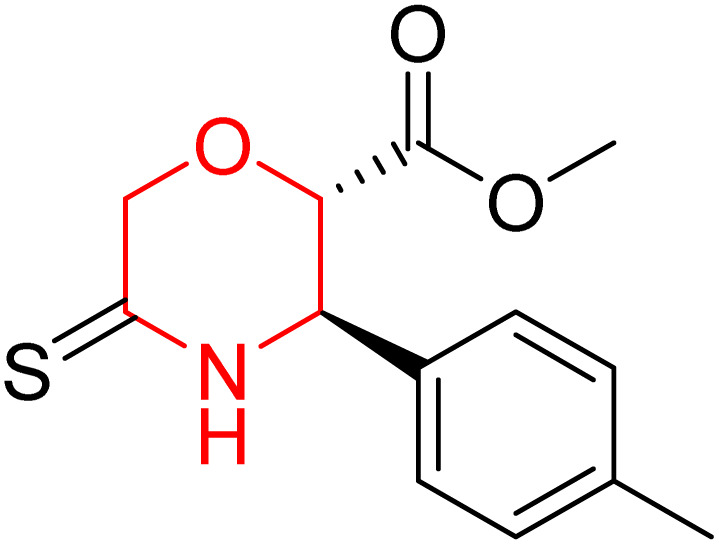	—	—	—	—	—	—	—	—
108	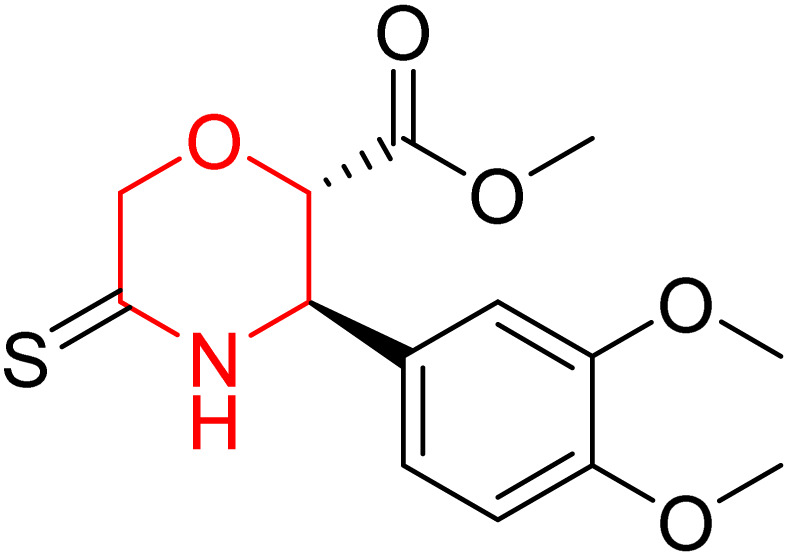	—	—	—	—	—	—	—	—
109	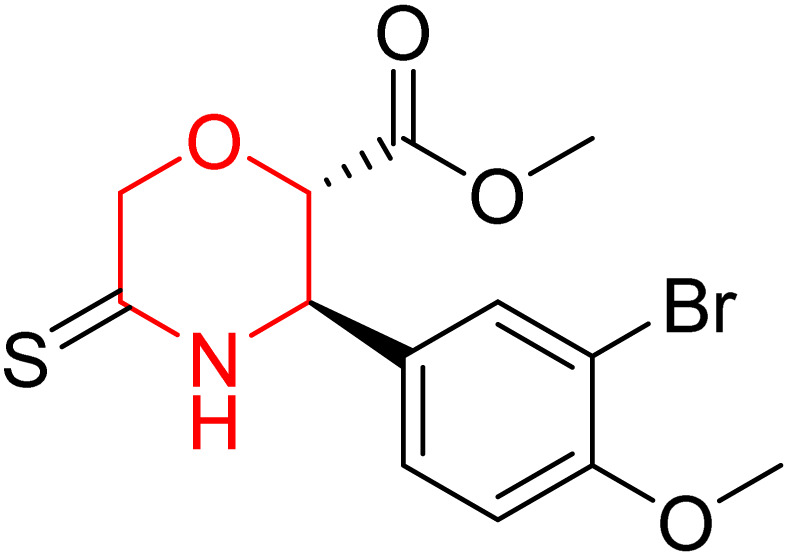	—	—	—	—	—	—	—	—
110	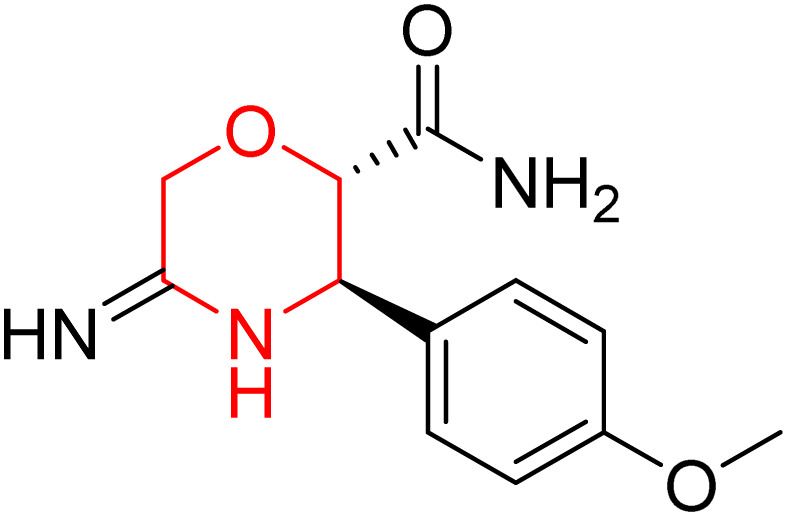	—	—	—	—	—	—	—	—
111	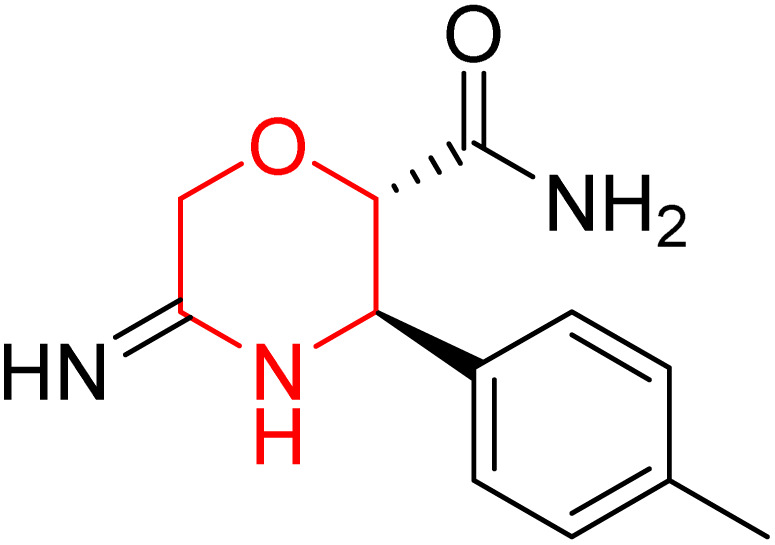	—	—	—	—	—	—	—	—
112	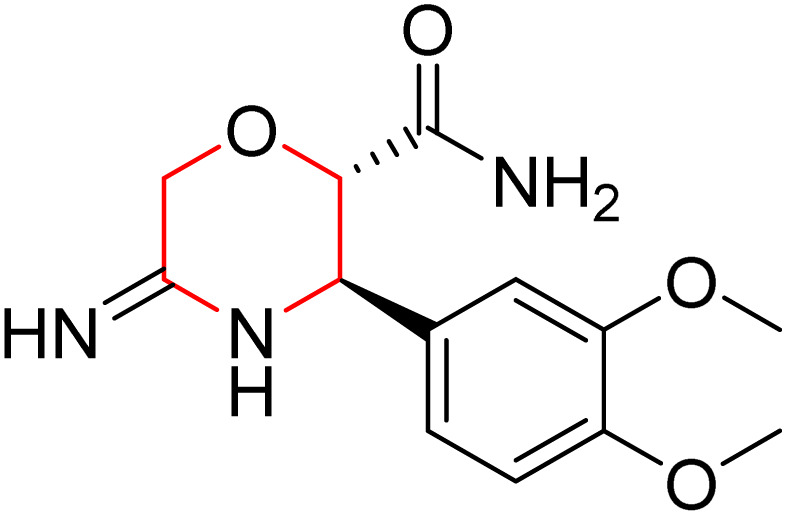	—	—	—	—	—	—	—	—
113	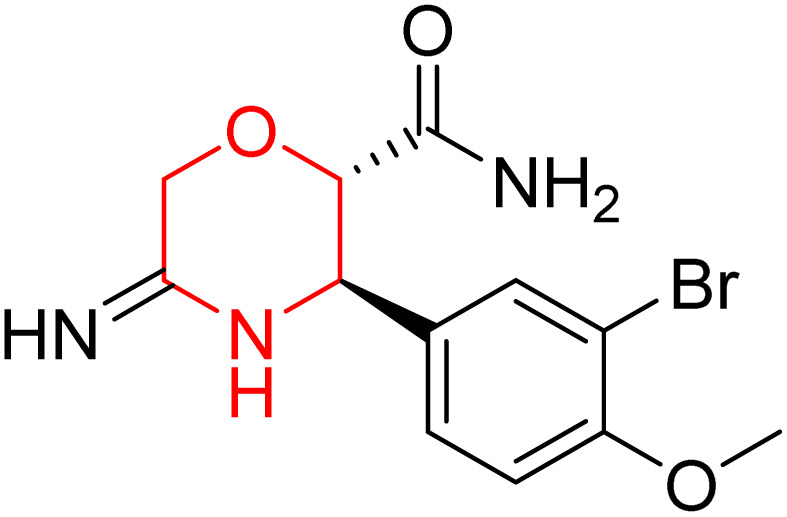	—	—	—	—	—	—	—	—
114	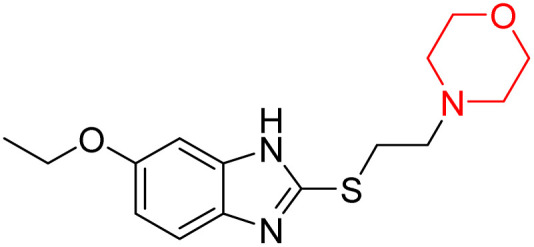	—	—	—	—	—	—	—	7.1
115	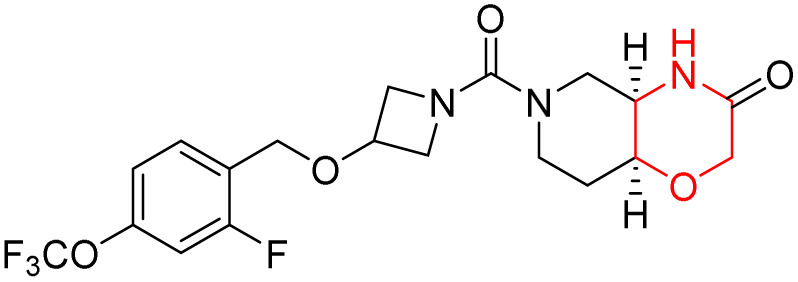	—	—	—	—	—	—	0.012 ± 0.001	—
116	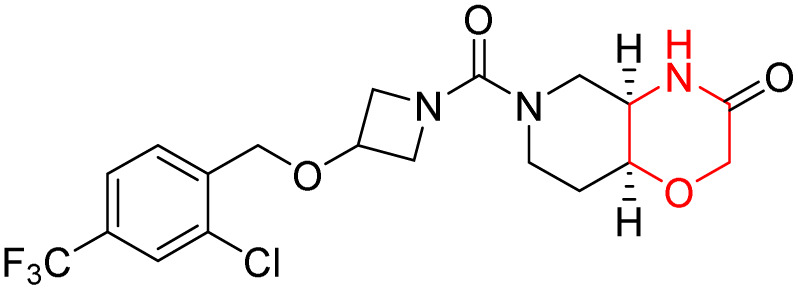	—	—	—	—	—	—	0.005 ± 0.002	—
117	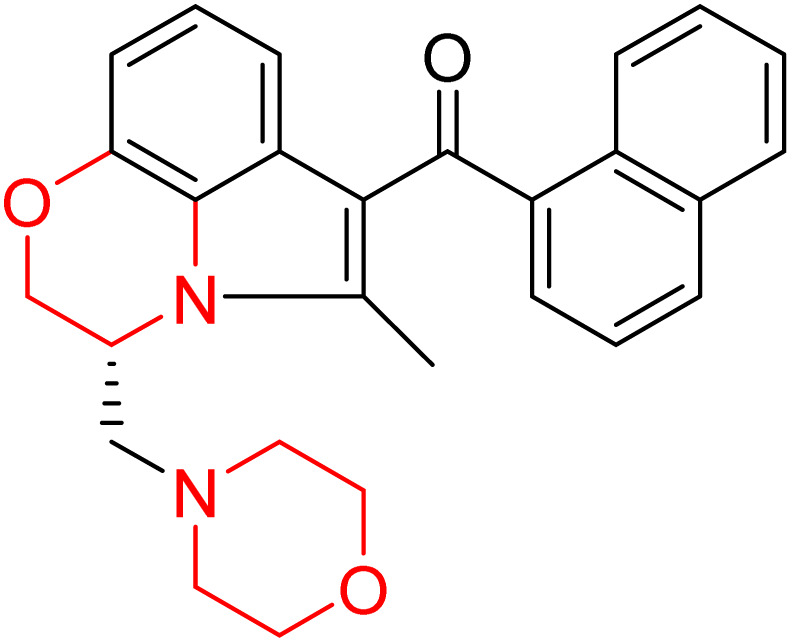	—	—	—	—	—	—	—	—

## 3 Discussion

The research studies emphasise that morpholine was identified as a structurally versatile and pharmacologically potent scaffold, which is capable of altering numerous CNS-related targets. The comparison of enzyme data reveals that morpholine-derived chalcones and benzothiazole-morpholine hybrids have a potent inhibitory effect on MAO-B (*e.g.*, compound 18, IC_50_ = 0.065 µM; compound 20, IC_50_ = 0.078 µM). This suggests that their selectivity indices (SI > 60) are on par with or better than those of standards like toloxatone and pargyline. The morpholine ring in the mTOR inhibitors can be modified to make it more selective and less prone to degradation in the body. For example, compound 97 is able to cross the blood–brain barrier without P-gp efflux, and it also inhibited mTOR strongly (*K*_i_ = 3.6 nM). The previous works very much describe morpholine as a versatile scaffold traversing enzyme inhibition, receptor modulation, and kinase targeting.

Some morpholine-derived CNS-active compounds reviewed in this article have been assessed for drug-likeness and ADMET-related properties using *in silico* prediction software, physicochemical characterization, and selected *in vitro* experiments, as described in the cited literature. These assessments include blood–brain barrier permeability (PAMPA-BBB and BOILED-Egg models), CNS multiparameter optimization (CNS MPO) scores, physicochemical properties, and *in silico* ADME prediction using software such as SwissADME. Taken together, these findings suggest that morpholine can be a useful structural element for the optimization of favorable CNS-related properties, such as balanced lipophilicity, acceptable polar surface area, and predicted brain penetration.

In terms of drug development, the translational utility of morpholine-derived scaffolds is reflected in the existence of morpholine-derived compounds such as viloxazine and reboxetine, which are morpholine-derived norepinephrine reuptake inhibitors and are currently in clinical use, as described in the cited literature. In contrast, most morpholine-derived compounds targeting MAO, cholinesterases, mTOR, BACE-1, MAGL, sigma-1 receptors, dopamine receptors, and cannabinoid receptors are still in the preclinical stage of development and are being evaluated for enzymatic potency, selectivity, and initial ADMET characterization.

However, these intriguing findings must be interpreted with a number of concerns that affect their practical applicability. Additional concerns of stability problems and drug–drug interactions were reported. Clearance profiles cannot be regarded as the most reliable. This suggests that further SAR tuning is aimed at reducing off-target metabolic liabilities. Even more important is the fact that morpholine-based entities continue to perform well in preclinical and *in vitro* tests. The majority are still in the early stages of development and have no obvious path to regulation or clinical trials. Efforts to improve metabolic stability, encourage BBB transport, and collect further *in vivo* safety data are necessary since these difficulties highlight the gap between potent biological activity and useful therapeutic efficacy.

Future morpholine scaffold placement is also influenced by advancements in neuroprotective drug design. Biomaterial-based strategies are one of such advancements that mimic the extracellular environment using synthetic or natural matrices. The ultimate aim is to deliver drugs to the central nervous system that not only keep their stability, but also enhance their absorption and integration into brain tissue.^42^ In the same way, glutathione (GSH) delivery systems are very attractive in models for Alzheimer's and Parkinson's diseases since they have the potential to reduce oxidative stress and mitochondrial dysfunction. A number of research works have suggested the idea of incorporation of such delivery innovations with small-molecule scaffolds.^43^ These novel concepts in the field reveal that neuroprotective medications that developed from the synergistic use of chemical scaffolds such as morpholine and advanced delivery technologies to practical relevance in future. The systemic state of Intellectual Property Rights (IPR) continues to impede innovation. It affects the speed at which novel morpholine-based agents may be acquired and their practical use.^44^ According to a recent bibliographic study on the subject, clearing the path through regulatory and intellectual property rights frameworks will enable these compounds to progress from being molecules in the research stage to being recognised as therapeutic candidates. All of these considerations together reinforce the idea that morpholine is a chemically versatile and pharmacologically potent scaffold, and it is able to modulate various CNS pathways. However, advancement of this into clinical practice will be contingent upon solving issues related to metabolism, demonstrating safety in long-term use, and incorporating delivery and regulatory aspects in the upcoming research work.

## 4 Conclusion

Morpholine has become one of the most valuable scaffolds in the CNS drug discovery because of its superior physicochemical properties and its ability to modulate a wide variety of targets. It targets kinases, dopamine receptors, cholinesterase, and MAO-B. Recent research gives very strong evidence of the potency and selectivity of the compounds across different systems. This reveals the potential of morpholine as a source of neurodegenerative diseases. This study emphasises all of the current synthetic methodologies, pharmacological data, and structure–activity relationships, underlining both the potential and the challenges with morpholine-based medicines. In order to determine whether morpholine derivatives might progress towards therapeutically meaningful neuroprotective medicines, future research should improve blood–brain barrier (BBB) delivery techniques, optimise the desinging of scaffold and provide *in vivo* validation.

## Conflicts of interest

There are no conflicts to declare.

## Data Availability

No primary research results, software or code have been included and no new data were generated or analysed as part of this review.
